# The *Capicua* C1 Domain Is Required for Full Activity of the CIC::DUX4 Fusion Oncoprotein

**DOI:** 10.1158/2767-9764.CRC-24-0348

**Published:** 2024-12-09

**Authors:** Cuyler Luck, Kyle A. Jacobs, Ross A. Okimoto

**Affiliations:** 1 Biomedical Sciences Graduate Program, University of California, San Francisco, San Francisco, California.; 2 Department of Medicine, University of California, San Francisco, San Francisco, California.; 3 Department of Cell and Tissue Biology, University of California, San Francisco, San Francisco, California.; 4 Helen Diller Comprehensive Cancer Center, University of California, San Francisco, San Francisco, California.

## Abstract

Rearrangements between genes can yield neomorphic fusions that drive oncogenesis. Fusion oncogenes are made up of fractional segments of the partner genes that comprise them, with each partner potentially contributing some of its own function to the nascent fusion oncoprotein. Clinically, fusion oncoproteins driving one diagnostic entity are typically clustered into a single molecular subset and are often treated a similar fashion. However, knowledge of where specific fusion breakpoints occur in partner genes, and the resulting retention of functional domains in the fusion, is an important determinant of fusion oncoprotein activity and may differ between patients. This study investigates these phenomena through the example of *capicua* (*CIC*)::*double homeobox 4* (*DUX4*), a fusion between transcriptional repressor *CIC* and *DUX4* genes, which drives an aggressive subset of undifferentiated round cell sarcoma. Using a harmonized dataset of more than 100 patient fusion breakpoints from the literature, we show that most *bona fide* CIC::DUX4 fusions retain the C1 domain, which is known to contribute to DNA binding by wild-type CIC. Mechanistically, deletion or mutation of the C1 domain reduces, but does not eliminate, the activation of *CIC* target genes by CIC::DUX4. We also find that expression of C1-deleted CIC::DUX4 is capable of exerting intermediate transformation-related phenotypes compared with those imparted by full-length CIC::DUX4 but was not sufficient for tumorigenesis in a subcutaneous mouse model. In summary, our results suggest a supercharging role for the C1 domain in the activity of CIC::DUX4.

**Significance:**

We show in mammalian settings that the capicua C1 functional domain is a supercharger for CIC::DUX4, a poorly studied fusion oncoprotein which drives a rare sarcoma with dismal outcomes.

## Introduction

When DNA replication and repair pathways fail to faithfully process genomic DNA, one possible outcome is the formation of fusion genes comprised of portions of two individual partner genes that have been rearranged into a single gene. In the case of many cancer types, such events give rise to fusion oncogenes, which are transcribed and translated into fusion oncoproteins that deviate from the behavior of their wild-type (WT) partners. Transcription factor (TF) fusions represent a subset of fusion oncogenes that drive multiple tumor types, including sarcomas, through chromatin remodeling, enhancer hijacking, and target gene dysregulation (1–6). In such tumor types, the TF fusion is often one of few, if not the sole, genetic alterations present (7, 8), suggesting they are key driver events.

Although fusion oncoproteins are neomorphic entities not observed in healthy cells, the functional domains guiding their form, function, and regulation are inherited from the normal partner genes that comprise them. This can directly determine how the fusion executes its oncogenic function: for example, PAX3::FOXO1 (fusion-positive rhabdomyosarcoma) binds DNA at sites determined by the *PAX3* DNA-binding domains, whereas *FOXO1* provides transactivating capacity (9, 10). Beyond directly shaping output, partner gene domains can also determine regulation of the fusion, as is the case for ERK-mediated degradation of *capicua* (*CIC*)::*double homeobox 4* (*DUX4*; CIC-rearranged sarcoma) due to retention of the *CIC* ERK-binding domain in the fusion oncoprotein (11, 12). Due to the critical roles they play in the activity of the overall fusion, knowing which functional domains are retained from partner genes can be informative in understanding how a fusion acts or can be acted upon. Importantly, fusion breakpoints and transcripts can be variable between patients with the same tumor types (13–15), which may result in different exons and potentially variable functional domains being retained in any individual fusion. Despite these possible molecular differences, it is often assumed that all fusion oncoproteins made up of a given set of partner genes operate in a similar fashion.

Particularly interesting classes of TF fusions are those involving rearrangements of *CIC* with one of several 3′ partner genes, typically *DUX4* (but also including *NUTM1*, *LEUTX*, and *FOXO4*). WT *CIC* is a transcriptional repressor discovered in *Drosophila* which is involved in developmental processes (16, 17) and acts as a tumor and metastasis suppressor predominantly through the repression of *PEA3* (*ETV1*, *ETV4*, and *ETV5*) family members and negative MAPK pathway (*DUSP4/6*) regulators (18–21). As a tumor suppressor, WT *CIC* is genetically deleted, mutated, or otherwise functionally inactivated in several types of cancers as we have previously reviewed (22). Interestingly, when fused to *DUX4*, CIC::DUX4 becomes an oncogenic neomorphic transcriptional activator of CIC target genes (6, 23–25) and drives undifferentiated round cell sarcoma with a dismal prognosis (26) and limited treatment options. This contrast between WT CIC acting as a tumor suppressor and CIC::DUX4 being an oncogene provides a rare opportunity to take knowledge learned from the natural evolution of CIC inactivation in WT CIC cancers and apply those strategies to potentially disable the CIC::DUX4 fusion oncoprotein.

We have taken a particular interest in the C1 domain of *CIC*, a C-terminal DNA-binding domain that cooperates with the HMG box to engage DNA at target sites, as has been best shown by Forés and colleagues, among others (bioRxiv 2022.03.28.485992; refs. 18, 27). Their work highlighted that the C1 domain is a mutational hot spot in glioma and showed that in *Drosophila*, truncation of the C1 domain in a synthetic fly–human CIC::DUX4 construct partially rescues phenotypes imparted by the expression of the fully intact fusion. However, the role of the C1 domain in CIC::DUX4 activity has not been formally tested in a human or mammalian context. The C1 domain is retained in the fusion transcripts of the few existing patient-derived CIC::DUX4 sarcoma cell lines (6, 28–30), but there has been no large-scale comprehensive analysis of CIC::DUX4 patient fusion breakpoints and which functional domains are retained in their fusion oncoproteins. This limits our understanding of how frequently the C1 domain is included in CIC::DUX4 oncoproteins in patients, making generalizations to patient biology difficult.

In this study, we leverage the first harmonized database of CIC-rearranged breakpoints from patients along with cellular models to interrogate the role of the C1 domain in mammalian CIC::DUX4 activity. We find that the C1 domain is retained in the majority of *bona fide* CIC::DUX4 patient fusions, with the *CIC* breakpoint typically constrained to a relatively small C-terminal region. Deletion or mutation of the C1 domain attenuated, but did not entirely eliminate, the activation of target genes in multiple cell models with ectopic expression of CIC::DUX4. This translated to phenotypic outcomes, in which C1-deleted CIC::DUX4 was capable of driving intermediate phenotypes including partially blocking myogenic differentiation. However, the expression of C1-deleted CIC::DUX4 was not capable of forming tumors in an *in vivo* subcutaneous implantation model, in contrast to expression of the full-length CIC::DUX4 fusion oncoprotein. Taken together, these results suggest that the C1 domain is necessary for maximal activity of the CIC::DUX4 fusion oncoprotein in mammalian systems.

## Materials and Methods

### Cell culture

293T (female, CRL-3216, RRID: CVCL_0063), NIH/3T3 (male, CRL-1658, RRID: CVCL_0594), and C2C12 (female, CRL-1772, RRID: CVCL_0188) cells were purchased from ATCC (293T: September 2021; NIH/3T3: September 2022; C2C12: January 2023) and short tandem repeat validated using the FTA Human (135-XV) or Mouse (137-XV) Authentication Services (ATCC). All cell lines were grown at 37°C under humidified conditions in 5% CO_2_ incubators in DMEM with high glucose, L-glutamine, and sodium pyruvate (SH30243.02, Cytiva) plus 10% FBS, 100 U/mL penicillin, and 100 µg/mL streptomycin. Cell lines were routinely tested for *Mycoplasma* using the e-Myco plus Mycoplasma PCR Detection Kit (Boca Scientific), typically included after completion of experiments with a given thawed cell line. Cell lines were typically grown for approximately 48 hours after thawing before being used for experiments and were typically passaged for no longer than 1 month before being discarded in favor of a lower-passage vial. Live cells in culture were imaged using a Moticam Pro 205A camera mounted on a trinocular inverted microscope (VWR 89404-462) or using an Olympus EP50 camera mounted on an Olympus CKX53 microscope.

### CIC-fusion breakpoint database and analysis

Breakpoint coordinates for CIC-rearranged fusion genes described in the literature were manually aligned to reference sequences for *CIC* (NM_015125.5), *DUX4* (NM_001306068.3), *NUTM1* (NM_001284292.2), *LEUTX* (NM_001382345.1), and *FOXO4* (NM_005938.4). When ambiguous bases existed at the breakpoint junction that could not be definitively attributed to either partner, they were assigned to CIC. The full dataset also includes exon-level information for 26 breakpoints where the precise nucleotide coordinates could not be identified, but we advise caution interpreting this data as not all authors use identical reference sequences and exon numbering is not always consistent across references. Although we have done our best to harmonize all data, please refer to the original publications for the most accurate details for specific cases. The full dataset comprising of 108 nucleotide-level and 26 exon-level breakpoints is available for download in Supplementary Dataset S1. Functional domain annotations in scatterplots of breakpoint locations were derived from refs. 19, 27, 31, and 32. The codes used to visualize the data are available at https://github.com/cuylerluck/C1_domain_2024/ (33–36).

### Cloning

HA-CIC::UTR fusion, C1 and HMG domain deletion, and point mutant constructs were cloned from pcDNA3.1-HA-CIC::DUX4 [a gift from Takuro Nakamura (6)] using the Q5 Site-Directed Mutagenesis Kit (NEB E0554S) with the primers described in Supplementary Dataset S2. The residues deleted for the C1 domain (R1464-M1519, NM_015125.5) and HMG box (I200 – K268, NM_015125.5) deletion mutants were determined from Forés and colleagues (27) and UniProt (RRID: SCR_002380) entry Q96RK0, respectively. The empty vector (EV) construct used for 293T experiments was pCMV-Neo-Bam, which has the same major functional elements as pcDNA3.1 with the exception of an HSV TK promoter for the neoresistance gene in place of an SV40 promoter. pCMV-Neo-Bam was a gift from Bert Vogelstein (Addgene, plasmid #16440; http://n2t.net/addgene:16440; RRID: Addgene_16440; ref. 37). C1-deleted pMYs-CIC::DUX4-IRES-EGFP was cloned from pMYs-CIC::DUX4-IRES-EGFP [a gift from Takuro Nakamura (38) and Michael Kyba (25)] using the Q5 Site-Directed Mutagenesis Kit (NEB E0554S) with the primers described in Supplementary Dataset S2. The corresponding EV pMY-IRES-EGFP was a gift from Louis Ates and Teunis Geijtenbeek (Addgene, plasmid #163361; http://n2t.net/addgene:163361; RRID: Addgene_163361; ref. 39). Plasmid sequences were verified via whole-plasmid sequencing by Plasmidsaurus/Primordium Labs using Oxford Nanopore Technologies with custom analysis and annotation. Select regions were also verified by Sanger sequencing (Quintara Biosciences).

### Plasmid transfections

For overexpression experiments, typically 1.5 μg of plasmid was reverse transfected into 300,000 293T cells in a well of a six-well plate using Opti-MEM (Gibco) and FuGENE 6 (Promega), at a 2:1 FuGENE:DNA ratio. Transfected cells were typically processed for downstream analysis after approximately 48 hours. For retrovirus generation, 293T cells were forward transfected using the same reagents.

### Western blot

Adherent cells were washed 3× with Dulbecco's Phosphate Buffered Saline (DPBS), scraped with RIPA buffer supplemented with Halt protease and phosphatase inhibitors (Thermo Fisher Scientific), and incubated on ice for at least 15 minutes. Cell suspensions were then sonicated and centrifuged before the supernatant was collected for analysis. Protein samples were normalized, boiled, separated by denaturing electrophoresis on Criterion TGX 4–15% gels (Bio-Rad), transferred to nitrocellulose membranes (Bio-Rad Trans-Blot Turbo), evaluated for transfer by Ponceau S (Sigma-Aldrich) staining, and blocked in 5% BSA in TBS-T for at least 1 hour. Precision Plus Protein Dual Color Standards (Bio-Rad) were used as molecular weight markers. Blots were horizontally cut to use for separate targets, and separate blots from identically loaded samples run at the same time were used for targets of similar sizes as described in figure legends where appropriate (per-blot details and full Ponceau S loading controls are shown in Supplementary Dataset S3). Primary antibodies were diluted in blocking buffer and incubated overnight followed by washing with TBS-T, and secondary antibodies were similarly diluted and incubated for 1 hour. After washing with TBS-T, imaging was performed on a Bio-Rad ChemiDoc Touch using ECL Prime reagent (Millipore Sigma) by briefly drying the blot, submerging the blot in ECL Prime mixture, dabbing excess solution off, and imaging. When required, brightness/contrast was adjusted either on the ChemiDoc or using Image Lab (Bio-Rad). The antibodies used and their dilutions were HSP90 (Cell Signaling Technology 4874S, 1:1,000–1:3,000), HA-tag clone C29F4 (Cell Signaling Technology 3724S, 1:1,000), ETV5 (Cell Signaling Technology 16274S, 1:1,000), DUX4 clone P4H2 (Invitrogen MA5-16147, 1:1,000–1:2000), GFP (Cell Signaling Technology 2956S, 1:1,000–1:5,000), Myogenin clone F5D (Invitrogen 14-5643-82, 1:2,000), horseradish peroxidase–linked rabbit IgG (Cell Signaling Technology 7074, 1:3,000), and horseradish peroxidase–linked mouse IgG (Cell Signaling Technology 7076, 1:3,000). Although HSP90 is shown for most blots as a presence/absence loading control, it was not optimized for quantitation. For better analysis of quantitation between lanes, please refer to the corresponding Ponceau stains in Supplementary Dataset S3.

### RT-qPCR

RNA was extracted from adherent cells with the RNeasy Mini Kit (Qiagen) as per the manufacturer’s protocol. Approximately 1 µg RNA per sample was used as input to the SensiFAST cDNA Synthesis Kit (Bioline). RT-qPCR was performed in technical quadruplicate with TaqMan Fast Advanced Master Mix (Applied Biosystems) and TaqMan probes on an Applied Biosystems StepOnePlus. The probes used were *GAPDH* (Hs02758991_g1), *ETV1* (Hs00951951_m1), *ETV4* (Hs00383361_g1), *ETV5* (Hs00927557_m1), and *DUSP6* (Hs04329643_s1). Gene expression was normalized to GAPDH using the 2^(Ct housekeeping − Ct experimental gene)^ method. To combine replicates for statistical analysis, replicates were first individually normalized with 0% defined as the average of the EV condition and 100% defined as the average of the HA-CIC::DUX4 condition. Then, technical quadruplicates were averaged for each replicate and aggregated. Statistical analysis was performed using one-way ANOVA and Šidák’s multiple comparisons test. GraphPad Prism (version 10.2.3, RRID: SCR_002798) was used for statistical analysis.

### Retroviral transductions and clonal cell line generation

Ecotropic retrovirus was generated in 293T cells by co-transfection of one of the transfer vectors (pMY-IRES-EGFP, pMYs-CIC::DUX4-IRES-EGFP, or pMYs-CIC::DUX4-IRES-EGFP dC1) and the packaging vector pCL-Eco (Novus Biologicals NBP2-29540). NIH/3T3 or C2C12 cells were infected by the addition of a viral supernatant with 10 µg/mL Polybrene (Millipore Sigma) directly on top of cells and were bulk sorted for EGFP^+^ cells 48 hours after infection with a FACSAria II (BD Biosciences). To generate clonal cell lines, a FACSAria II or III (BD Biosciences) was used to further sort single EGFP^+^ cells into individual wells of a 96-well plate. Wells with a single colony confirmed by microscopic inspection were expanded for freezing and evaluated for transgene expression by Western blot before further use.

### Luciferase reporter assay

In a 96-well plate, 10,000 293T cells per well were reverse transfected as described above with 50 ng each of experimental plasmid (EV or HA-CIC::DUX4/mutants) and a GoClone *ETV5* promoter-RenSP construct (SwitchGear Genomics S722373) containing the consensus CIC binding sequence TGAATGAA. Transfections were performed in technical quadruplicate. After approximately 48 hours, cells were processed with the LightSwitch Assay Kit (Active Motif LS010), and luciferase signal was read for 1.5 seconds on a SpectraMax M5 (Molecular Devices). To combine replicates for statistical analysis, replicates were first individually normalized with 0% defined as the average of the EV condition and 100% defined as the average of the HA-CIC::DUX4 condition. Then, technical quadruplicates were averaged for each replicate and aggregated. Statistical analysis was performed using one-way ANOVA and Šidák’s multiple comparisons test. GraphPad Prism (version 10.2.3, RRID: SCR_002798) was used for statistical analysis.

### Immunofluorescence

For transfected 293T, cells were first reverse transfected as described above with appropriate plasmids. The next day, transfected cells were passaged onto collagen I-coated (50 µg/mL, Corning 354236) 18-mm coverslips. Approximately 48 hours after transfection, cells were fixed with 4% paraformaldehyde in PBS for 10 minutes at 37°C, briefly washed once with DPBS, quenched with 100 mmol/L glycine for 30 minutes at room temperature, briefly washed twice with DPBS, permeabilized with 0.2% Triton X-100 for 15 minutes at room temperature, washed three times with DPBS, and blocked in 2% BSA overnight at 4°C. Coverslips were incubated with primary antibody (HA-tag clone C29F4, Cell Signaling Technology 3724S, dilution 1:300) for 2 hours at room temperature, washed three times with DPBS, and again incubated with a secondary mixture of 1:10,000 rhodamine phalloidin (Invitrogen R415), 1:1000 DAPI (Thermo Fisher Scientific D1306), and 1:300 anti–rabbit IgG Alexa 647 (Invitrogen A-21245) for 1 hour at room temperature. Coverslips were washed three times with DPBS and mounted in ProLong Glass (Invitrogen P36984). Cells were imaged on a Yokogawa CSU-X1 spinning disk confocal on a Nikon Ti-E microscope with a 60×/1.45NA lens (Nikon) using NIS-Elements AR (v5.21.03, Nikon) software at consistent exposure times and laser powers. Images were processed using FIJI/ImageJ (https://github.com/fiji/fiji). Briefly, all confocal planes were converted to single images by maximum intensity projection, and brightness/contrast was consistently adjusted between all images.

For transduced clonal NIH/3T3, cells were seeded onto poly-L-lysine (0.01%, Sigma-Aldrich P4707-50ML) coated coverslips and allowed to adhere overnight. Cells were then similarly prepared as above but blocked for 1 hour at room temperature, prepared without a primary antibody step, and used 1:400 rhodamine phalloidin (Invitrogen R415) and 1:2,000 DAPI (Thermo Fisher Scientific D1306). Cells were imaged on a ZEISS Axio Imager 2 with ZEISS ZEN 2 (blue edition, version 2.0.0.0) software and a 20×/0.8 Plan-Apochromat air objective (ZEISS) at consistent exposure times and laser powers, with direct detection of EGFP fluorescence. Images were processed as described above, without the maximum intensity projection step.

### RNA sequencing and analysis

For NIH/3T3 clones, 150,000 cells per cell line were seeded in wells of a six-well plate and allowed to grow for approximately 48 hours. After 48 hours, RNA was extracted using the RNeasy Mini Kit (Qiagen) including an on-column DNase digest (Qiagen) and an additional short Buffer RPE wash. RNA samples were additionally processed with the Monarch RNA Cleanup Kit (NEB), analyzed for integrity with a TapeStation (Agilent), and submitted to Novogene for library preparation and sequencing. Samples were polyA enriched, and directional libraries were prepared using the NEBNext Ultra II Directional Library Prep Kit for Illumina (NEB). Libraries were PE150 sequenced on a NovaSeq 6000 (Illumina) with approximately 10 Gb of data per sample. C2C12 clones were similarly grown and processed but with 75,000 cells per cell line seeded, no additional short Buffer RPE wash, and approximately 11 to 14 Gb of data per sample.

FASTQ files showed no concerning quality indications per both the Novogene report and our own analysis with FastQC (RRID: SCR_014583; ref. 40). Full code describing processing and analysis of the data is available at https://github.com/cuylerluck/C1_domain_2024/. Briefly, FASTQ files were aligned to the mouse GRCm39 genome with STAR (RRID: SCR_004463; ref. 41), including quantitation of gene counts using the option quantMode GeneCounts. Uniquely mapped read rates were between 90% and 94% for all samples. The grep function was used to manually verify the presence of proper transgenes in each sample. A custom R script (version 4.2.2; refs. 42–49), also available at https://github.com/cuylerluck/C1_domain_2024/, was used to separately perform differential gene expression analysis for NIH/3T3 and C2C12 samples. Briefly, column 4 from STAR GeneCount output was merged between all samples. Then, edgeR (version 3.40.2; RRID: SCR_012802; refs. 50–53) was used for differential expression analysis with a GLM method and quasi-likelihood F-tests, with resulting *P*-values then FDR corrected. The log_2_ (counts per million) values were extracted from the trimmed mean of M-values (TMM)-normalized dataset using the cpm function. Gene list functional profiling was performed with the gProfiler2 package (54) with multiple testing correction using the built-in gSCS method. The differential expression output for the CD4 versus EV, dC1 versus EV, and dC1 versus CD4 comparisons for both NIH/3T3 and C2C12 samples is available in Supplementary Datasets S4 to S9. The gene list functional profiling output from both NIH/3T3 and C2C12 samples is available in Supplementary Datasets S10 to S15. The raw FASTQ files, log_2_ (cpm) data, and counts data have been deposited in NCBI’s Gene Expression Omnibus (GEO; RRID: SCR_005012; ref. 55) and are accessible through GEO Series accession numbers GSE269295 (https://www.ncbi.nlm.nih.gov/geo/query/acc.cgi?acc=GSE269295) and GSE279546 (https://www.ncbi.nlm.nih.gov/geo/query/acc.cgi?acc=GSE279546) for the NIH/3T3 and C2C12 data, respectively.

### Crystal violet assay

Approximately 10,000 (NIH/3T3 clones) or 7,500 (C2C12 clones) cells were seeded in individual wells of a six-well plate and allowed to grow for approximately 96 hours. Media were then removed, and cells were gently washed once with DPBS before fixation with 4% paraformaldehyde at 37°C for 10 minutes. Wells were then washed three times with water and stained with 0.05% crystal violet (Sigma-Aldrich) for 30 minutes. The staining solution was then removed, and wells were washed three times with water before air-drying. Plates were imaged with a ChemiDoc (Bio-Rad) once dry. For quantitation, FIJI was used to measure the mean signal intensity within each well, which was multiplied by the area measured to calculate the total signal intensity per well. Background signal was calculated by multiplying the same area value by the mean signal measured from an image area outside the plate. Then, the background signal was subtracted from the sample signal, and the results were plotted using GraphPad Prism (version 10.2.3, RRID: SCR_002798).

### CellTiter-Glo assay

A total of 350 cells per well were seeded in 100 μL of media within triplicate interior wells of a white-walled opaque-bottomed 96-well plate, with 100 μL of media alone added to all other wells of the plate. Three identical plates were prepared per experiment, to be harvested at different time points (approximately 1, 72, and 96 hours postseeding). At appropriate time points, plates were processed using CellTiter-Glo reagent (Promega) as per the manufacturer’s protocol. Briefly, plates were allowed to equilibrate to room temperature for 30 minutes, and 100 μL of CellTiter-Glo reagent was then added to all cell-containing wells and three media-only wells. Plates were mixed for 2 minutes on an orbital shaker and allowed to stabilize for 10 minutes, and then luminescence was read for 500 ms on a SpectraMax M5 (Molecular Devices). Within each experiment, the signal for each technical replicate was normalized to that of the same well in the 1 hour postseeding plate, and then technical replicates were averaged. The mean and standard deviation of the normalized and averaged data from the independent experiments were then plotted using GraphPad Prism (version 10.2.3, RRID: SCR_002798).

### C2C12 differentiation assay

Clonal C2C12 cell lines were seeded at 175,000 cells per well in wells of identically prepared six-well plates, one plate per time point. The next day, media were removed and gently rinsed once with DPBS, and differentiation media were added [growth DMEM as defined above, but with 2% horse serum (Thermo Fisher Scientific 16050130) in place of FBS]. The day 0 plate was immediately imaged and harvested for protein as described above. Differentiation media were changed 2 days after differentiation began, and the day 4 plate was imaged. The day 4 plate was imaged and harvested another 2 days later, and differentiation media were replaced on the day 6 plate. Finally, the day 6 plate was imaged and harvested after another 2 days. Protein samples were analyzed by Western blot as described above.

For the siCIC + differentiation experiment, 175,000 clonal C2C12 cells per well were plated in six-well plates. The next day, one plate was briefly rinsed once with DPBS, switched to differentiation media, and immediately harvested for protein. The other plate was briefly rinsed once with DPBS and switched to differentiation media, and cells were forward transfected with 100 pmol of appropriate siRNA Dharmacon ON-TARGETplus SMARTpools, either nontargeting (Horizon D-001810-10-05) or human siCIC (Horizon L-015185-01-0020), using Lipofectamine RNAiMAX (Invitrogen). Differentiation media were then changed 2 and 4 days after transfection, and cells were harvested for protein 5 days after infection. Protein samples were analyzed by Western blot as described above. The sequences for the siRNA pools were as follows: nontargeting: UGG​UUU​ACA​UGU​CGA​CUA​A, UGG​UUU​ACA​UGU​UGU​GUG​A, UGG​UUU​ACA​UGU​UUU​CUG​A, and UGG​UUU​ACA​UGU​UUU​CCU​A; human siCIC: GCU​UAG​UGU​AUU​CGG​ACA​A, CGG​CGC​AAG​AGA​CCC​GAA​A, GAG​AAG​CCG​CAA​UGA​GCG​A, and CGA​GUG​AUG​AGG​AGC​GCA​U. None of the human siCIC sequences are perfect matches for the mouse short *CIC* isoform (NM_027882.4), each having one to three mismatches with mouse *CIC*.

For the siGFP^+^ differentiation experiment, the same protocol as for the siCIC experiment was followed but using Dharmacon ON-TARGETplus Nontargeting Control siRNA #1 (Horizon D-001810-01-20, target sequence UGG​UUU​ACA​UGU​CGA​CUA​A) and Dharmacon GFP Duplex I siRNA (Horizon P-002048-01-20, target sequence GCA​AGC​TGA​CCC​TGA​AGT​TC). Wells were additionally imaged on day 5 prior to protein harvesting.

### Hanging drop assay

Cells were diluted to 1.5e6 viable cells/mL, and 5-μL droplets (7,500 cells per droplet) were placed on the underside of the lid of a 10-cm tissue culture dish filled with 10 mL of sterile DPBS. Twelve droplets were placed per plate. Droplets were incubated for 24 hours, after which the lid was inverted droplet side up and imaged using a Moticam Pro 205A camera mounted on a trinocular inverted microscope (VWR 89404-462).

### *In vivo* tumorigenesis

Five- to six-week-old female nude (NU/J) mice were purchased from The Jackson Laboratory (RRID: IMSR_JAX:002019). Specific pathogen-free conditions and facilities were approved by the American Association for Accreditation of Laboratory Animal Care. Mouse experiments followed protocol AN194620-01C and were reviewed and approved by the University of California, San Francisco Institutional Animal Care and Use Committee. For subcutaneous flank injection, cell lines were prepared to 8 million cells per 500 μL DPBS, and 25 μL of cell suspension was mixed 1:1 with Matrigel (Corning 356234) to achieve 400,000 cells per 50 μL injection. For each of the three cell lines tested, five mice per group were anesthetized with isoflurane and subcutaneously injected in both flanks. Mice were monitored for tumor size three times a week starting 4 days postinjection, with measurable lesions evaluated with a digital caliper. Tumor volume was calculated by the (pi/6) × length × width × height method. The R script and raw data used to produce the plot of tumor size versus time are available at https://github.com/cuylerluck/C1_domain_2024/. Upon sacrifice, collectible tumor tissue was resected from euthanized mice and stored on ice, quickly frozen on dry ice upon return to the lab, and stored at −80°C.

Protein lysates were collected from tumor tissue by mortar and pestle in RIPA buffer supplemented with protease and phosphatase inhibitors, followed by sonication and centrifugation as above. Cell line control lysate was prepared from cells plated for 48 hours as described above. Western blotting was performed as described above.

For RNA extraction, tumor tissue was homogenized using a Precellys Evolution (Bertin Technologies) with High Impact Zirconium Beads (Benchmark Scientific D1032-30). RNA was then extracted using the RNeasy Mini Kit (Qiagen) including an on-column DNase digest (Qiagen), and 500 ng RNA per sample was used as input to the SensiFAST cDNA Synthesis Kit (Bioline), including RT controls. Cell line control cDNA was similarly prepared from cells plated for 48 hours. PCR was performed on the cDNA samples using Phusion polymerase (NEB). EGFP reactions used an HF buffer, 66°C annealing temperature, 35 cycles, and primers EGFP_FWD (5′ taa​acg​gcc​aca​agt​tca​gcg 3′) and EGFP_REV (5′ cag​ctc​gat​gcg​gtt​cac​cag 3′). CIC::DUX4 reactions used a GC buffer, annealing temperature 68°C, 40 cycles, and primers CIC_FWD (5′ aga​agc​cga​gga​cgt​gct​tgg 3′) and DUX4_REV (5′ gtt​gcg​cct​gct​gca​gaa​ac 3′). PCR products were electrophoresed on a 2% agarose gel made with ethidium bromide (Thermo Fisher Scientific) and imaged on a ChemiDoc Touch (Bio-Rad).

### Data availability

The RNA sequencing (RNA-seq) data generated in this study are publicly available in GEO at GSE269295 and GSE279546. The CIC-fusion breakpoint database is available in the Supplementary Materials.

## Results

### The majority of *bona fide* CIC::DUX4 fusions in patient tumors retain the C1 domain

**Figure 1:**
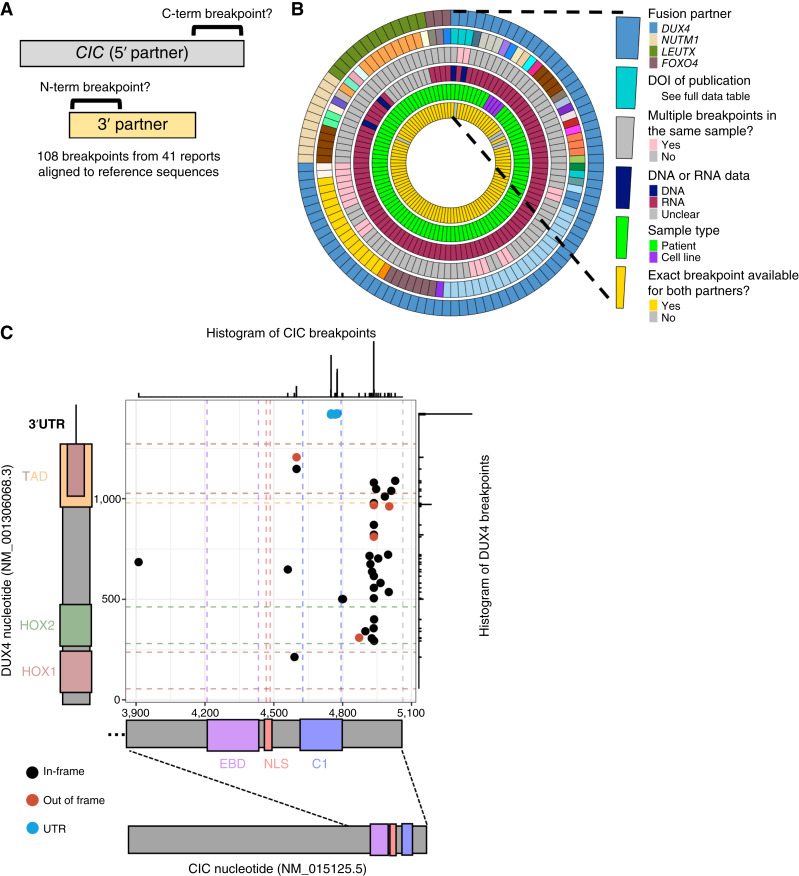
The CIC C1 domain is retained in most *bona fide* CIC::DUX4 fusion transcripts. **A,** Schematic describing harmonization of patient breakpoint data. **B,** Circos plot of all 108 nucleotide-level breakpoints gathered for CIC-fusion genes. **C,** Scatterplot of breakpoint coordinates from 74 CIC::DUX4 transcripts in which the data source was RNA and both partner breakpoints were available. Each data point is one breakpoint; histograms are intended to aid with overplotting. EBD: ERK-binding domain; C1, C1 domain; HOX1/2, homeodomains 1/2; NLS, nuclear localization signal; TAD, two overlapping putative transactivation regions identified in *DUX4*.

We first hypothesized that if the C1 domain is functionally important for human sarcomagenesis, then the *CIC* breakpoints observed in CIC-fusion transcripts would likely nonrandomly occur after the 3′ end of the C1 domain, preserving it in the resulting fusion oncoprotein. To the best of our knowledge, no comprehensive databases of CIC-fusion breakpoints exist, and reports describing CIC-fusion transcripts often use different reference sequences, complicating analysis. To overcome this challenge, we trawled the literature for reports of any CIC-rearranged fusion transcripts from patients, manually aligning all collected breakpoints to reference sequences (Fig. 1A). We were able to identify 108 nucleotide-level breakpoints from 41 different publications for four different *CIC* fusions (*DUX4*, *NUTM1*, *LEUTX*, and *FOXO4*; Fig. 1B), with an additional 26 exon-level breakpoints (full dataset available in Supplementary Dataset S1). For further detailed analysis, we focused on breakpoints derived from RNA data in which information on both partners were available. Interestingly, although *CIC* breakpoints for *DUX4*, *LEUTX*, and *FOXO4* fusions were largely at the far C-terminal end of the gene, those for *NUTM1* fusions were often relatively earlier in the coding sequence, including upstream of the C1 domain (Supplementary Fig. S1A). However, low sample numbers for the exceptionally rare non-*DUX4* fusions limited the generalizations that could be drawn for *LEUTX*, *FOXO4*, and *NUTM1* fusions.

As CIC::DUX4 fusions had the most breakpoints to analyze, we next focused on the 74 nucleotide-level CIC::DUX4 breakpoints which met the aforementioned selection criteria. Interestingly, we observed a large number of *CIC* breakpoints in CIC::DUX4 fusions that occurred within the C1 domain (*n* = 32 of 74, 43%), which we would expect to disrupt the C1 domain function (Supplementary Fig. S1A). However, closer analysis of the *DUX4* breakpoint in these samples indicated that these fusions are actually between *CIC* and the 3′-UTR of *DUX4*, which is consistent with prior observations of these cases in their primary reports (Fig. 1C; refs. 56–60). These “CIC::UTR” fusions have been described to often have stop codons shortly after the breakpoint, leading to a truncated CIC protein lacking a C-terminal region infringing on the C1 domain, which has been hypothesized to explain their apparent tumorigenic nature (56). To model this phenomenon, we used an HA-CIC::DUX4 plasmid (6) to clone one HA-CIC::UTR fusion based on case number 4 from Cocchi and colleagues (Supplementary Fig. S1B; Supplementary Dataset S2; ref. 59). We found that although its expression in 293T cells significantly increased the transcription of some known CIC target genes compared with the negative control, the magnitude of change in gene expression was exceptionally small compared with that observed following transfection of a full-length HA-CIC::DUX4 (Supplementary Fig. S1C and S1D).

Of the remaining 42 CIC::DUX4 breakpoints, we determined the majority to be genuine in-frame fusions (*n* = 37 of 42, 88%). Almost all of these *bona fide* CIC::DUX4 fusions retained the C1 domain, with the *CIC* breakpoint occurring after the 3′ end of the C1 domain in 32 of 37 *bona fide* CIC::DUX4 breakpoints (86%; Fig. 1C). This is exceptionally constrained compared with the distribution of *DUX4* breakpoints in the same samples, where the *DUX4* breakpoints are observed to occur almost anywhere between the HOX1 DNA-binding domain and the C-terminal region previously described as important for the recruitment of p300 and transactivation capacity (Fig. 1C; refs. 31, 61). Given that the extremely narrow C-terminal window where most *CIC* breakpoints occur in *bona fide* CIC::DUX4 fusions results in retention of the C1 domain in the fusion oncoprotein, we interpret this to suggest a likely functional contribution of the C1 domain to the activity of the fusion in a human context.

### Deletion or mutation of the C1 domain partially, but not completely, impairs target gene induction by CIC::DUX4 in 293T cells

**Figure 2:**
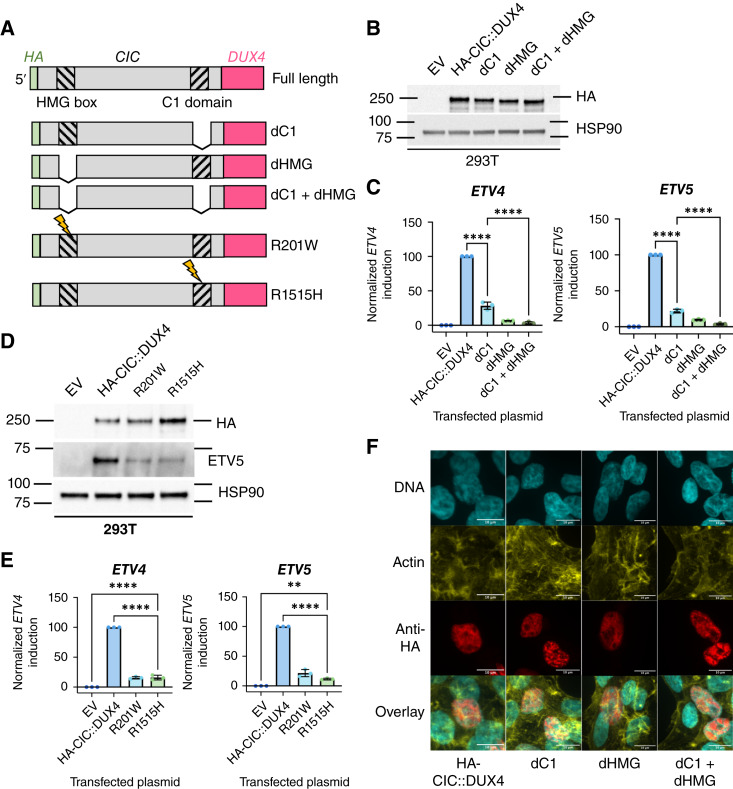
Deletion of the C1 domain reduces, but does not eliminate, CIC target gene induction without overtly affecting CIC::DUX4 protein expression or localization. **A,** Schematic of the engineered CIC::DUX4 deletion or point mutation variants. **B,** Immunoblot of 293T cells approximately 48 hours after transfection with EV or the indicated constructs; data representative of three independent experiments. **C,** Normalized RT-qPCR measurement of target gene (*ETV4* and *ETV5*) induction in 293T cells approximately 48 hours after transfection with EV or the indicated constructs. Each data point represents the mean of one of three independent experiments; error bars indicate SD (****, *P* < 0.0001) by one-way ANOVA and Šidák’s multiple comparisons test. **D,** Immunoblot of 293T cells approximately 48 hours after transfection with EV or the indicated constructs; data representative of three independent experiments. The HSP90 blot was aggregated with HA and ETV5 blots derived from identically loaded samples processed simultaneously; see Supplementary Dataset S3 for full Ponceau S loading controls and details. **E,** Normalized RT-qPCR measurement of target gene (*ETV4* and *ETV5*) induction in 293T cells approximately 48 hours after transfection with EV or the indicated constructs. Each data point represents the mean of one of three independent experiments; error bars indicate SD (****, *P* < 0.0001; **, *P* < 0.01) by one-way ANOVA and Šidák’s multiple comparisons test. **F,** Confocal immunofluorescence imaging of 293T cells approximately 48 hours after transfection with the labeled constructs. DNA visualized with DAPI and actin visualized with rhodamine phalloidin; 60× objective used for imaging. Scale bars, 10 μm. Representative cells chosen from one experiment.

In order to test the functional impact of the C1 domain on human CIC::DUX4 activity, we deleted the C1 domain, the HMG box, or both from an established HA-CIC::DUX4 expression construct (6) and expressed them in 293T cells (Fig. 2A). As the HMG box and the C1 domain have been shown to be required together for proper binding of CIC target DNA sites (bioRxiv 2022.03.28.485992; refs. 27, 62), the HMG/C1 co-deletion mutant functions as a DNA-binding-dead CIC::DUX4 negative control. All constructs were expressed at the expected protein level (Fig. 2B). Intriguingly, although deletion of the C1 domain significantly impaired the transcriptional induction of CIC target genes, the observed activation remained higher than the DNA-binding-dead control in the majority of tested targets (Fig. 2C; Supplementary Fig. S2A). To test if we could replicate these findings without large truncating deletions in the same functional domains, we engineered point mutations at residues R201 (HMG box, mutated to W) and R1515 (C1 domain, mutated to H) based on previously described hot spot mutations found in CIC-mutated gliomas (Fig. 2A; ref. 27). Indeed, we again observed that a point mutation in the C1 domain significantly reduced, but did not eliminate, the induction of CIC target genes (Fig. 2D and E; Supplementary Fig. S2B). We orthogonally validated these results by testing the activation of an *ETV5* promoter-luciferase construct carrying the consensus CIC binding sequence by the deletion mutants, finding again that the C1-deleted mutant showed incomplete abrogation of transcriptional activation (Supplementary Fig. S2C). Immunofluorescence microscopy of full-length or deletion-mutant HA-CIC::DUX4 transfected cells indicated that the deletions were not overtly impacting nuclear localization (Fig. 2F). Together, these results indicate that the C1 domain of CIC::DUX4 enhances the activation of CIC::DUX4 target genes in 293T cells.

### Stable NIH/3T3 and C2C12 clones validate differential induction of ETV5 by C1-intact or C1-deleted CIC::DUX4

**Figure 3:**
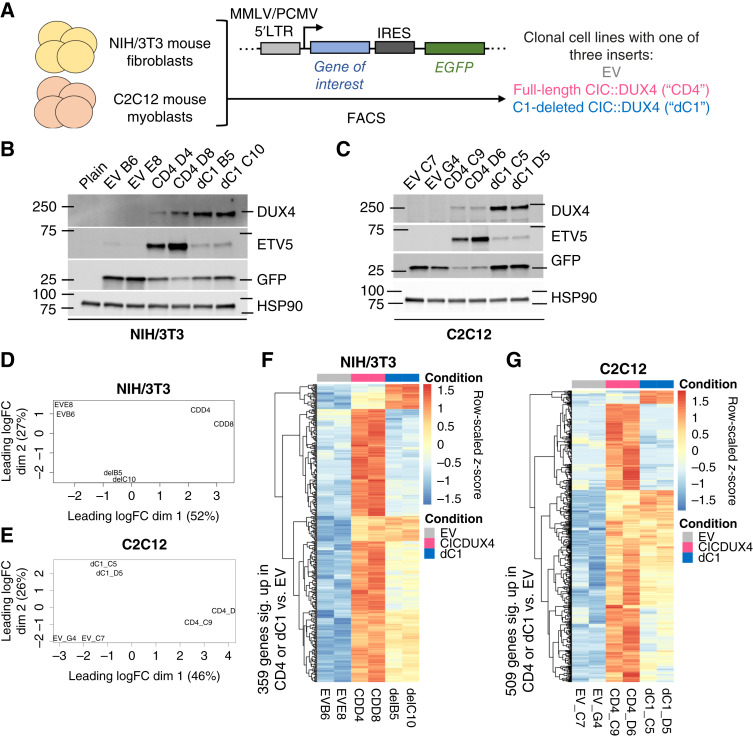
Stable expression of C1-deleted CIC::DUX4 in clonal NIH/3T3 and C2C12 cells drives a largely attenuated transcriptomic program defined by full-length CIC::DUX4 expression. **A,** Schematic describing how clonal populations of transduced NIH/3T3 and C2C12 cells were generated. **B,** Immunoblot of selected clonal NIH/3T3 cell lines in comparison with plain untransduced NIH/3T3. Clones were evaluated for transgene expression three independent times including the initial clone screening. The HSP90 and GFP blots were aggregated with DUX4 and ETV5 blots derived from identically loaded samples processed simultaneously; see Supplementary Dataset S3 for full Ponceau S loading controls and details. **C,** Immunoblot of selected clonal C2C12 cell lines. Clones were evaluated for transgene expression two independent times including the initial clone screening. The HSP90 blot was aggregated with DUX4, GFP, and ETV5 blots derived from identically loaded samples processed simultaneously; see Supplementary Dataset S3 for full Ponceau S loading controls and details. **D,** Multidimensional scaling plot of NIH/3T3 clone RNA-seq data after processing with edgeR. For clone name prefixes: EV, empty vector; CD, full-length CIC::DUX4; del, C1-deleted CIC::DUX4. **E,** Multidimensional scaling plot of C2C12 clone RNA-seq data after processing with edgeR. For clone name prefixes: EV, empty vector; CD4, full-length CIC::DUX4; dC1, C1-deleted CIC::DUX4. **F,** Row-scaled heatmap of 359 significantly upregulated (log_2_ fold change >2; *q* < 0.01) genes in either of the CD4 versus EV or dC1 versus EV comparisons across six NIH/3T3 clones. The same clone naming as in **D** applies. **G,** Row-scaled heatmap of 509 significantly upregulated (log_2_ fold change >2; *q* < 0.1) genes in either of the CD4 versus EV or dC1 versus EV comparisons across six C2C12 clones. The same clone naming as in **E** applies.

To broaden our findings beyond transient expression systems and 293T cells, we next aimed to stably express CIC::DUX4 or the C1-deleted mutant in cell lines more reflective of sarcoma biology. We chose to employ mouse NIH/3T3 fibroblasts and mouse C2C12 myoblasts, as both have previously been used to study CIC::DUX4 and other sarcomas (6, 24, 25) and represent the putative progenitor of soft tissue sarcomas (mesenchymal lineage—myoblast). We used a combination of retroviral transduction and fluorescence-activated cell sorting to generate clonal cell lines expressing “EV,” full-length CIC::DUX4 (“CD4”), or C1-deleted CIC::DUX4 (“dC1”) with an IRES-EGFP reporter (Fig. 3A). All but one evaluated NIH/3T3 clones expressed the proper transgenes (Supplementary Fig. S3A and S3B; Supplementary Dataset S3). Interestingly, although the C2C12 EV and dC1 clones all expressed the proper transgenes, only two of 12 tested C2C12 CD4 clones displayed measurable full-length CIC::DUX4 protein expression (Supplementary Fig. S3C and S3D; Supplementary Dataset S3). Across both NIH/3T3 and C2C12 clones, dC1 clones consistently expressed higher protein levels of mutant CIC::DUX4 than CD4 clones expressed full-length CIC::DUX4, which we speculate is in part due to toxicity and intolerance of high full-length fusion expression as previously described with other fusion oncoproteins (63). NIH/3T3 clones expressing either CIC::DUX4 version expressed less EGFP than the EV clones, suggesting that CIC::DUX4 transgenes were transcribed less than EV transgenes. In C2C12 cells, full-length CIC::DUX4 clones generally had lower EGFP expression than either of the other conditions, suggesting lower transcription of the full-length CIC::DUX4 transgene. Despite having lower fusion oncoprotein expression, CD4 clones expressing full-length CIC::DUX4 consistently demonstrated higher levels of ETV5 protein expression than dC1 clones expressing C1-deleted CIC::DUX4, corroborating our findings from 293T cells.

### Deletion of the C1 domain impairs, but does not fully eliminate, the transcriptional program controlled by CIC::DUX4 in NIH/3T3 cells

We selected two clones of each condition from the NIH/3T3 and C2C12 stable cell lines to continue with unbiased transcriptional analysis via bulk RNA-seq (Fig. 3B and C). Across both sets of cell lines, treating the two clones within each group as biological replicates and using multidimensional scaling indicated that the C1-deleted clones had a transcriptional profile in between those of either the EV or full-length CIC::DUX4 clones (Fig. 3D and E). A heatmap of all genes significantly upregulated (NIH/3T3: log_2_ fold change >2, *q* < 0.01; C2C12: log_2_ fold change >2, *q* < 0.1; Supplementary Datasets S4–S9) in either the CD4 or dC1 conditions versus EV revealed three major distinct blocks of genes regulated differently by the two versions of CIC::DUX4 (Fig. 3F and G). There was a minor subset of genes that were more strongly upregulated by C1-deleted CIC::DUX4 when compared with full-length CIC::DUX4 (“block 1,” top of heatmaps). However, many genes were strongly upregulated by full-length CIC::DUX4 but essentially not activated at all by C1-deleted CIC::DUX4 (“block 2,” in the upper middle of heatmaps). Separate from either of these two groups, the remaining genes comprised a third block of genes in which either activation did not depend on which CIC::DUX4 variant was expressed or expression was mildly attenuated in C1-deleted CIC::DUX4 clones compared with full-length CIC::DUX4 clones (“block 3,” bottom half of heatmaps). Gene set functional profiling of these three blocks of genes for both sets of clones indicated that block 1 was not consistently related to any terms, whereas blocks 2 and 3 were both associated with terms for anatomic and organismal development (Supplementary Datasets S10–S15).

Volcano plots of either CD4 or dC1 clones compared with EV clones revealed that the expression of essentially all genes significantly activated by full-length CIC::DUX4 was attenuated in the C1-deleted clones (Supplementary Fig. S4A). Focusing on six validated or high-confidence CIC::DUX4 target genes, we observed that although all six targets typically had lower expression in the dC1 clones compared with the CD4 clones, even this attenuated expression was often still significantly higher than that in the EV clones (Supplementary Fig. S4B and S4C). However, the magnitude of attenuation in the dC1 condition compared with the CD4 clones and the statistical significance of those differences varied with the particular target gene and the set of clones being analyzed (Supplementary Fig. S4B and S4C). Although the loss of the C1 domain generally impaired the CIC::DUX4-mediated transcriptional program in stable NIH/3T3 and C2C12 clones, it remained unclear whether this would translate to differences in functional phenotypes driven by these fusion oncoproteins.

### Deletion of the C1 domain yields intermediate CIC::DUX4-driven, transformation-related phenotypes

**Figure 4:**
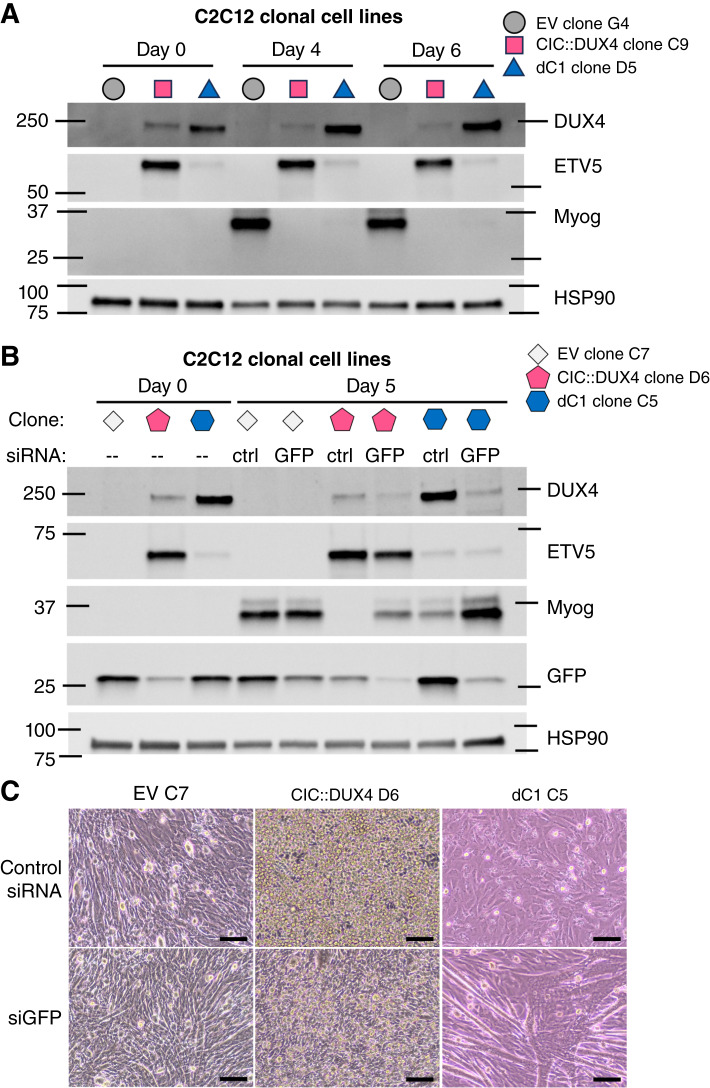
Expression of CIC::DUX4 with or without the C1 domain can block the differentiation of clonal C2C12 cells. **A,** Immunoblot of clonal C2C12 cells after differentiation for the indicated times, representative of two independent experiments. **B,** Immunoblot of clonal C2C12 cells after differentiation for the indicated times and following transfection with nontargeting (ctrl) or GFP-targeting siRNA, representative of two independent experiments. The HSP90 and GFP blots were aggregated with DUX5, ETV5, and Myog blots derived from identically loaded samples processed simultaneously; see Supplementary Dataset S3 for full Ponceau S loading controls and details. **C,** Microscopy images of C2C12 clones after 5 days of differentiation and treatment with the indicated siRNAs. Imaged using a 10× objective. Scale bar, 50 μm. Representative of two independent experiments.

Co-opting differentiation pathways is a known hallmark of some fusion oncoproteins, which to our knowledge has not been defined for CIC::DUX4. Thus, we first asked if CIC::DUX4 ± the C1 domain can interfere with C2C12 differentiation *in vitro*. C2C12 cells plated at confluency and serum starved differentiate into myotubes, in the process expressing key TF markers including myogenin (*Myog*; ref. 64). We subjected our C2C12 clonal cell lines to such an assay and used *Myog* expression as a proxy for reading out myotube differentiation. CIC::DUX4-expressing clones demonstrated reduced Myog protein expression relative to the EV clone, with the full-length CIC::DUX4 clone having no detectable protein expression and the C1-deleted clone having minimal expression at days 4 and 6 (Fig. 4A). To test that the observed change in Myog expression was a direct effect of CIC::DUX4 transgene expression, we next performed two separate rescue experiments in two sets of clones in which siRNAs were used to deplete transgene expression during differentiation. Specifically, we used either siRNA for GFP (targeting the whole transduced transcript, as in this system CIC::DUX4 and EGFP are polycistronic with an IRES) or for human *CIC* to deplete CIC::DUX4 expression during differentiation. siRNA targeting either GFP or human *CIC* had no detectable effect on Myog expression in the EV clone compared with control, whereas we again observed that control-treated CIC::DUX4-expressing clones yielded no (full-length CIC::DUX4) or reduced (C1-deleted CIC::DUX4) Myog expression (Fig. 4B; Supplementary Fig. S5A). Importantly, this was partially reversible with siGFP or siCIC treatment and accompanying mild knockdown of CIC::DUX4 protein at day 5, whereas siRNA treatment did not measurably change Myog expression in the EV clones (Fig. 4B; Supplementary Fig. S5A). For the siGFP rescue experiments, these results were also clear under microscopic inspection based on the relative appearances of elongating cell structures (Fig. 4C). For mechanistic insight into how CIC::DUX4 may be stifling the differentiation, we revisited our RNA-seq data of the C2C12 clones and noted that the full-length CIC::DUX4 clones had lower expression of three key myogenic transcription factors (*Myf5*, *Myod1*, and *Myog*) during steady-state growth, although these differences were not statistically significant (Supplementary Fig. S5B). Taken together, these results suggest that CIC::DUX4 expression does block myotube differentiation of C2C12 cells *in vitro* and that C1-deleted CIC::DUX4 is capable of driving a slightly attenuated but similar phenotype.

During the process of these differentiation experiments, we recorded images of differentiating cells at several time points with the intention of visualizing the elongation of cells into myotubes. While capturing these images, we unexpectedly observed phenotypic differences in cell growth related to CIC::DUX4 expression. The full-length CIC::DUX4 clones consistently continued proliferating throughout the whole experiment, seeming to grow vertically on top of their monolayer (control treated; Fig. 4C). This is in contrast to the EV and dC1 clones, which either remained a monolayer or were subconfluent while differentiating (control treated; Fig. 4C). This was best visualized as a time course during the initial differentiation experiments (Supplementary Fig. S6). Indeed, the observations that both CD4 clones demonstrated this growth pattern and that siGFP treatment seemed to reduce this overgrowth in CD4 clone D6 (Fig. 4C) suggest that this is a phenotype driven by the expression of full-length CIC::DUX4.

As an additional means of exploring growth-related outcomes, we leveraged our NIH/3T3 clones to test 3D growth/cell–cell adhesion phenotypes in a hanging drop assay. Although the EV clones were capable of forming small tightly packed spheroids with few isolated cells, the full-length CIC::DUX4 clones displayed atypical growth patterns with many free-floating dissociated cells not associated with larger assemblies (Supplementary Fig. S7). The C1-deleted clones demonstrated an intermediate phenotype, with one clone (B5) forming fairly EV-like spheroids whereas the other (C10) formed small spheroids that clumped up in chains or larger structures (Supplementary Fig. S7).

Finally, as our lab has previously studied the role of CIC::DUX4 in regulating cell proliferation (24) and given that changes in proliferation rates may impact other assays, we evaluated the growth rates of all clones. On average, we observed relatively similar growth rates across all NIH/3T3 clones, whereas in C2C12 cells the full-length CIC::DUX4 clones on average grew faster than the dC1 clones, which grew similarly to but perhaps slightly quicker than the EV clones (Supplementary Fig. S8A–S8F).

Taken together, these results suggest that CIC::DUX4 harboring deletion of the C1 domain can still yield phenotypes intermediate to those conferred by full-length CIC::DUX4, including some transformation-related phenomena most notably including differentiation blocking.

### Expression of C1-deleted CIC::DUX4 in C2C12 clones is not sufficient to drive tumorigenesis in nude mice

**Figure 5:**
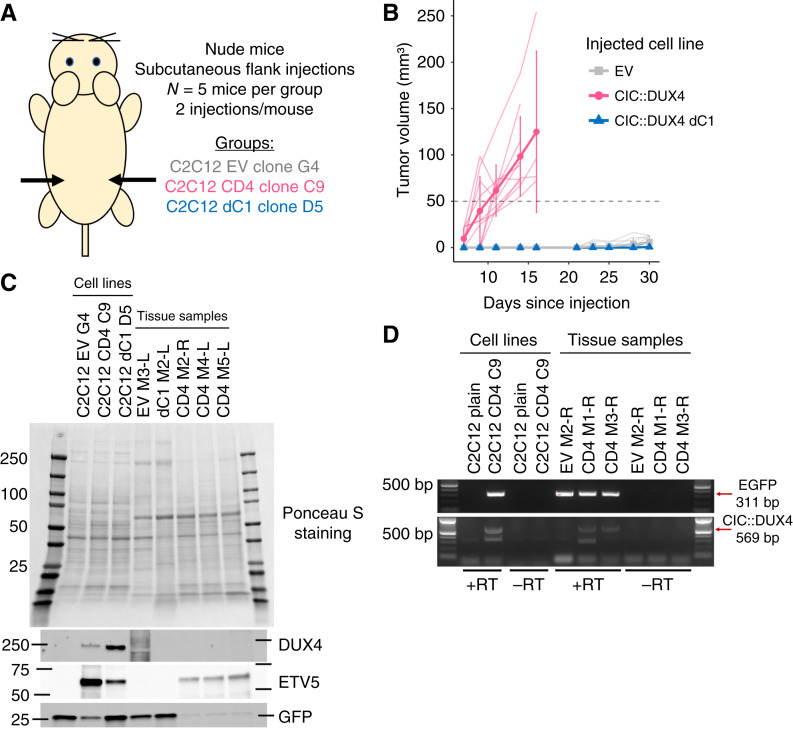
C1-deleted CIC::DUX4-expressing C2C12 cells do not initiate tumor formation in nude mice. **A,** Schematic of clonal C2C12 subcutaneous implantation experimental design. **B,** Plot of tumor volumes versus time; thin transparent lines indicate individual injection sites, whereas thick opaque lines and data points represent averages. Error bars represent SD. Note that three full-length CIC::DUX4-injected mice were sacrificed at day 14, and the remaining two mice in that group were sacrificed at day 16. **C,** Ponceau S staining and immunoblots of clonal C2C12 cell lines or explanted tumor tissue. Tumor tissue is labeled by injected cell line, mouse number, and flank side. Protein analysis was performed once because of inability to detect CIC::DUX4 expression in tissue. **D,** Agarose gel electrophoresis of PCR products from cDNA derived from C2C12 cell lines or explanted tumor tissue. Arrows indicate expected sizes of positive PCR products for the indicated transcripts. The smaller band at approximately 300 to 350 bp in the CIC::DUX4 reaction is nonspecific. +/−RT refers to inclusion/exclusion of reverse transcriptase in the cDNA generation step. Red pixels within bands indicate saturated signal. The same cDNA from tissue samples was tested two separate times with the same results.

As we consistently observed intermediate activity of the C1-deleted CIC::DUX4 compared with full-length CIC::DUX4 across several *in vitro* assays, we next aimed to test the impact on tumor growth *in vivo*. We subcutaneously implanted three of our similarly fast-growing C2C12 clones into the flanks of immunodeficient (nude) mice (Fig. 5A). C2C12 myoblasts are representative of a mesenchymal stem cell progenitor, and they only generate lesions over fairly long time frames when implanted in mice (65–67). Consistent with these findings, only half of the injection sites (5/10, 50%) of EV-injected mice developed small (less than 14 mm^3^) slow-growing lesions over the course of the 30-day experiment (Fig. 5B; Supplementary Fig. S9A). In contrast, full-length CIC::DUX4-injected mice rapidly developed tumors (10/10, 100%) of at least 50 mm^3^ by day 14 postinjection (Fig. 5B; Supplementary Fig. S9A). Three CD4-injected mice had to be sacrificed at day 14 because of ulcerating tumors, whereas the remaining two CD4-injected mice were sacrificed at day 16 to avoid ulceration from their established tumors. Unexpectedly, mice injected with C1-deleted CIC::DUX4-expressing cells did not develop measurable tumors at study termination, with only one injection site having a small lesion of 6 mm^3^ (1/10; 10%; Fig. 5B; Supplementary Fig. S9A). Western blot analysis of explanted tissue could not reliably detect CIC::DUX4 protein expression in any tumor sample but did reveal increased ETV5 levels in full-length CIC::DUX4 tumors compared with EV or dC1 tissue (Fig. 5C). We were able to confirm CIC::DUX4 transcript expression at the RNA level for full-length CIC::DUX4 tumors (Fig. 5D) but did not have remaining tissue to evaluate dC1 lesions. These results suggest that in this particular model, C1-deleted CIC::DUX4 was not sufficient to drive tumorigenesis.

## Discussion

Fusion oncogenes, including TF fusions, are often defined at the gene level (i.e., *EWS*::*FLI1*, not *EWSR1* exon 7::*FLI* exon 5). This is certainly understandable given the vast number of fusion oncogenes driving human disease and the requirement for rapid and precise clinical and diagnostic interpretation. However, specific breakpoints where partner genes are fused in the context of these rearrangements are not always identical (13–15). We therefore argue that there is value in understanding where those sites occur and how the inclusion or exclusion of key regulatory domains from the partner genes affects fusion oncoprotein function.

Our analysis of CIC::DUX4 breakpoints described in the literature reveals that in *bona fide* CIC::DUX4 fusion transcripts, *DUX4* breakpoints occur almost anywhere after the HOX1 domain and prior to the C-terminal region responsible for the transactivation and interaction with p300. This distribution of *DUX4* breakpoint locations is consistent with the current model that DUX4 contributes its p300 recruitment and transactivation capacities to the CIC::DUX4 fusion oncoprotein but not its DNA-binding specificity. In contrast to the variability observed for *DUX4* breakpoints, *CIC* breakpoints occur almost exclusively at the very 3′ end of the coding sequence, retaining essentially all of the *CIC* transcripts (4,500+ nucleotides) in the fusion. Although this could be due to factors like sequence-specific properties which make those regions more likely to form translocations, a more compelling, evolution-based rationale is that breakpoints at the 3′ end of *CIC* preserve functional domain(s) important for the activity of the fusion and thus tumorigenesis. In support of this hypothesis, the major exception to C1 domain–retaining breakpoints in CIC::DUX4 fusions is the class of CIC::UTR fusions which are not genuine coding CIC::DUX4 fusions. In these noncanonical fusions, rather than retaining the C1 domain and forming a transactivating fusion with *DUX4*, the breakpoint seems likely to disrupt the C1 domain (and potentially other C-terminal regions) and function as a DUX4-independent, dominant-negative *CIC* mutant as has been hypothesized by others (56). We observed statistically significant but minor activity of such a CIC::UTR fusion when expressed in 293T cells, but further study is warranted, particularly in the context of a potential requirement for haplo-insufficiency and to determine if even a mild upregulation of target genes by a potentially dominant-negative CIC::UTR is sufficient for transformation.

We observed through multiple separate cell models that deletion of the C1 domain leads to an attenuated activation of CIC::DUX4 target genes but does not entirely abrogate the CIC::DUX4 transcriptional program. One limitation of interpreting this finding is that not all target genes responded to the same degree. One possible explanation for this depends on recent findings by our group and others that CIC and CIC::DUX4 may additionally bind noncanonical sites on DNA in addition to the preferred octamer (bioRxiv 2023.10.11.561932; refs. 23, 62, 68). If the C1 domain is playing a role in binding DNA in CIC::DUX4, which seems highly likely, then its loss may differentially impact binding to target genes with variable binding motifs in their respective regulatory elements. This in turn may differentially influence chromatin remodeling near such target genes.

A second difficulty in determining whether reduced but significant activation of target genes by C1-deleted CIC::DUX4 is functionally meaningful lies in the field’s lack of understanding of how much CIC::DUX4 expression is ideal for tumorigenesis. This question goes hand in hand with the issue of the “Goldilocks principle” for fusion oncoproteins, as was recently described by Seong and colleagues (63) for EWS::FLI. Our data from CIC::DUX4 stable NIH/3T3 and C2C12 clones seem to converge on the notion that too much full-length CIC::DUX4 is toxic to cells, as the full-length CIC::DUX4 clones expressed much less fusion at the protein level than the weaker C1-deleted CIC::DUX4 clones, despite being driven by an otherwise identical retroviral construct. Indeed, most C2C12 full-length CIC::DUX4 clones did not show a detectable fusion protein, suggesting that there was selective pressure to silence the transgene. Thus, an improved understanding of endogenous CIC::DUX4 dosage and how it impacts tumor cell survival and death can enhance our understanding of fusion oncoprotein stability and expression.

While exploring phenotypic consequences of deleting the C1 domain, we showed for the first time that CIC::DUX4 can block the differentiation of C2C12 cells. This is an exciting finding given the ability of other fusion oncoproteins to cause disease by hijacking differentiation, but we remain unsure if this phenotype is pathogenically relevant. For one, although C1-deleted CIC::DUX4 impaired the expression of Myog, the same transduced cells were unable to initiate tumors *in vivo*. Moreover, although a primitive mesenchymal progenitor is hypothesized to be the cell of origin for CIC::DUX4 tumors, it is plausible that C2C12 mouse myoblasts are not an accurate cell model to study CIC::DUX4-mediated transformation. Consequently, defining the correct cellular context to study CIC::DUX4 initiation is also an area of importance.

This study has several limitations. First, we did not employ patient-derived cell lines endogenously harboring CIC::DUX4 due to our group’s prior experience attempting to knock out CIC::DUX4 from NCC_CDS1_X1_C1 cells (30) with CRISPR-Cas9, in which cell viability was extremely limited upon selection (11), likely because of dependence on the fusion. Consequently, we would expect that mutating the endogenous C1 domain and thus impairing fusion function would lead to loss of cell viability. Although the cell lines used here do not directly mimic patient biology, they do reflect proper transcriptional responses to the expression of CIC::DUX4 and are easily engineerable, making them useful for mechanistic studies. Second, our *in vivo* model may not completely reflect human biology because a small number of breakpoints identified in patients are *bona fide* CIC::DUX4 fusions that do not retain the C1 domain. Although the lack of tumorigenesis for C1-deleted CIC::DUX4-expressing C2C12 cells is consistent with their absence of beyond-monolayer growth compared with that observed for full-length CIC::DUX4 clones in the differentiation assay, we cannot rule out that C1-deleted CIC::DUX4 may be capable of tumorigenesis in the right cellular context. Third, our breakpoint database is inherently biased by the approaches used to identify breakpoints in patients. Because some CIC-rearranged genes are identified with fixed PCR primers that target only certain parts of the partner genes, this may lead to the unintentional exclusion of breakpoints missed by such approaches.

In summary, we show that the *CIC* C1 domain is necessary for the maximal activity of the CIC::DUX4 fusion oncoprotein. This work builds upon pioneering data from WT CIC and experiments using a fly CIC::DUX4 chimera in *Drosophila* (27) to extend these findings to mammalian and disease-relevant settings. Our results demonstrate an example of how paying attention to fusion breakpoints may be informative of which partner gene functional domains are useful to the nascent oncoprotein. Finally, although we do not definitively show that the C1 domain is an essential vulnerability of CIC::DUX4, targeting this key functional domain may be worth further study. Beyond CIC-rearranged tumors, we encourage clinicians and researchers alike to be mindful of reporting fusion oncogene breakpoints in a consistent and accessible manner so that we may use the natural evolution of fusions to tell us about their important functional domains.

## Supplementary Material

Supplementary Dataset S1 Full CIC-fusion breakpoint database.

Supplementary Dataset S2 Cloning PCR primer sequences.

Supplementary Dataset S3 Uncropped blots/gels and full Ponceau S loading controls.

Supplementary Dataset S4 edgeR DE output for NIH3T3 CD4 vs EV, larger log_2_FC values = higher in CD4.

Supplementary Dataset S5 edgeR DE output for NIH3T3 dC1 vs EV, larger log2FC values = higher in dC1.

Supplementary Dataset S6 edgeR DE output for NIH3T3 dC1 vs CD4, larger log2FC values = higher in dC1.

Supplementary Dataset S7 edgeR DE output for C2C12 CD4 vs EV, larger log2FC values = higher in CD4.

Supplementary Dataset S8 edgeR DE output for C2C12 dC1 vs EV, larger log2FC values = higher in dC1.

Supplementary Dataset S9 edgeR DE output for C2C12 dC1 vs CD4, larger log2FC values = higher in dC1.

Supplementary Dataset S10 gProfiler2 output for NIH/3T3 block 1 genes.

Supplementary Dataset S11 gProfiler2 output for NIH/3T3 block 2 genes.

Supplementary Dataset S12 gProfiler2 output for NIH/3T3 block 3 genes.

Supplementary Dataset S13 gProfiler2 output for C2C12 block 1 genes.

Supplementary Dataset S14 gProfiler2 output for C2C12 block 2 genes.

Supplementary Dataset S15 gProfiler2 output for C2C12 block 3 genes.

Supplementary Figure S1 CIC breakpoints are mildly variable across different 3’ partner genes, and piloting of a CIC::UTR “fusion” model.

Supplementary Figure S2 Deletion or point mutation of the C1 domain result in attenuated target gene activation and ETV5-promoter engagement by CIC::DUX4.

Supplementary Figure S3 Screening and validation of transduced NIH/3T3 and C2C12 clonal cell lines.

Supplementary Figure S4 Deletion of the C1 domain attenuates activation of CIC::DUX4-induced genes, but varies in magnitude on some known target genes.

Supplementary Figure S5 siCIC treatment can rescue C2C12 clone CIC::DUX4-related differentiation defects, and analysis of myogenic differentiation-related gene expression in C2C12 clones.

Supplementary Figure S6 C1-intact CIC::DUX4 expression alters the growth pattern of clonal C2C12 cells.

Supplementary Figure S7 Full-length or C1-deleted CIC::DUX4 expression alters 3D growth in NIH/3T3 clones.

Supplementary Figure S8 Growth dynamics of clonal NIH/3T3 and C2C12 cell lines transduced with empty vector, full length CIC::DUX4, or C1-deleted CIC::DUX4.

Supplementary Figure S9 Only full-length CIC::DUX4 expressing C2C12 cells are capable of forming overt tumors in a nude mouse subcutaneous injection model.
