# Supplementary material for: The Capicua C1 Domain Is Required for Full Activity of the CIC::DUX4 Fusion Oncoprotein
Source: Cancer Res Commun. 2024 Dec 9;4(12):3099–113. doi: 10.1158/2767-9764.CRC-24-0348 (PMC11626509; doi:10.1158/2767-9764.CRC-24-0348)
Supplement: Supplementary Dataset S3 — Uncropped blots/gels and full Ponceau S loading controls. [file crc-24-0348_supplementary_dataset_s3_suppsd3.pdf]

Figure 2 Panel B

Uncropped Chemiluminescence Image

HSP90

HA

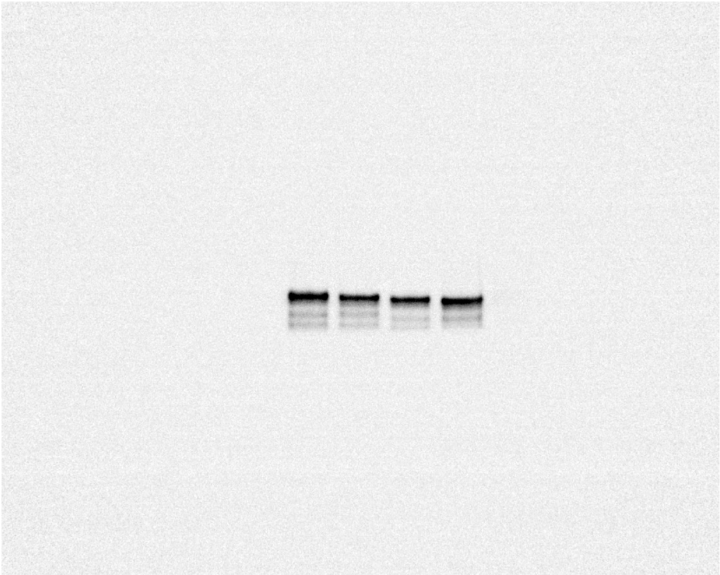

Uncropped Composite (with Colorimetric, for ladder)

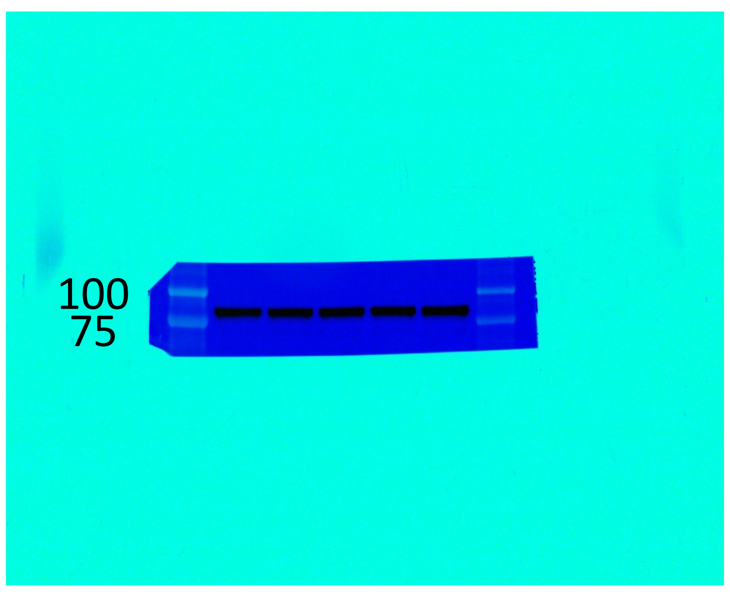

Note: chemi. brightness was adjusted in Image Lab, hence darker bands in the original composite

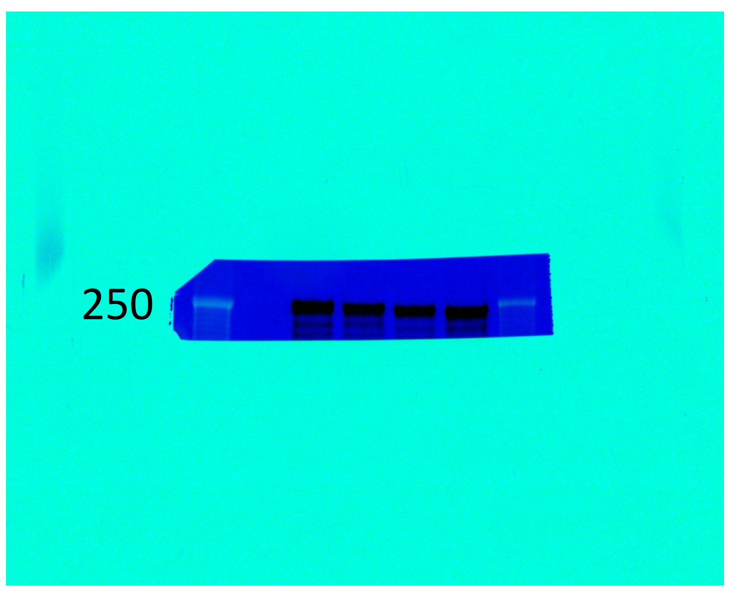

Note: chemi. brightness was adjusted in Image Lab, hence darker bands in the original composite

Ponceau S Staining

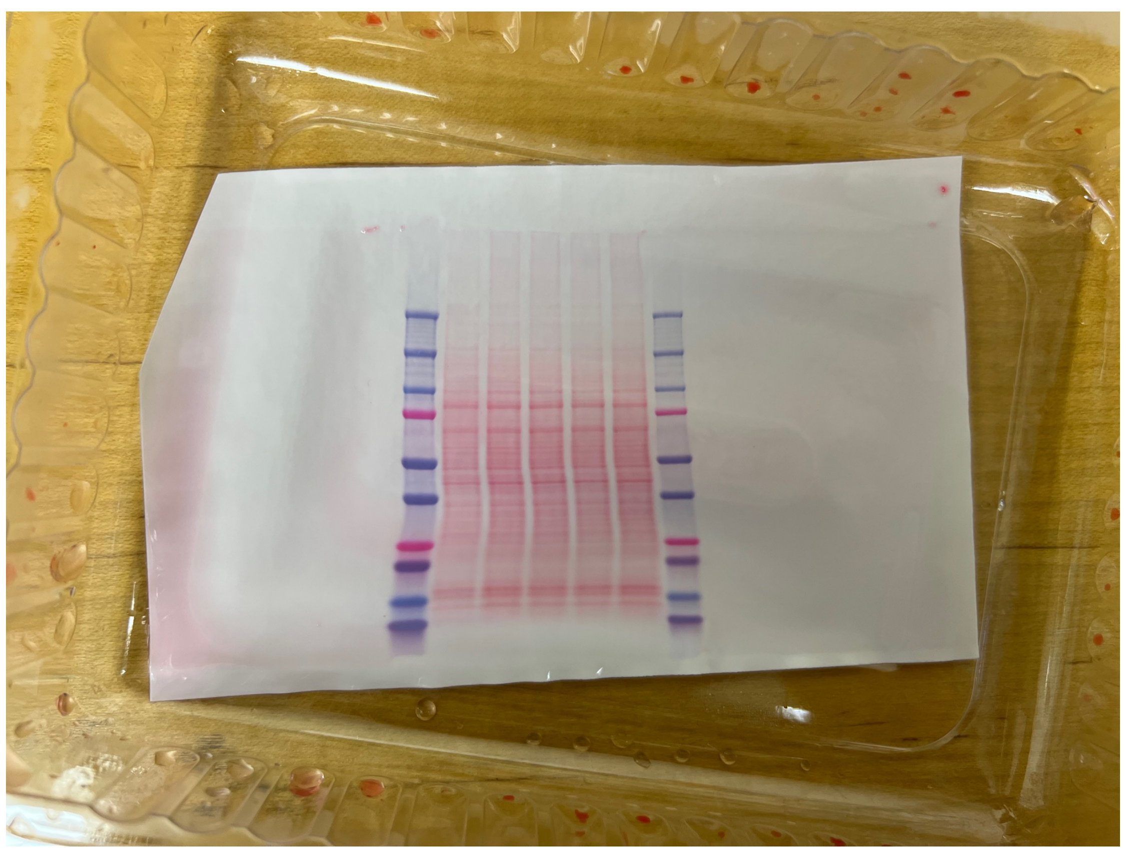

Note: imaged on iPhone

Sample order, left to right:
7 uL ladder
EV
HA-CIC::DUX4
dC1
dHMG
dC1 + dHMG
3 uL ladder

Figure 2 Panel D

Uncropped Chemiluminescence Image

HSP90

HA

ETV5

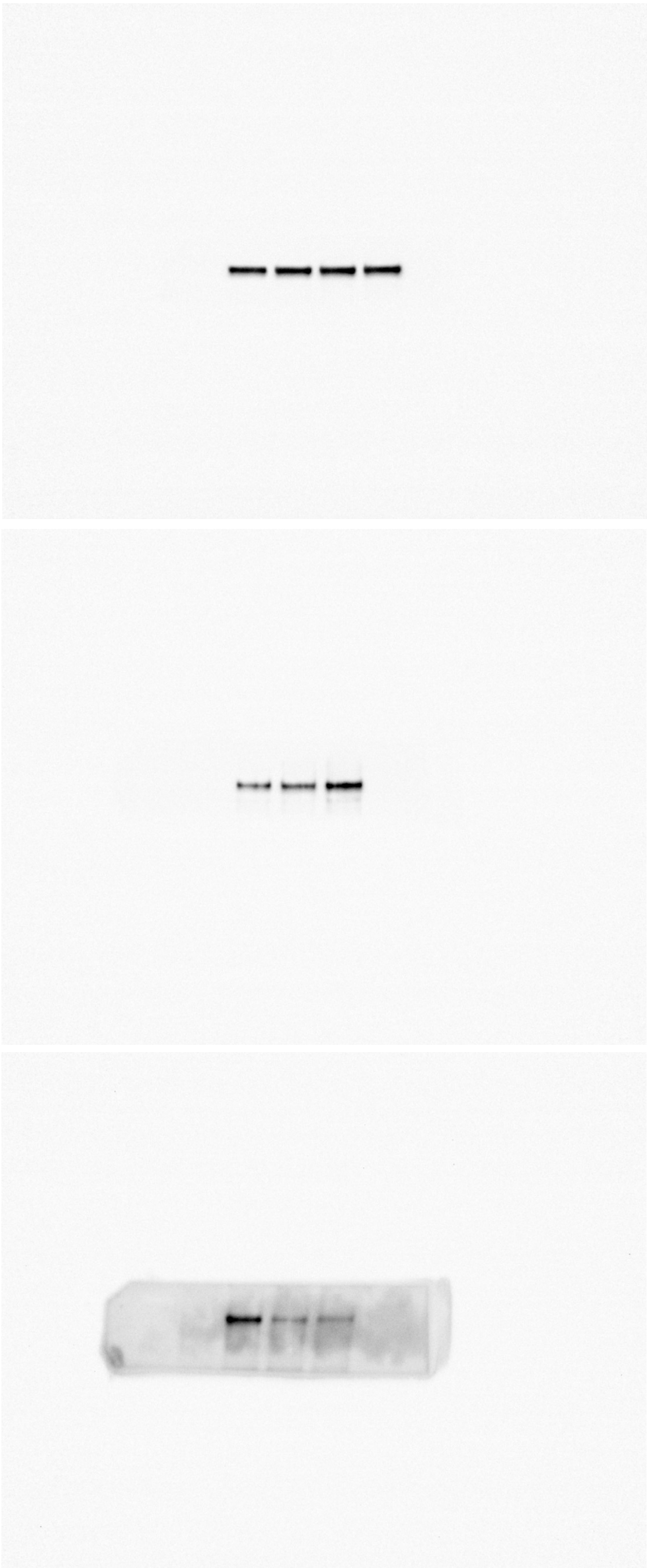

Uncropped Composite (with Colorimetric, for ladder)

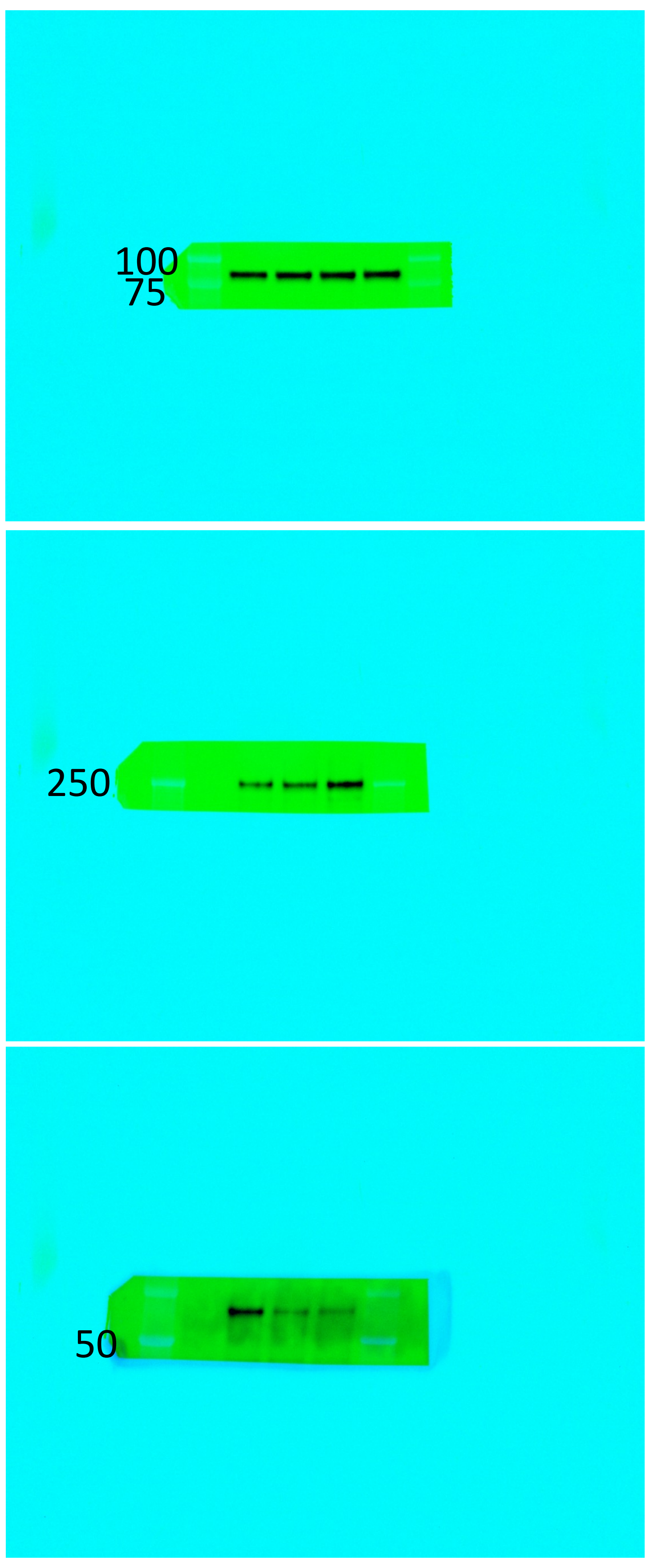

Ponceau S Staining

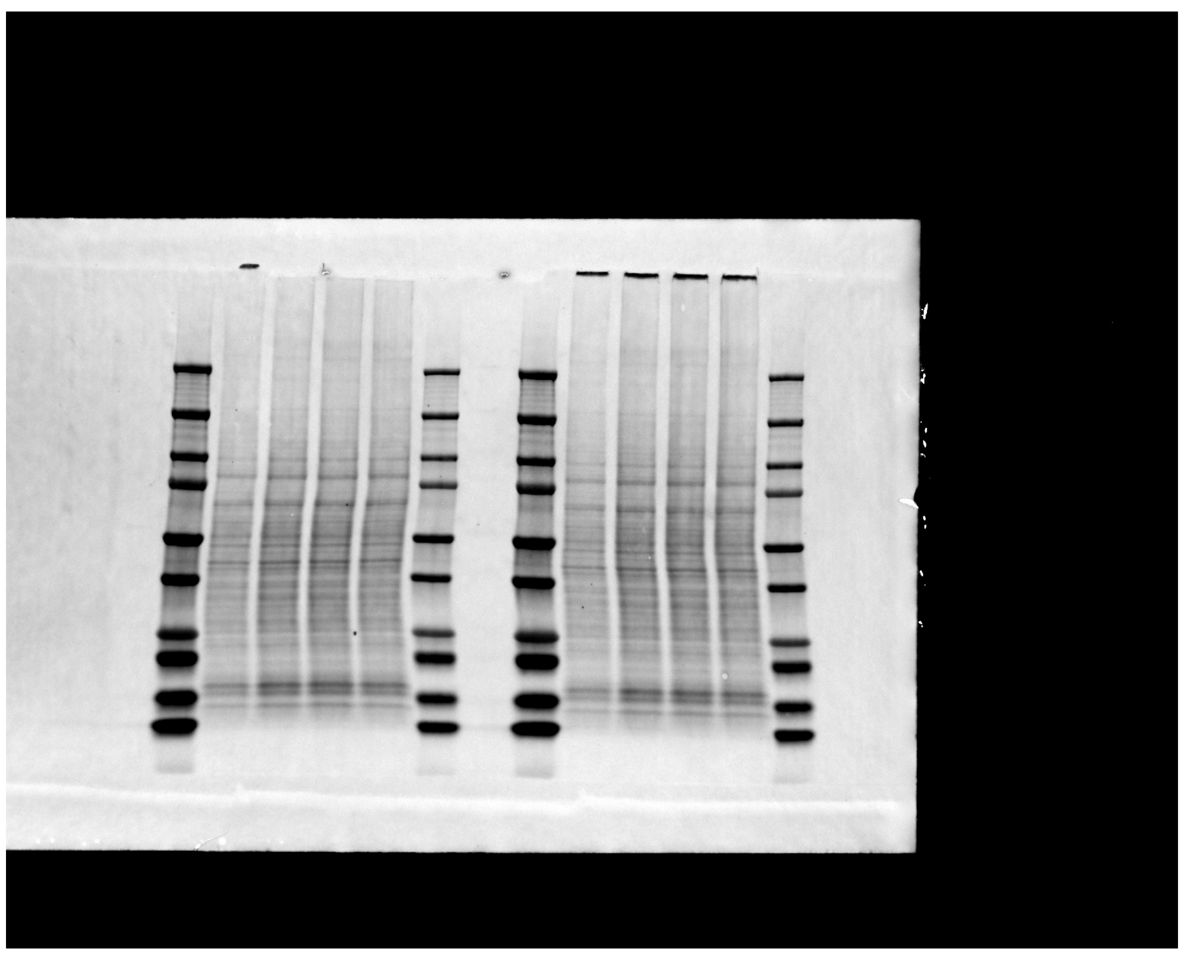

Sample order, left to right:
7 uL ladder
EV
HA-CIC::DUX4
R201W
R1515H
3 uL ladder

Note: HA tag and ETV5 cut from the same set of samples, HSP90 cut from the other.

Figure 3 Panel B

Uncropped Chemiluminescence Image

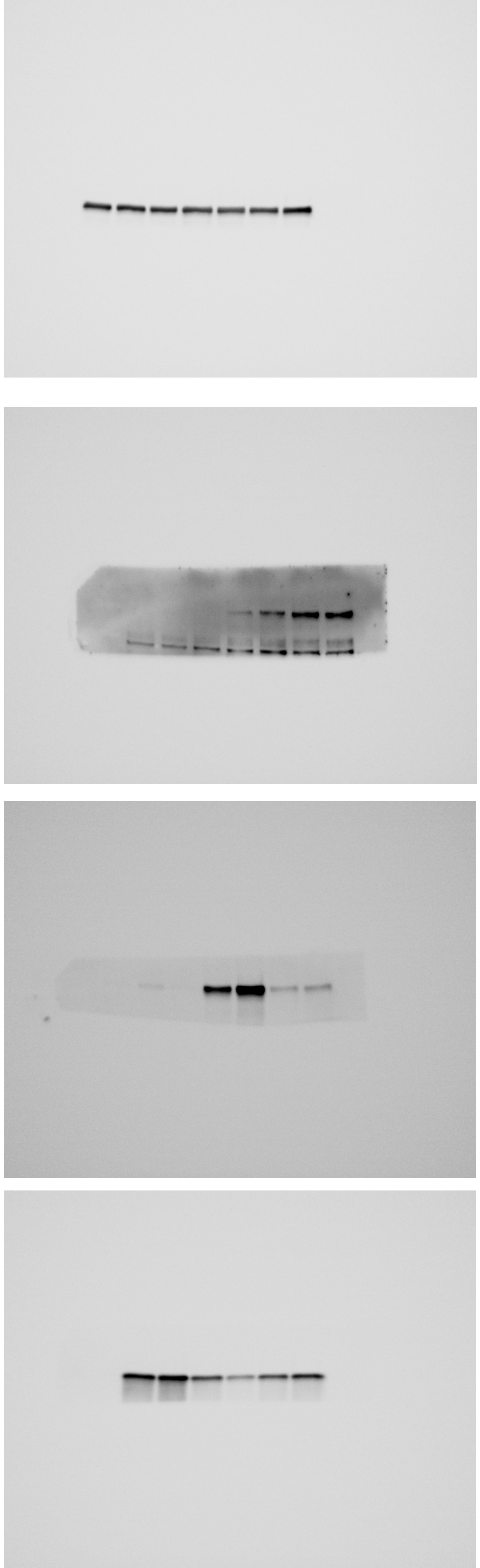

Uncropped Composite (with Colorimetric, for ladder)

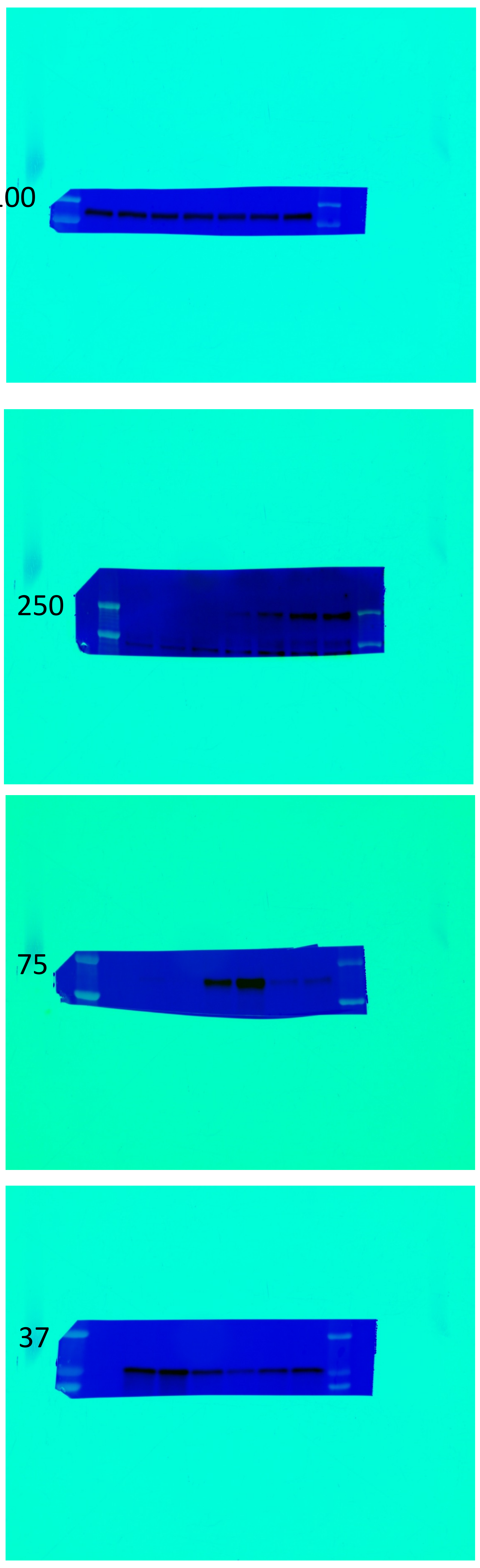

Ponceau S Staining

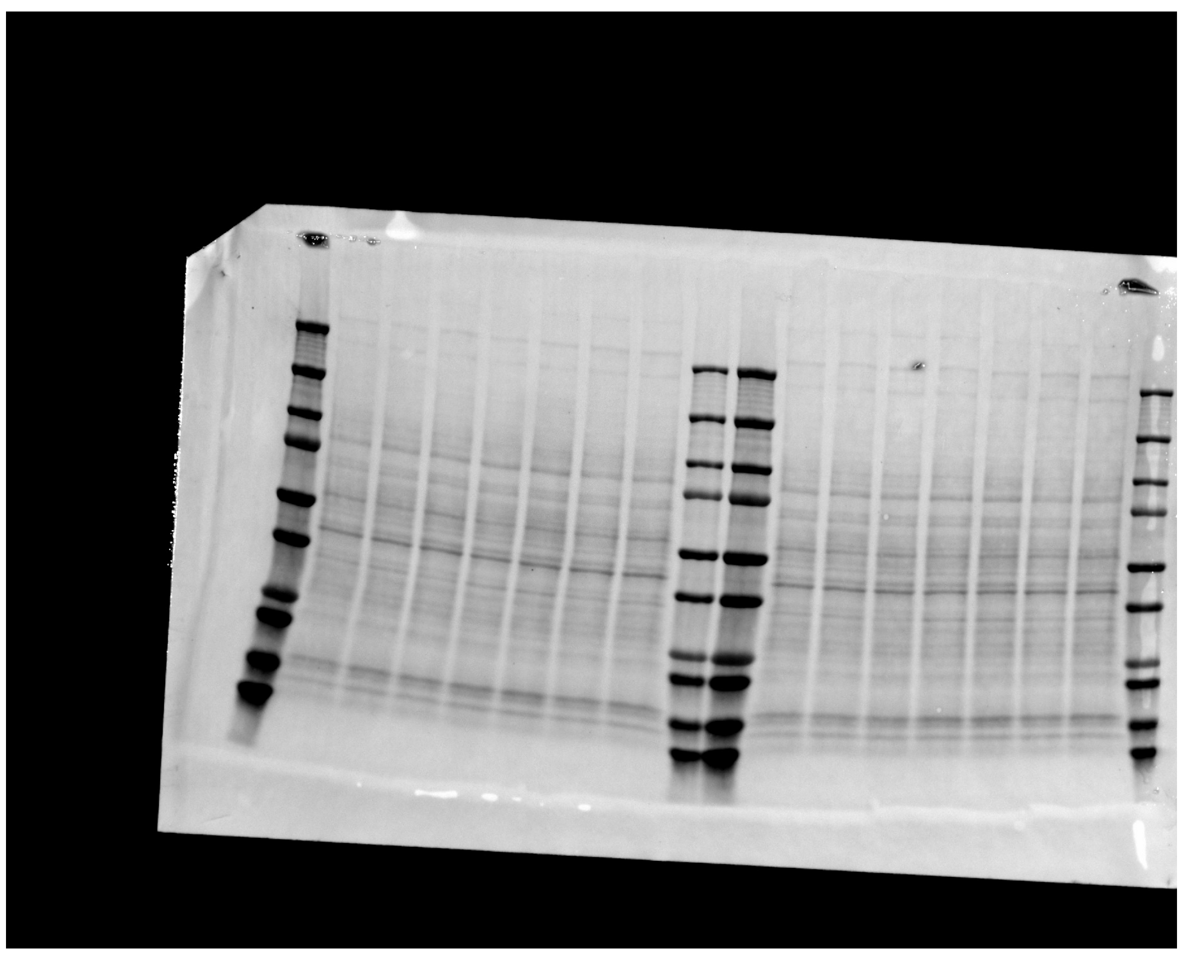

Sample order, left to right:
7 uL ladder
Plain
EV B6
EV E8
CICDUX4 D4
CICDUX4 D8
dC1 B5
dC1 C10
3 uL ladder

Note: DUX4 and ETV5 cut from the same set of samples (left), HSP90 and GFP cut from the other set of samples (right)

Figure 3 Panel C

Uncropped Chemiluminescence Image

HSP90

DUX4

ETV5

GFP

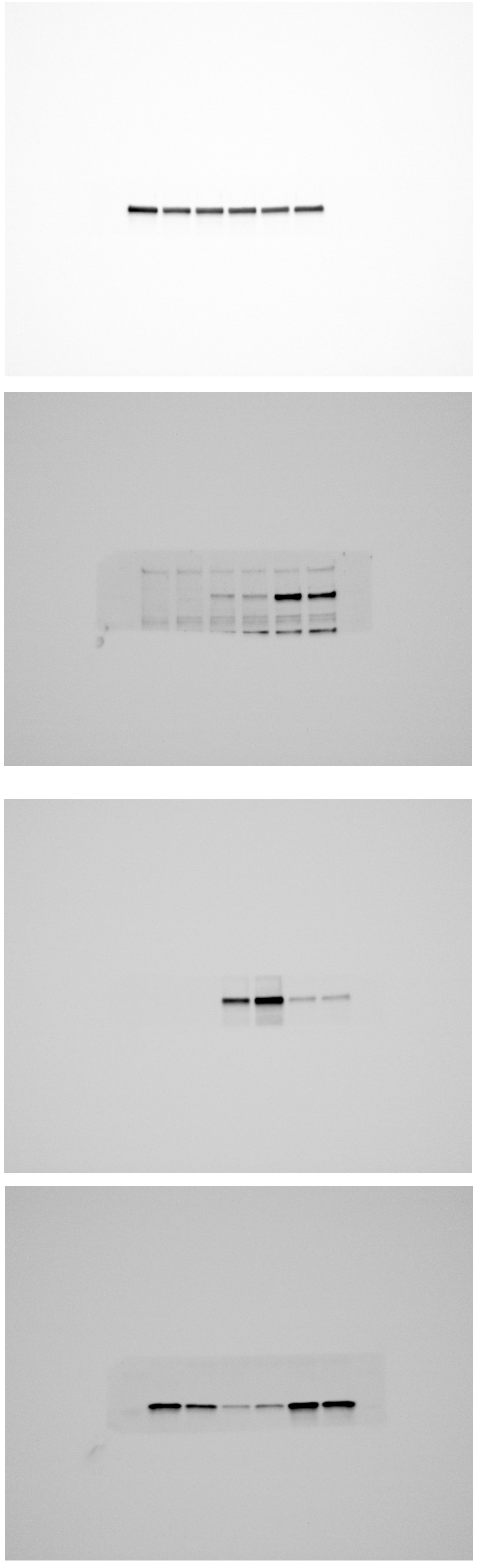

Uncropped Composite (with Colorimetric, for ladder)

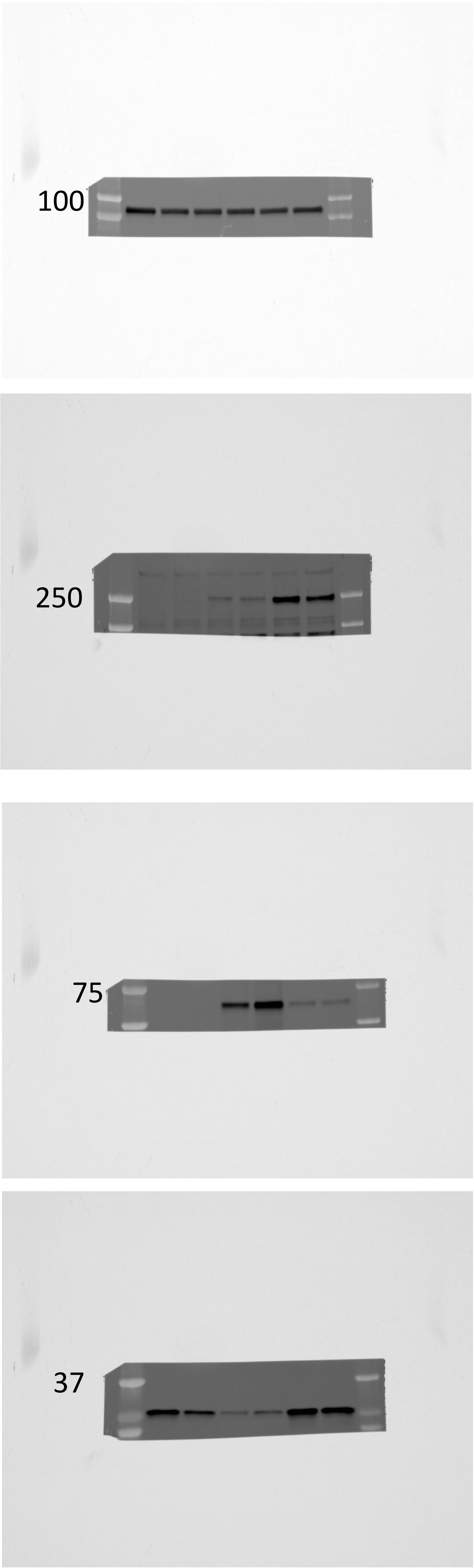

Ponceau S Staining

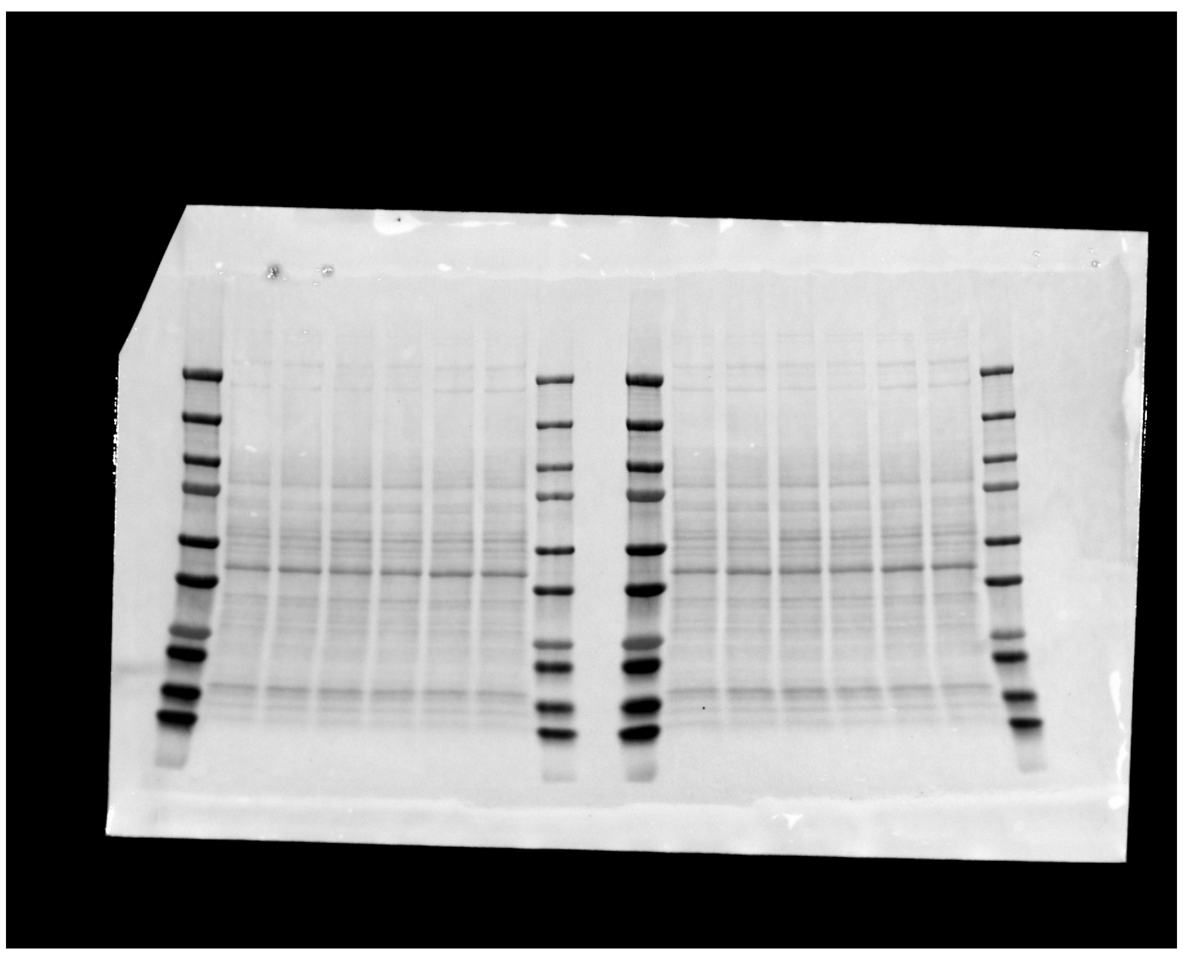

Note: HSP90 cut from the left set of samples, DUX4, ETV5, and GFP cut from the right set of samples.

Sample order, left to right:
7 uL precision plus ladder
C2C12 EV C7
C2C12 EV G4
C2C12 CD4 C9
C2C12 CD4 D6
C2C12 dC1 C5
C2C12 dC1 D5
3 uL precision plus ladder

Figure 4 Panel A

Uncropped Chemiluminescence Image

HSP90

DUX4

ETV5

Myogenin

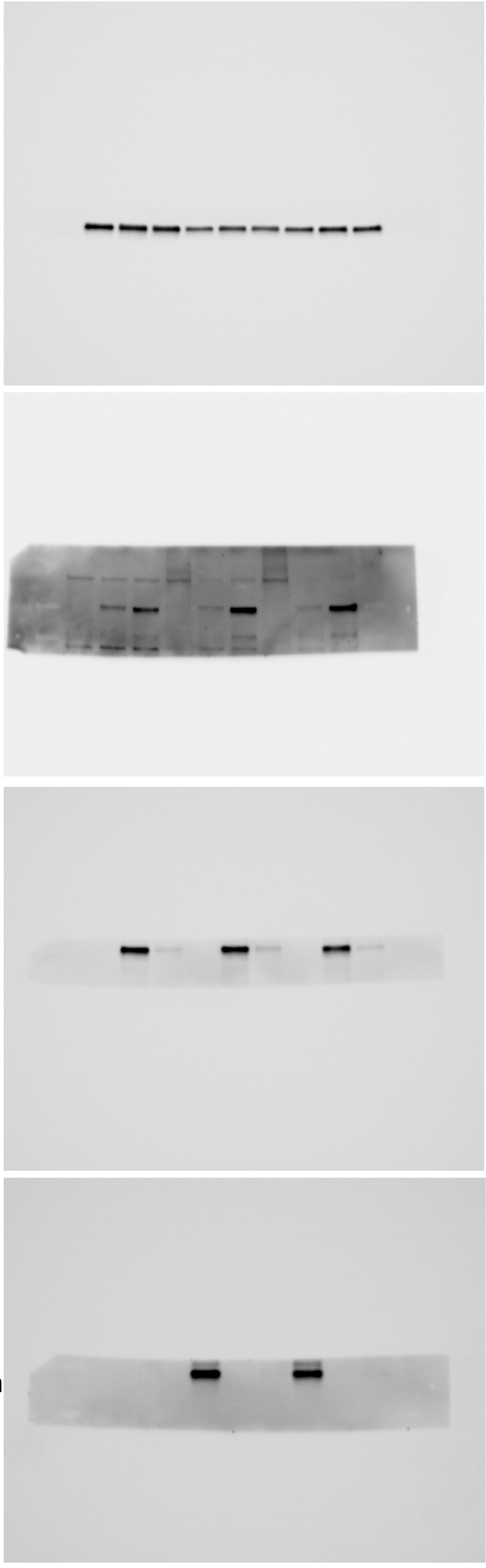

Uncropped Composite (with Colorimetric, for ladder)

100

250

50

37

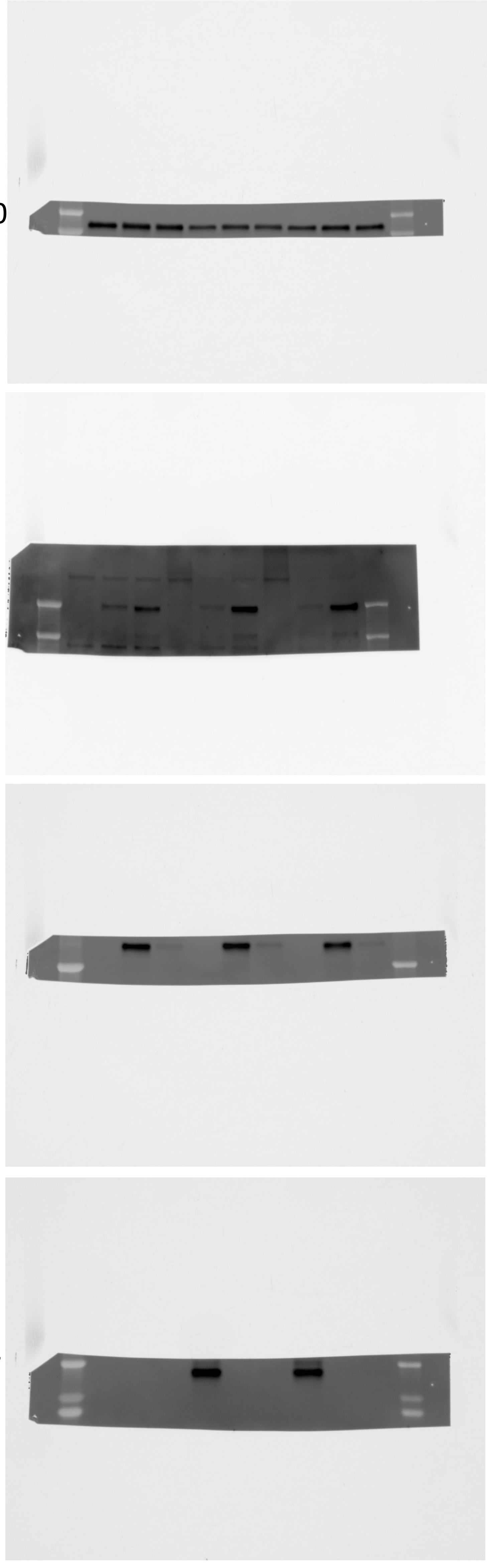

Ponceau S Staining

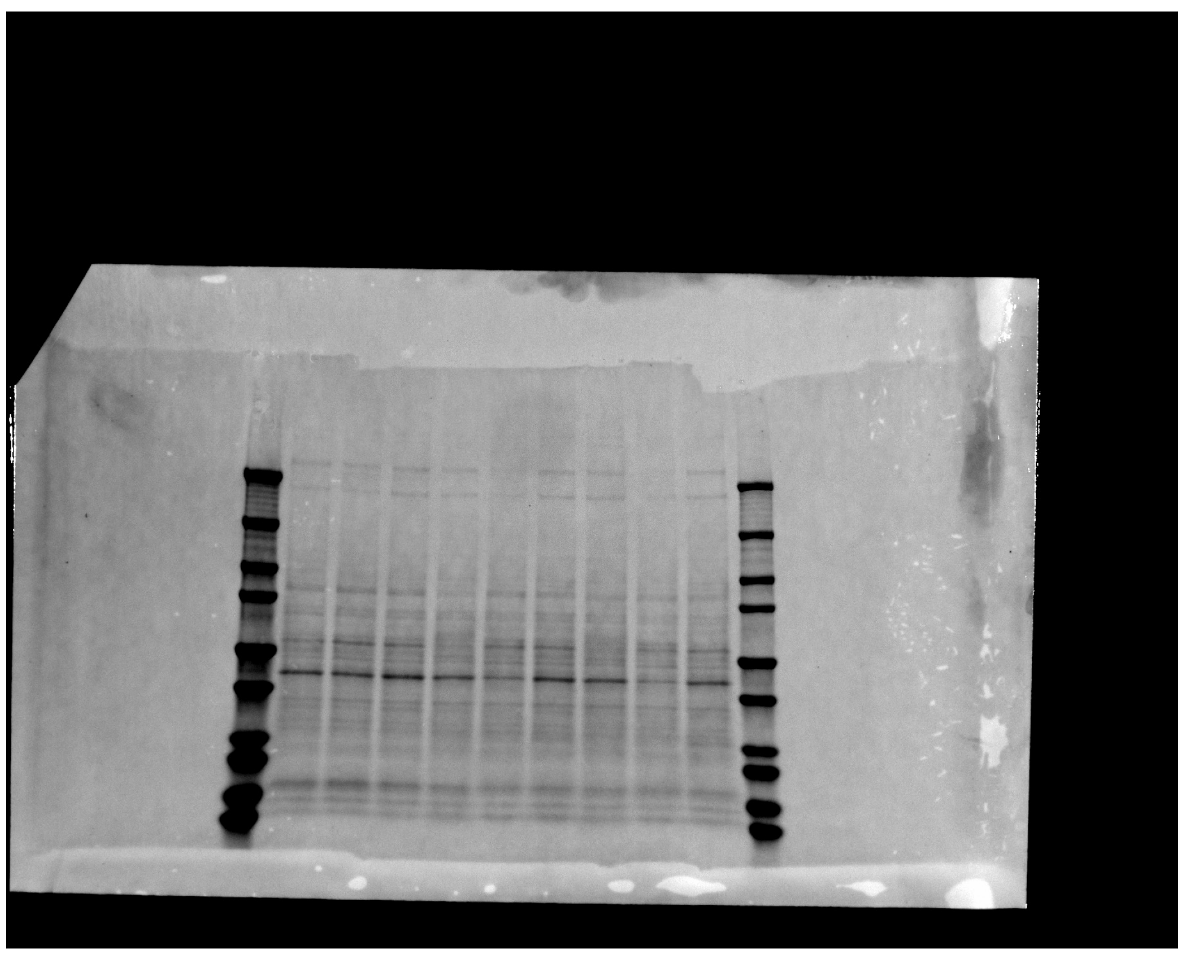

Sample order, left to right:
7 uL ladder
EV G4 d0
CICDUX4 C9 d0
dC1 D5 d0
EV G4 d4
CICDUX4 C9 d4
dC1 D5 d4
EV G4 d6
CICDUX4 C9 d6
dC1 D5 d6
3 uL ladder

Figure 4 Panel B

Uncropped Chemiluminescence Image

Uncropped Composite (with Colorimetric, for ladder)

HSP90

DUX4

ETV5

Myogenin

GFP

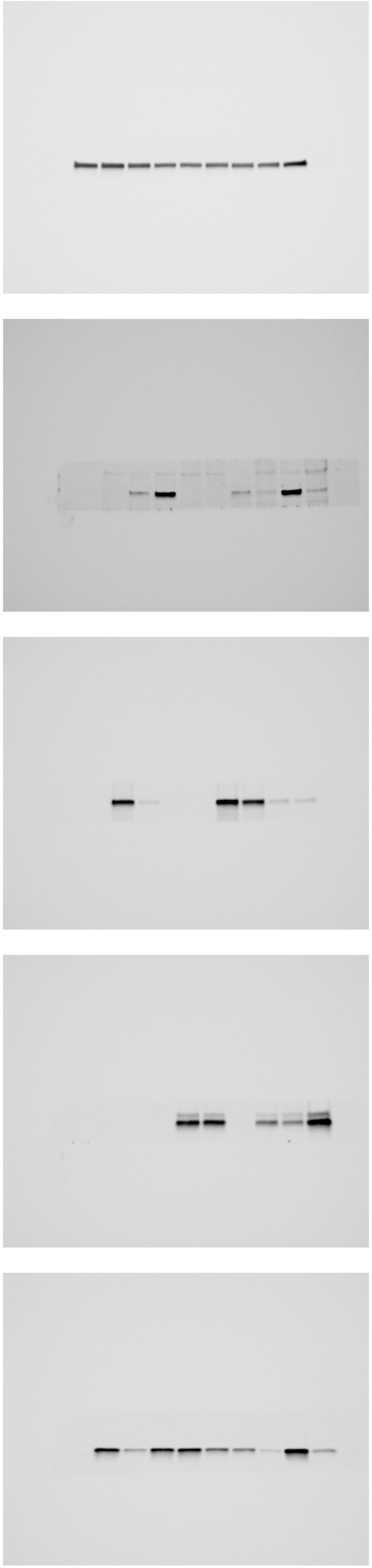

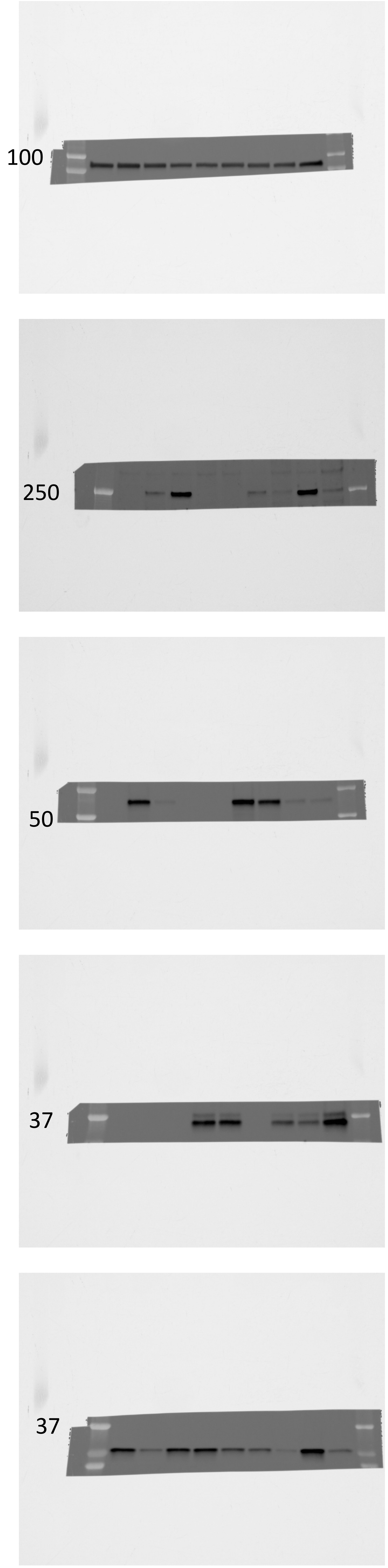

Ponceau S Staining

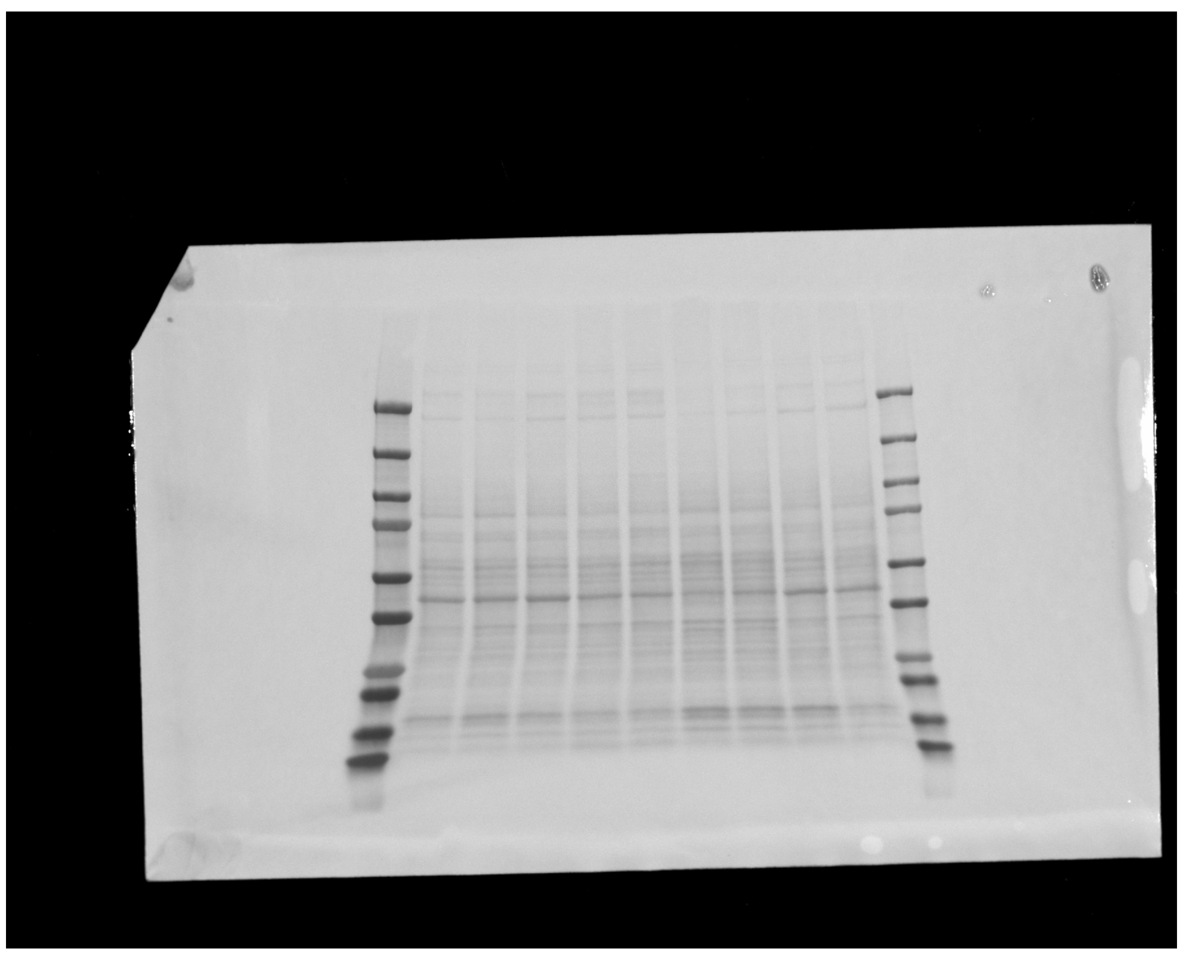

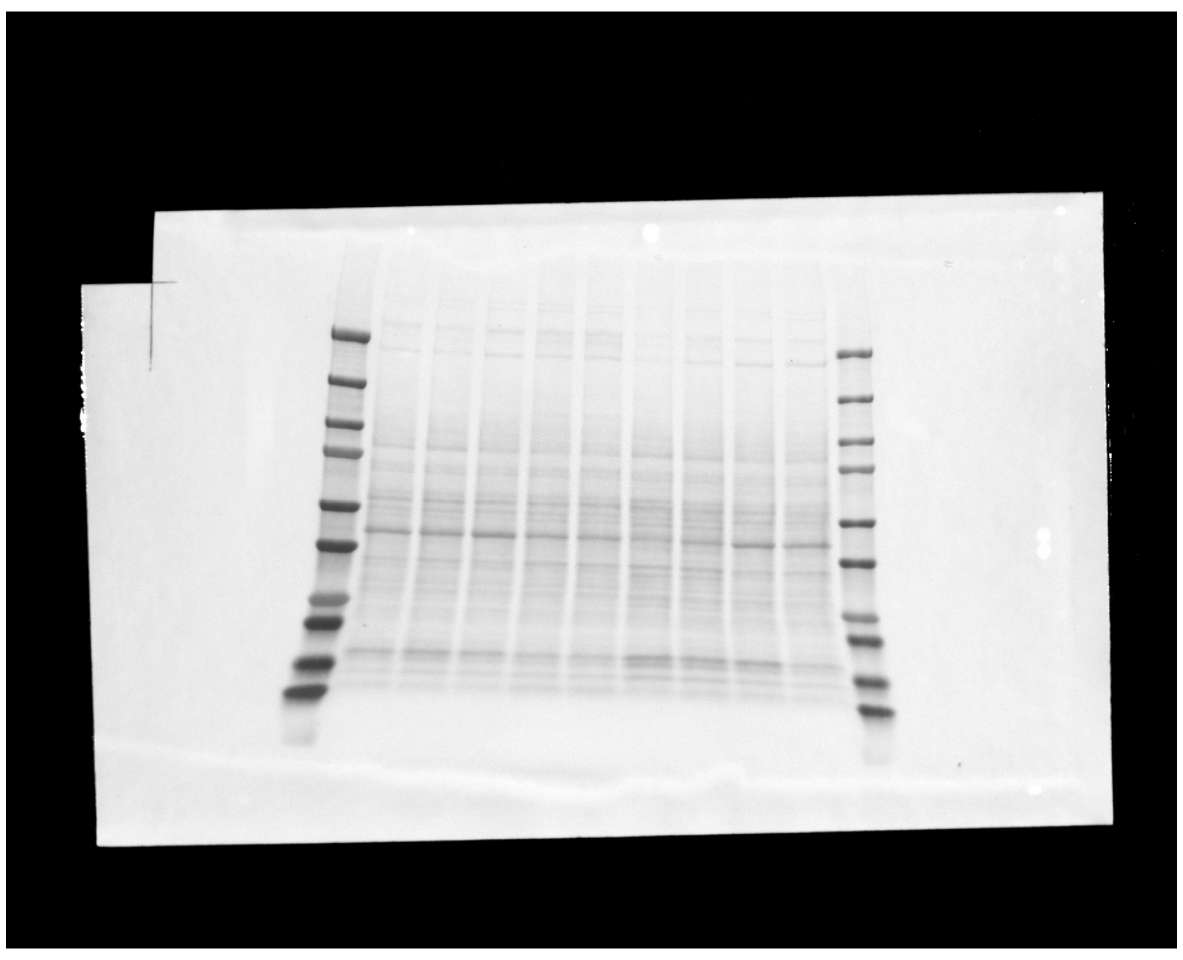

Note: HSP90 and GFP were cut from the same set of samples (right), and DUX4, ETV5, and Myogenin were cut from the same set of samples (left).

Sample order, left to right:
7 uL ladder
EV C7 d0
CD4 D6 d0
dC1 C5 d0
EV C7 ctrl d5
EV C7 siGFP d5
CD4 D6 ctrl d5
CD4 D6 siGFP d5
dC1 C5 ctrl d5
dC1 C5 siGFP d5
3 uL ladder

Figure 5 Panel C

Uncropped Chemiluminescence Image

DUX4

ETV5

GFP

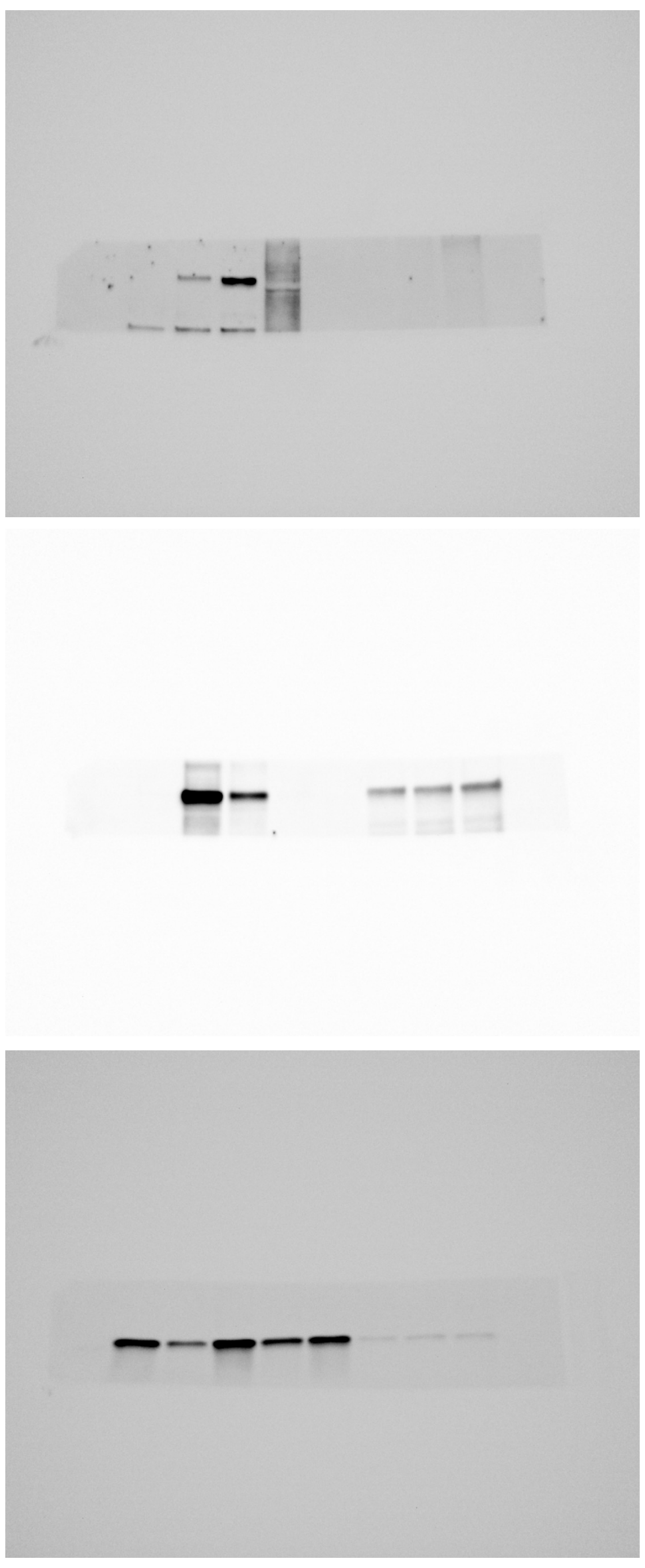

Uncropped Composite (with Colorimetric, for ladder)

250

50

37

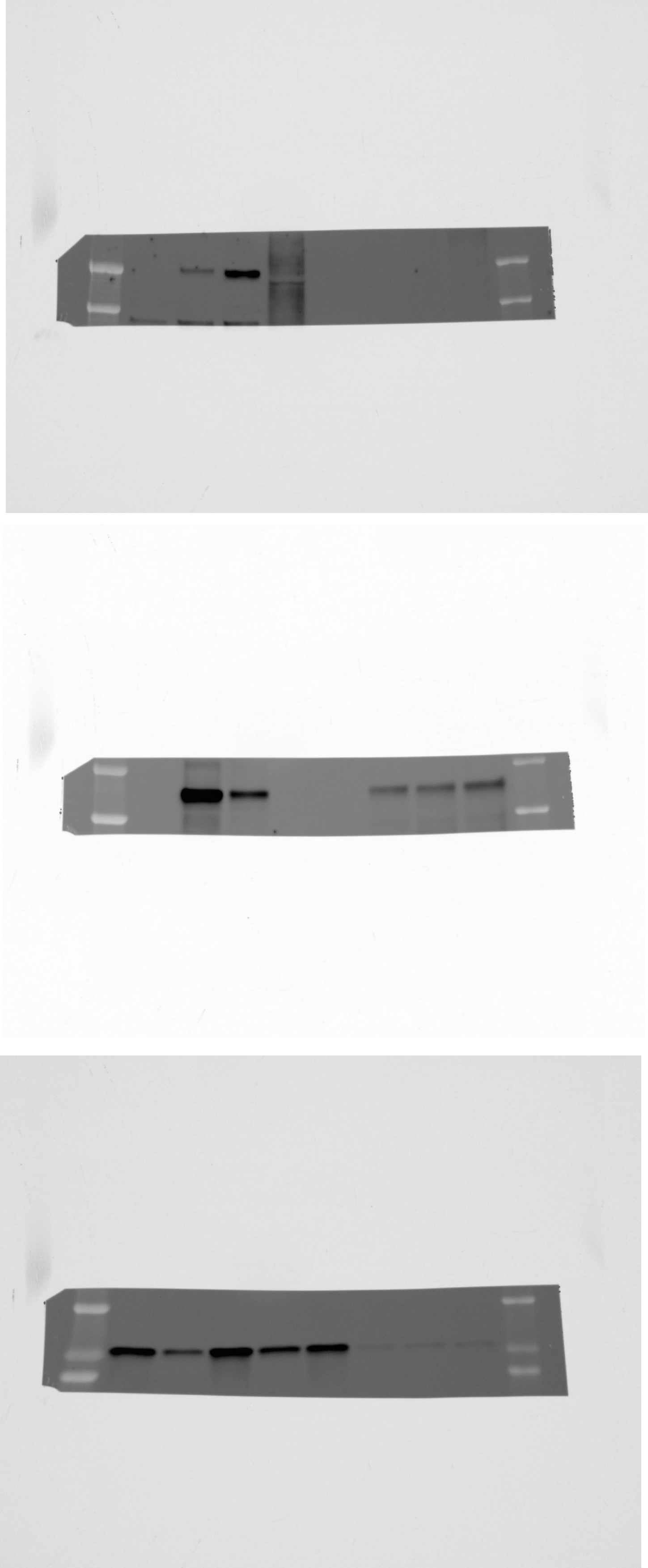

Ponceau S Staining

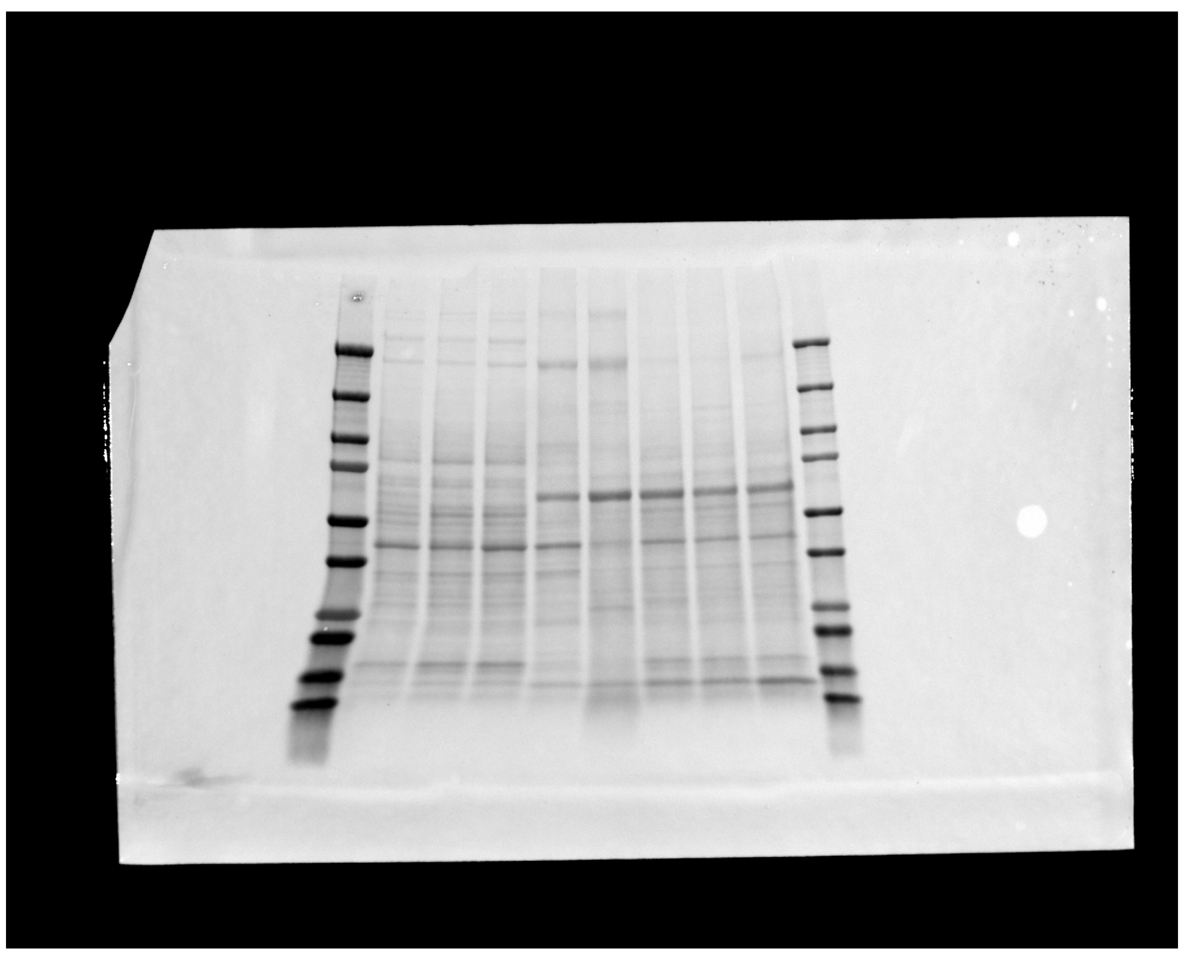

Sample order, left to right:
7 uL ladder
C2C12 EV G4
C2C12 CD4 C9
C2C12 dC1 D5
EV M3-L
dC1 M2-L
CD4 M2-R
CD4 M4-L
CD4 M5-L
3 uL ladder

Figure 5 Panel D

Uncropped Image

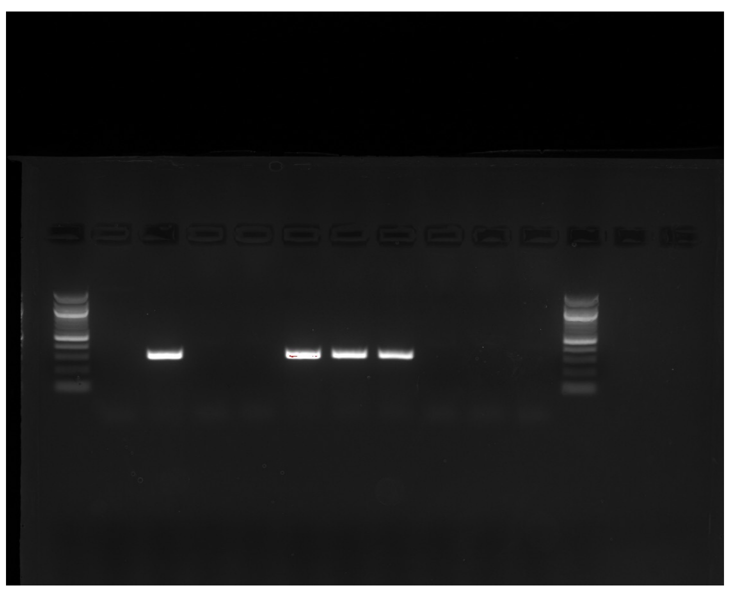

EGFP Products

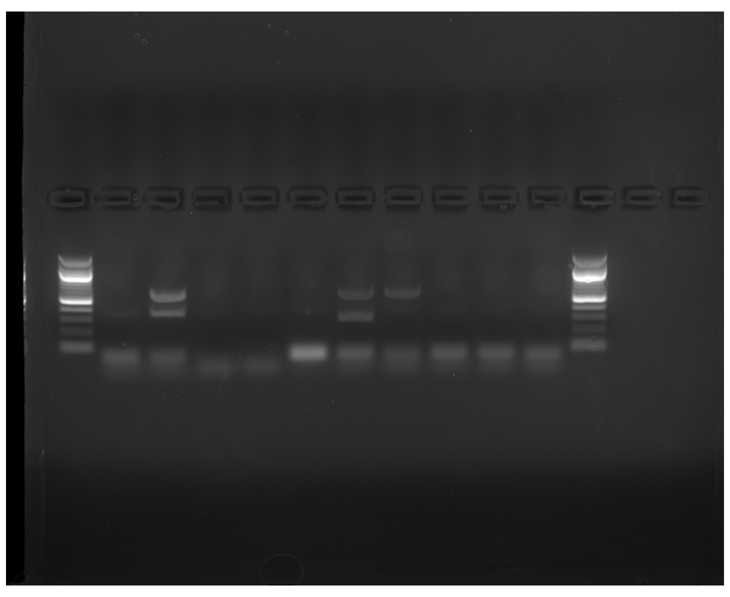

CIC-DUX4 Products

Sample order, left to right:
100bp ladder (NEB Quick-Load)
C2C12 plain +RT
C2C12 CD4 C9 +RT
C2C12 plain –RT
C2C12 CD4 C9 –RT
EV M2-R +RT
CD4 M1-R +RT
CD4 M3-R +RT
EV M2-R –RT
CD4 M1-R –RT
CD4 M3-R –RT
100bp ladder (NEB Quick-Load)

2% agarose gel, run at 100V, made with Ethidium Bromide
Both gels: 10 uL ladder loaded
EGFP reactions: 10 uL reaction loaded
CICDUX4 reactions: 20 uL reaction loaded

Supplemental Figure S1 Panel C

Uncropped Chemiluminescence Image

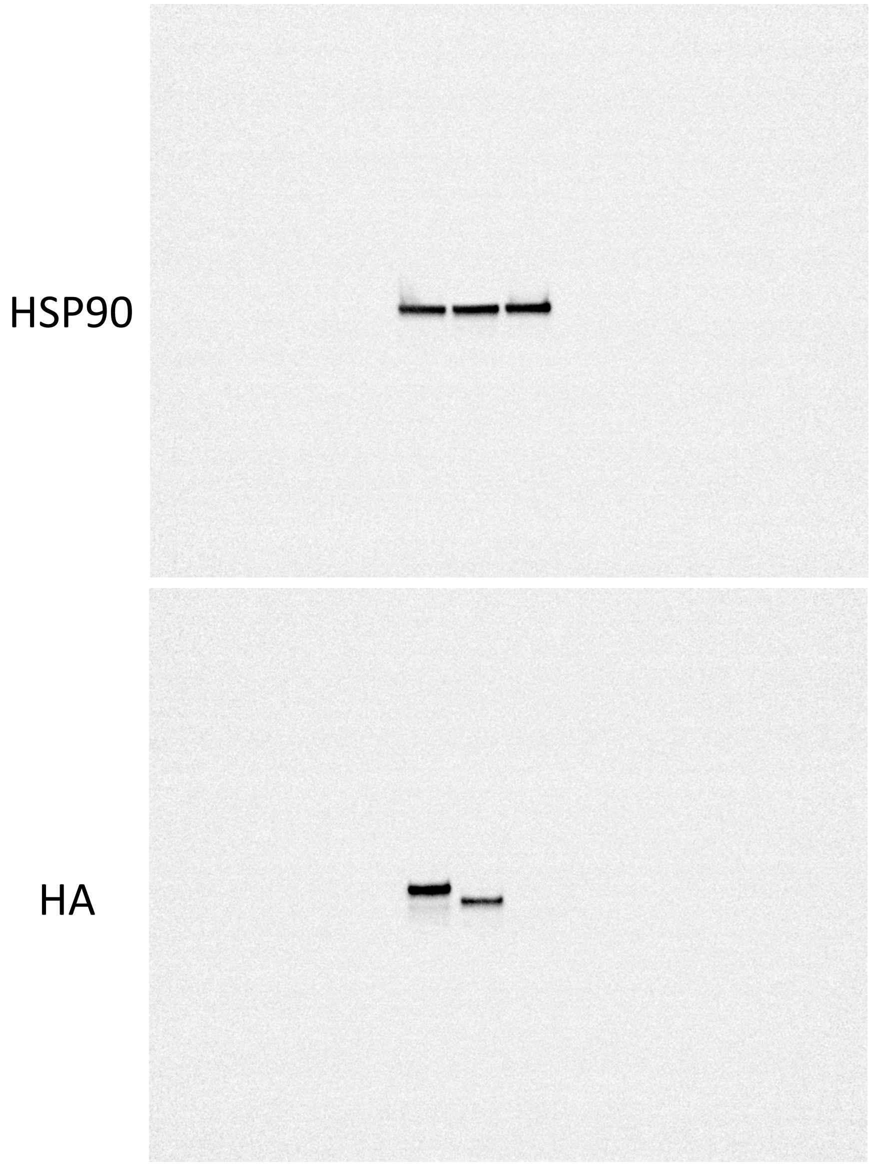

Uncropped Composite (with Colorimetric, for ladder)

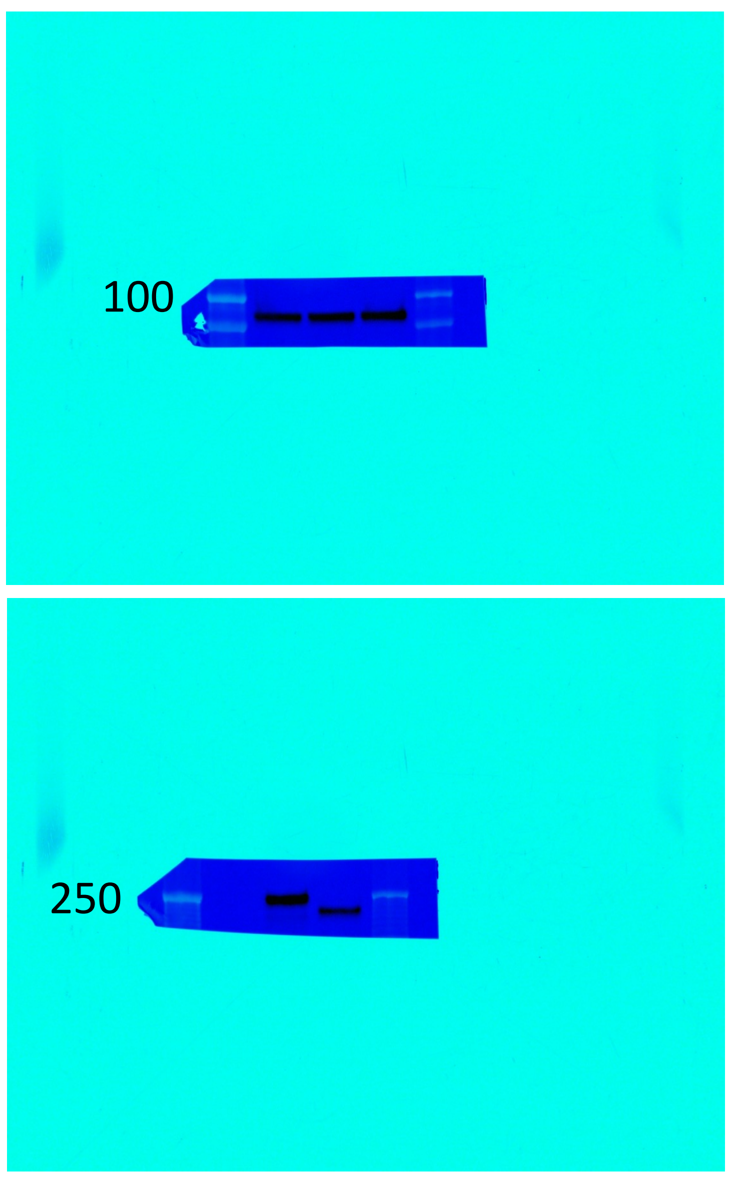

Ponceau S Staining

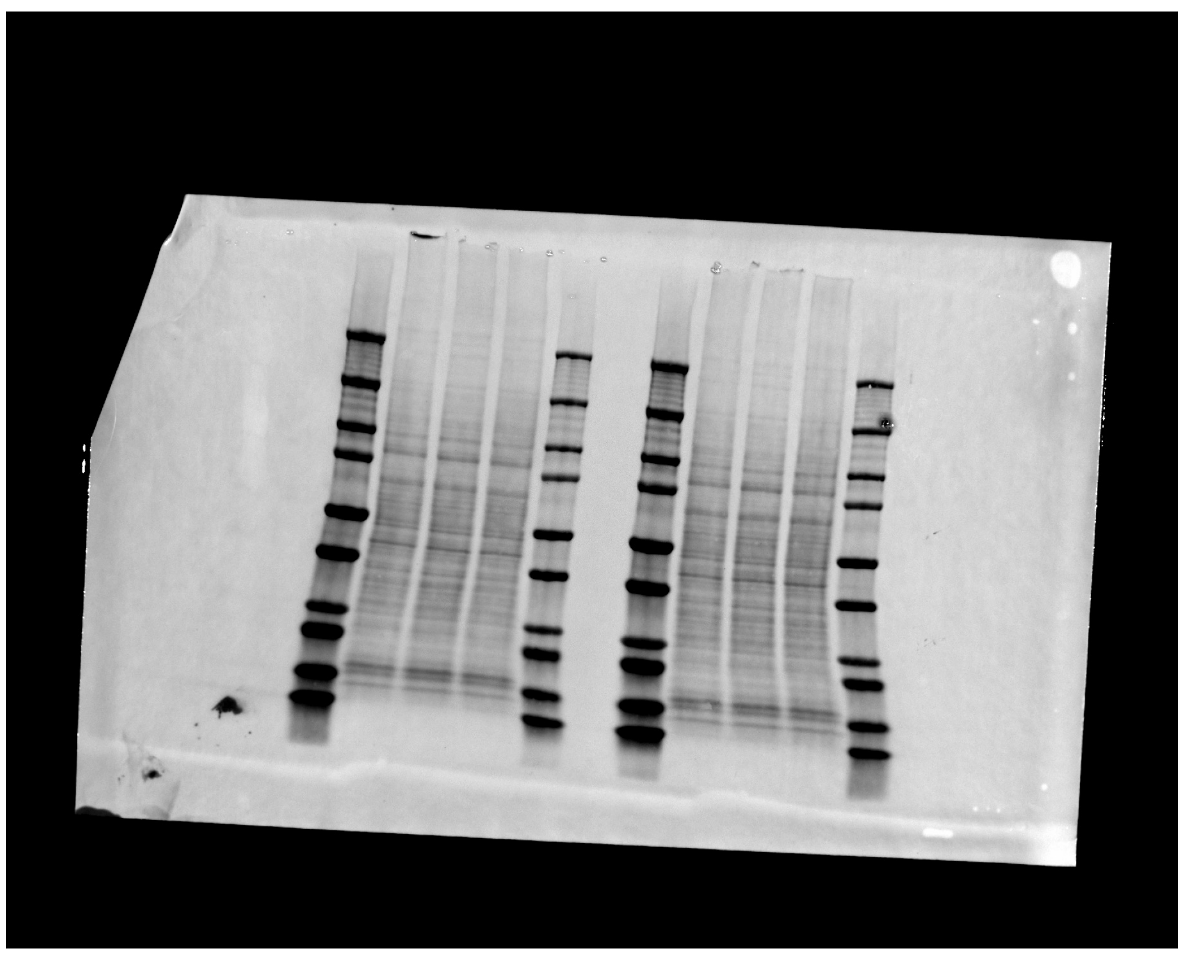

Note: both HSP90 and HA were cut from the same set of samples, the other was for a different blot.

Sample order, left to right:
7 uL ladder
EV
HA-CIC::DUX4
HA-CIC::UTR
3 uL ladder

Supplemental Figure S3 Panel A, Gel 1 (left)

Uncropped Chemiluminescence Image

Uncropped Composite (with Colorimetric, for ladder)

HSP90

DUX4

ETV5

GFP

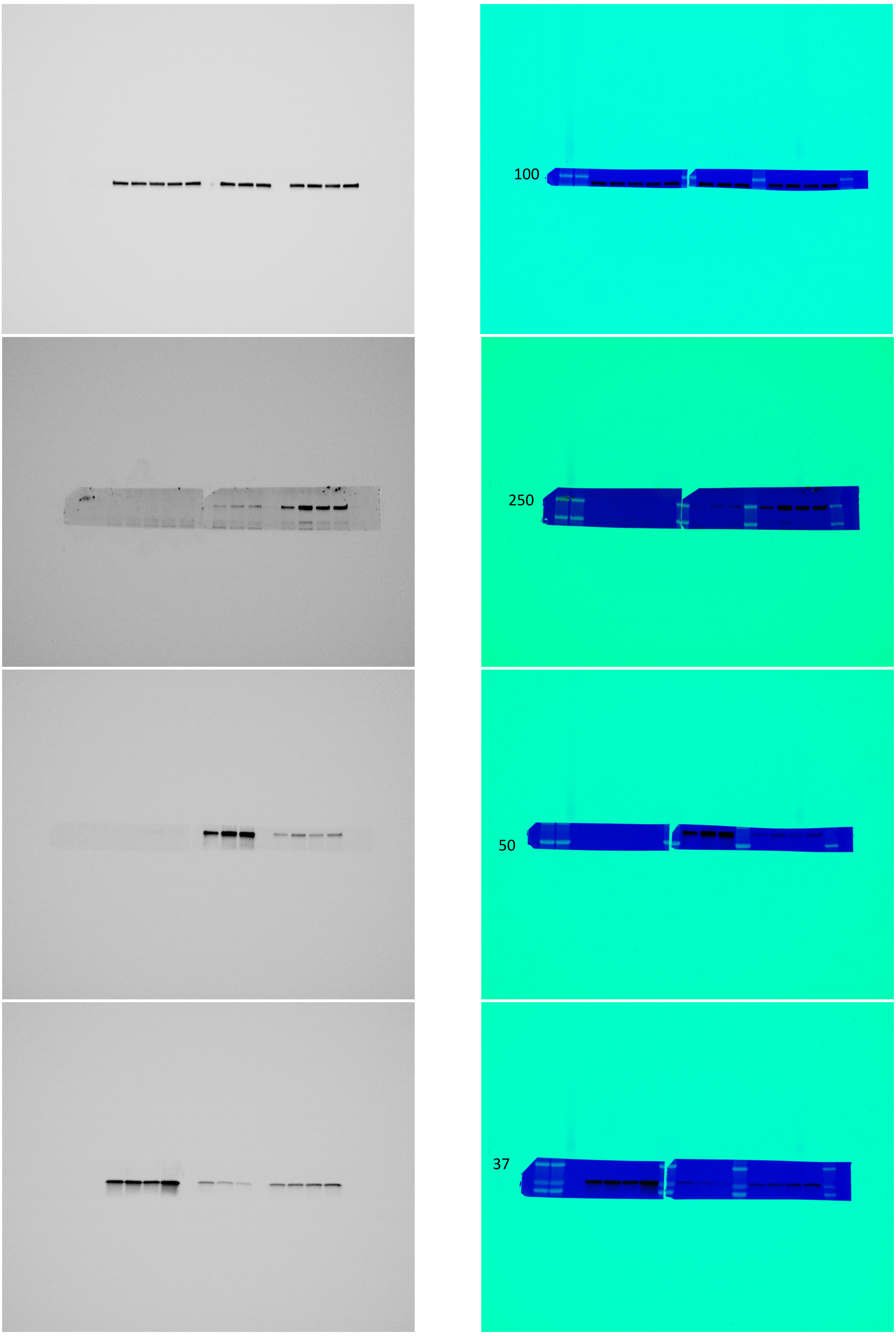

Ponceau S Staining

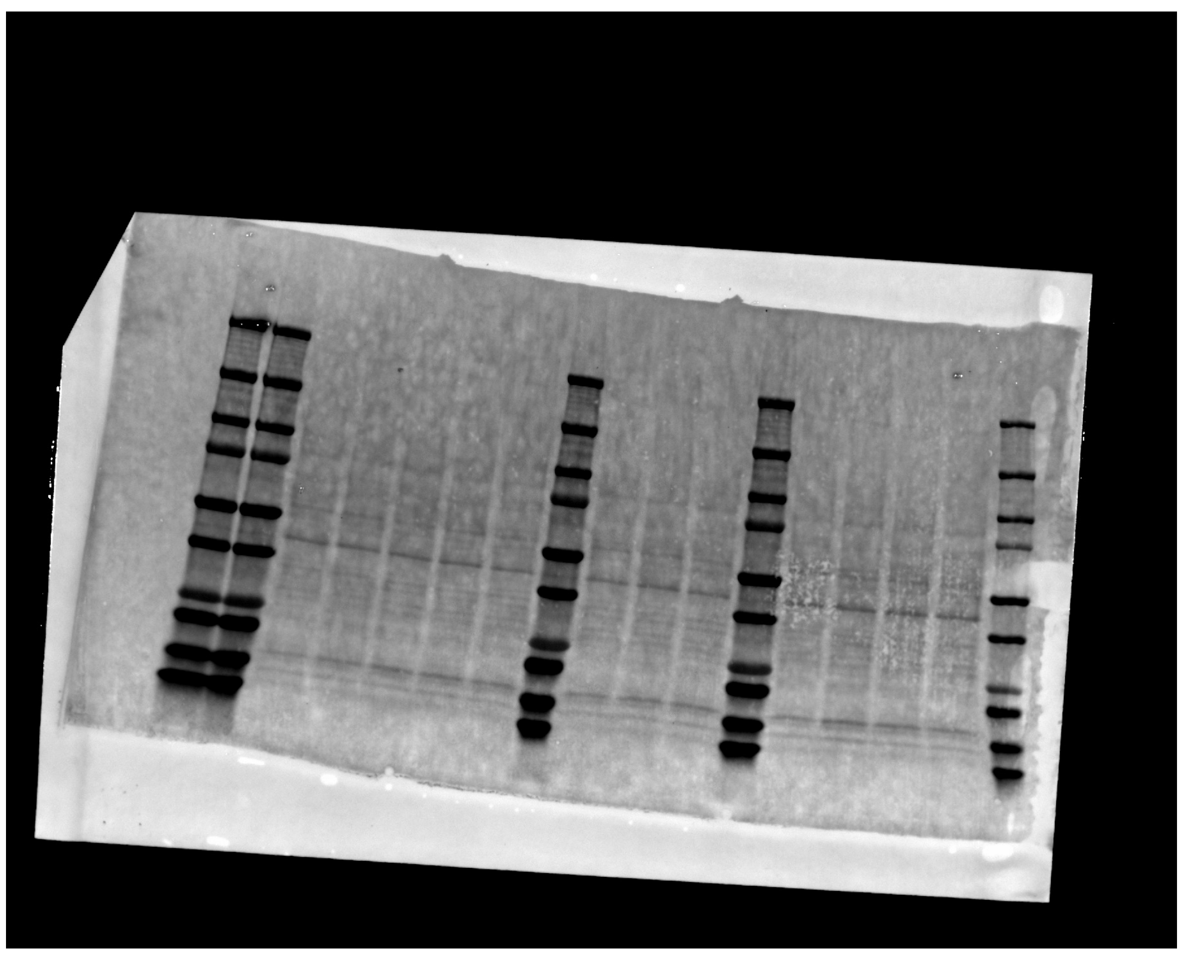

Sample order, left to right:
7 uL ladder
7 uL ladder
Plain
Bulk EV
EV B4
EV B6
EV C2
7 uL ladder
Bulk CD4
CD4 C7
CD4 C9
7 uL ladder
Bulk dC1
dC1 B4
dC1 B5
dC1 C9
3 uL ladder

Supplemental Figure S3 Panel A, Gel 2 (right)

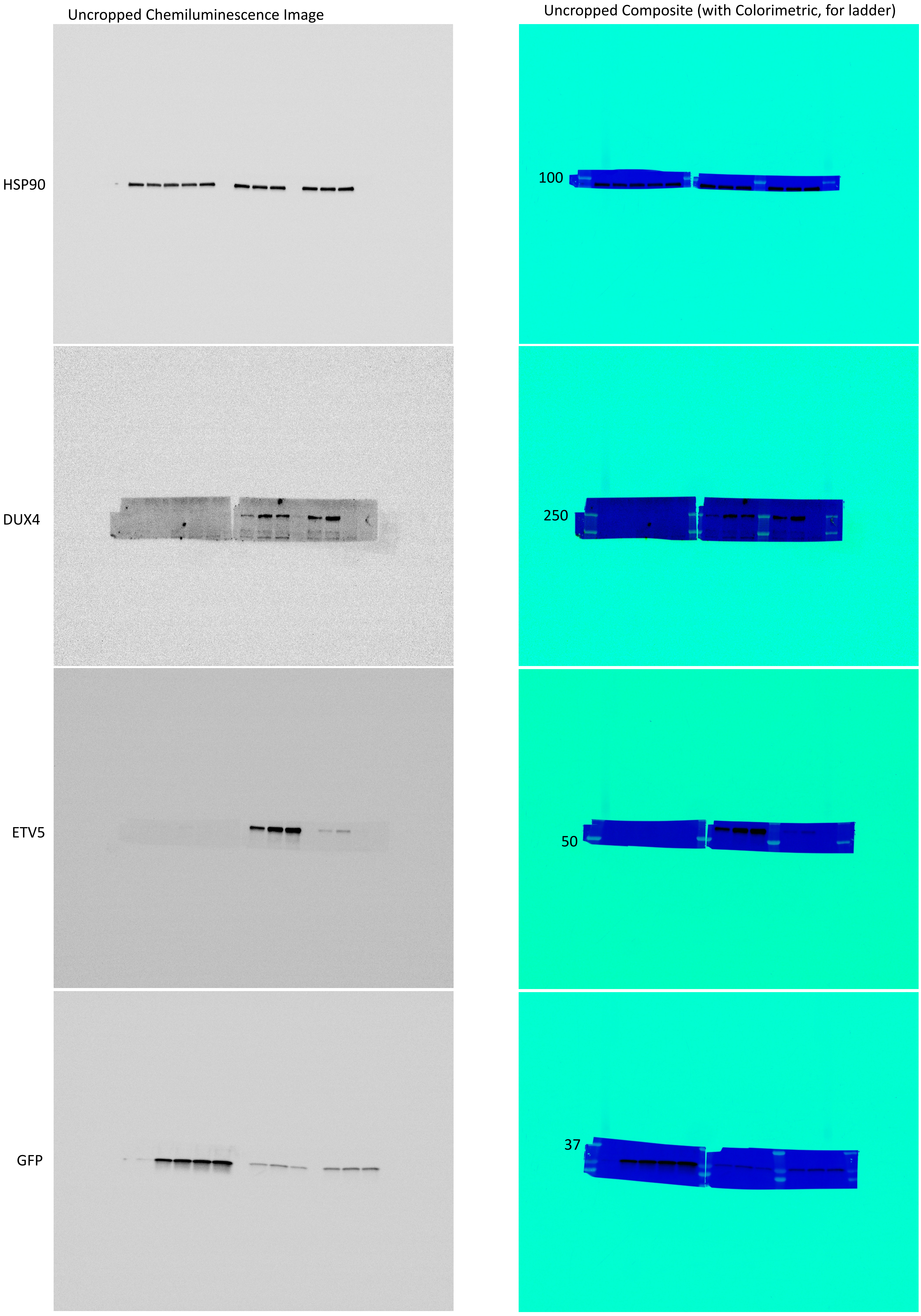

Ponceau S Staining

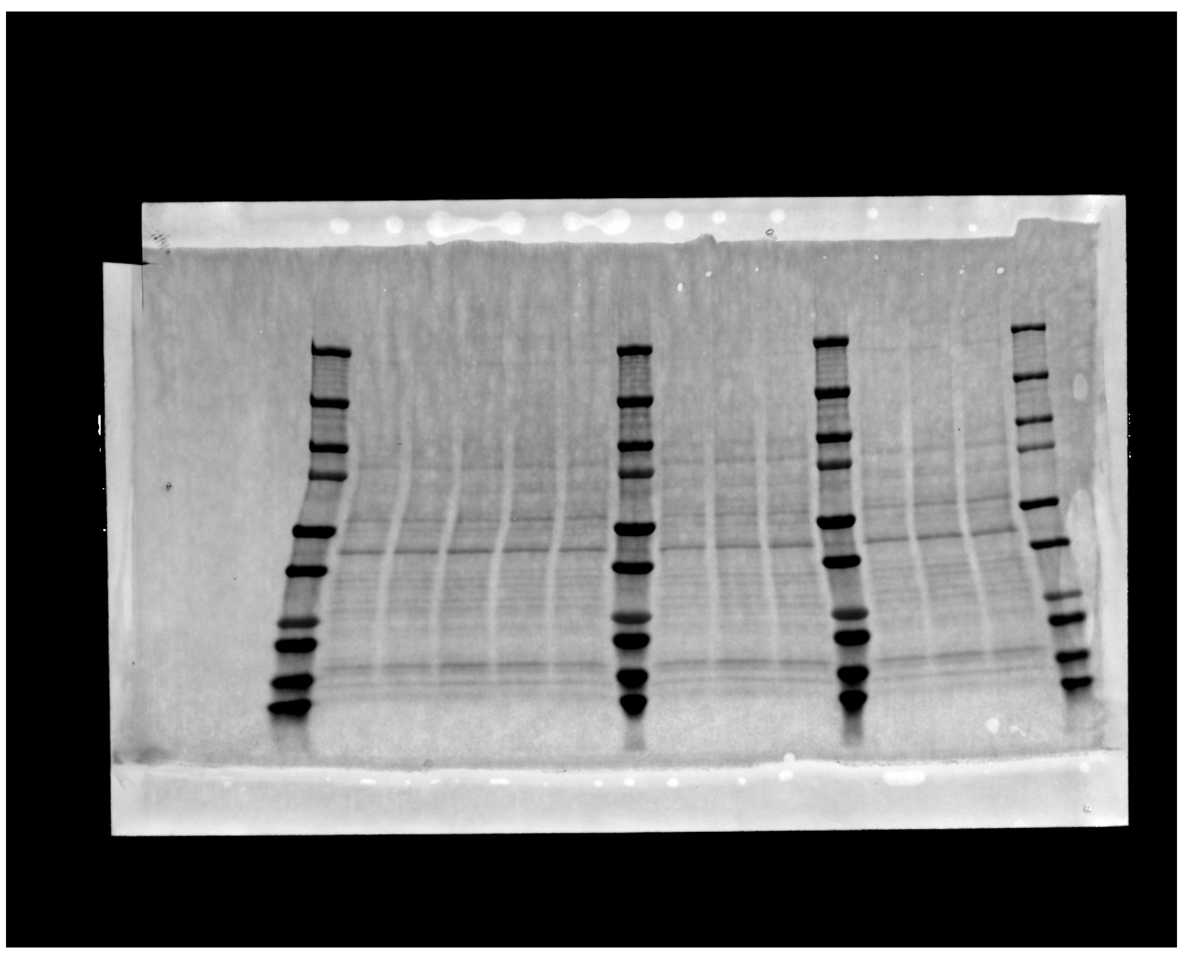

Sample order, left to right:
7 uL ladder
Plain
Bulk EV
EV D8
EV E8
EV F11
7 uL ladder
Bulk CD4
CD4 D4
CD4 D8
7 uL ladder
Bulk dC1
dC1 C10
dC1 E10
3 uL ladder

Supplemental Figure S3 Panel C, Gel 1 (left)

Uncropped Chemiluminescence Image

Uncropped Composite (with Colorimetric, for ladder)

HSP90

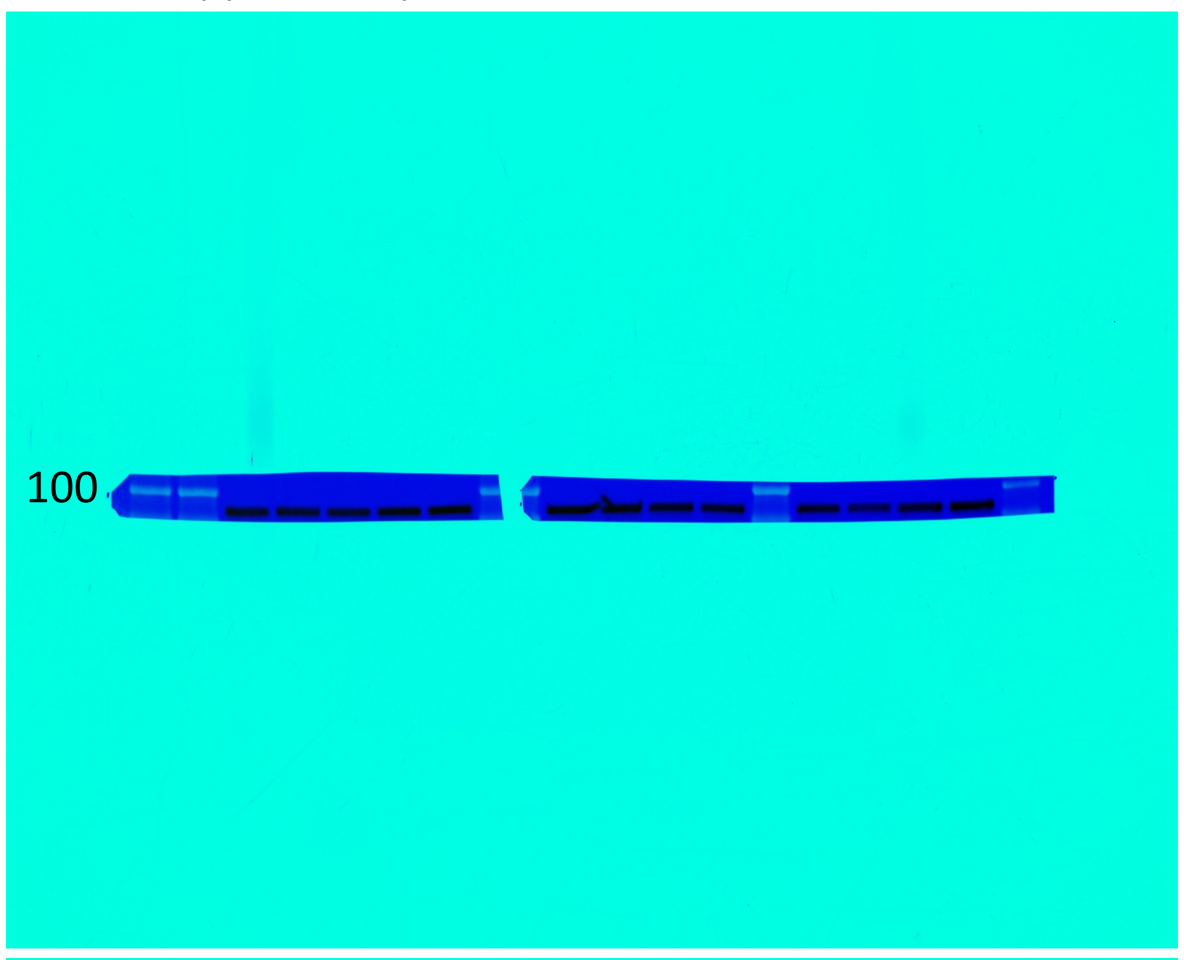

DUX4 short exposure

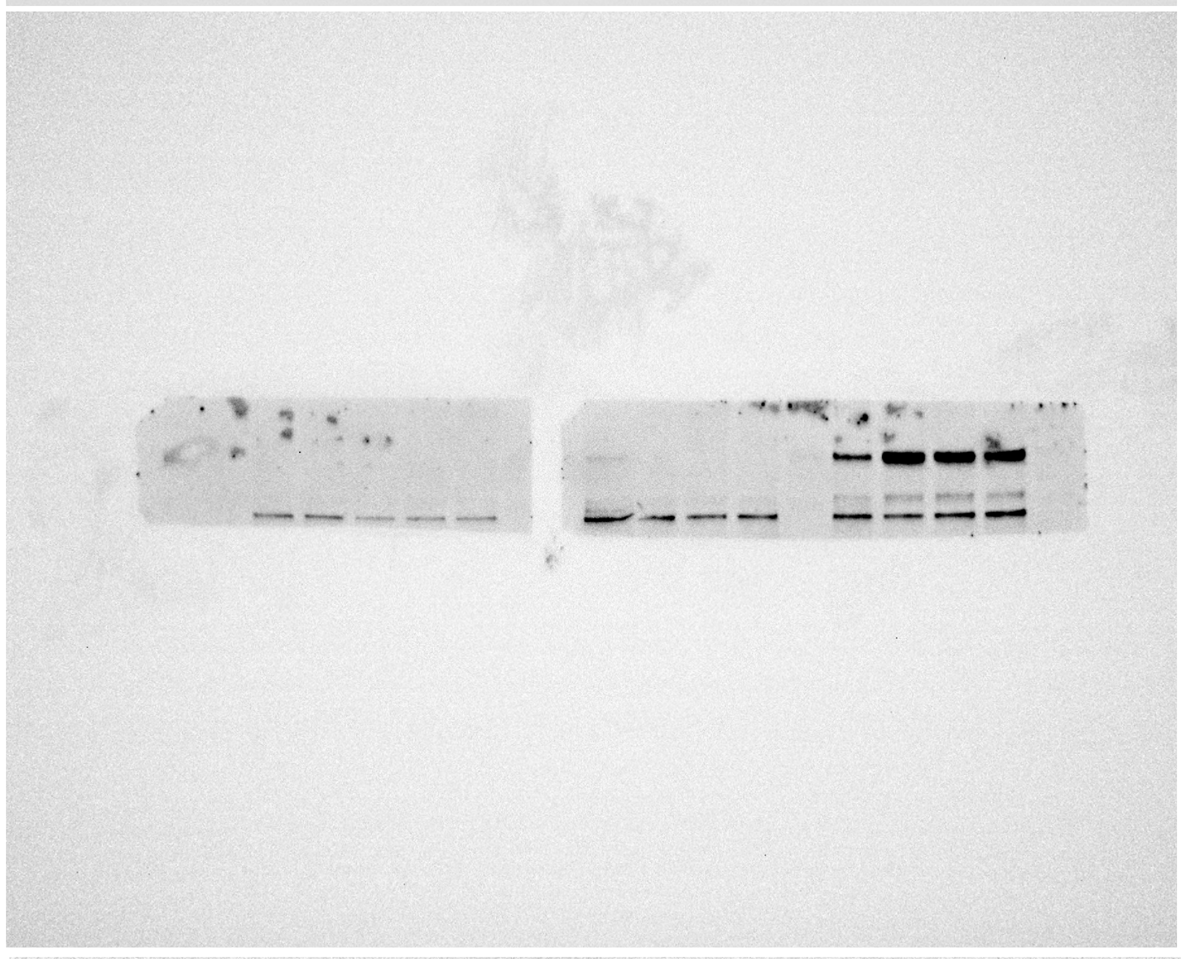

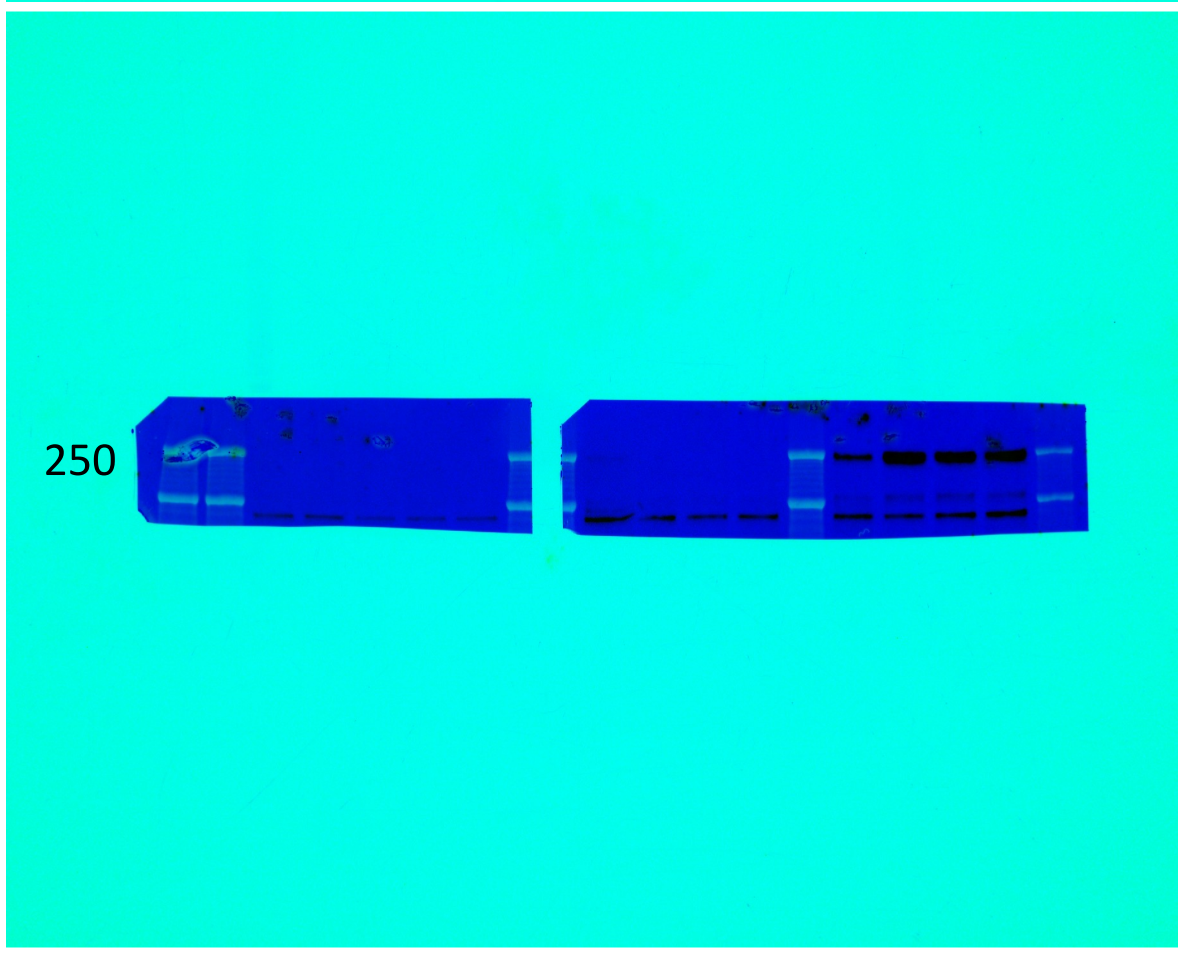

DUX4 long exposure

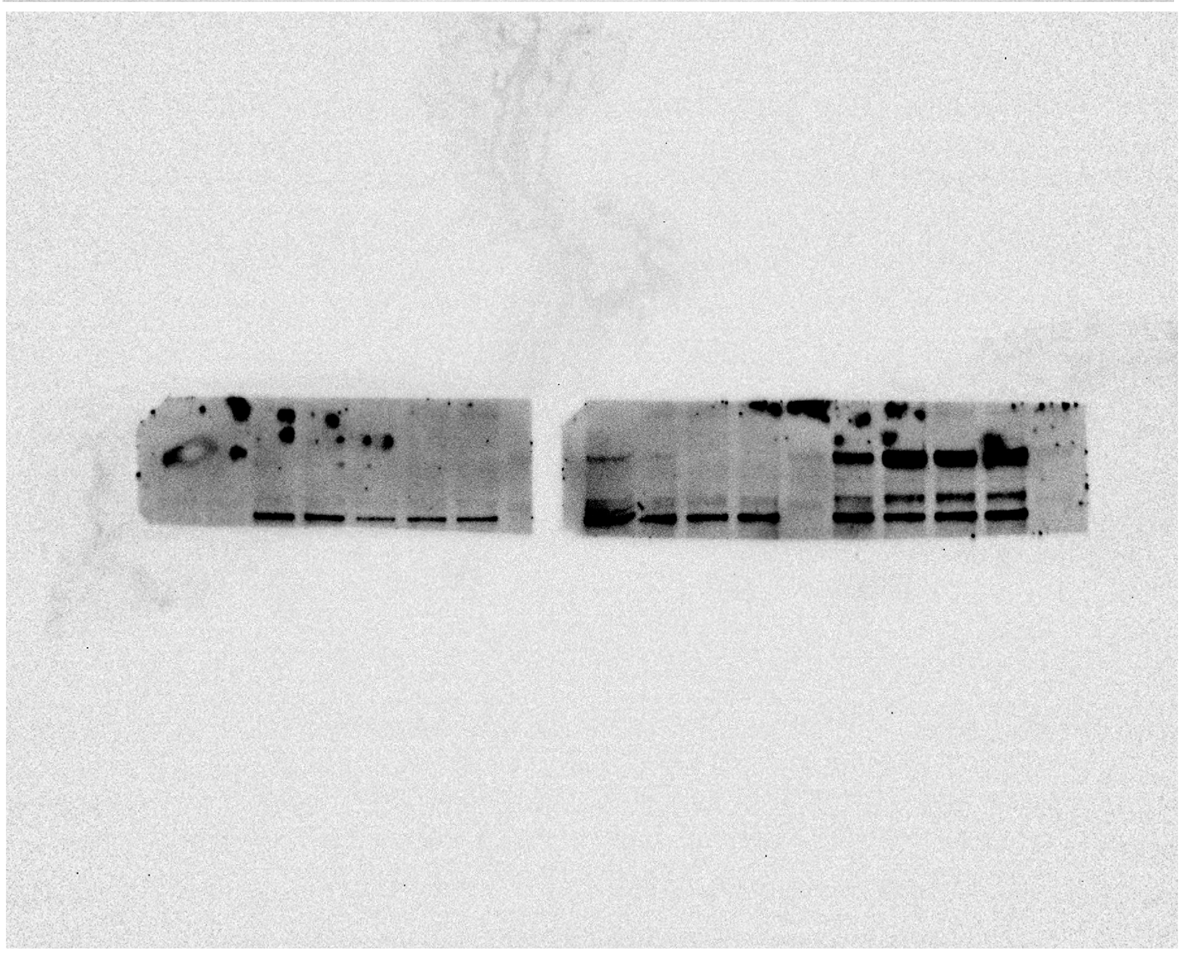

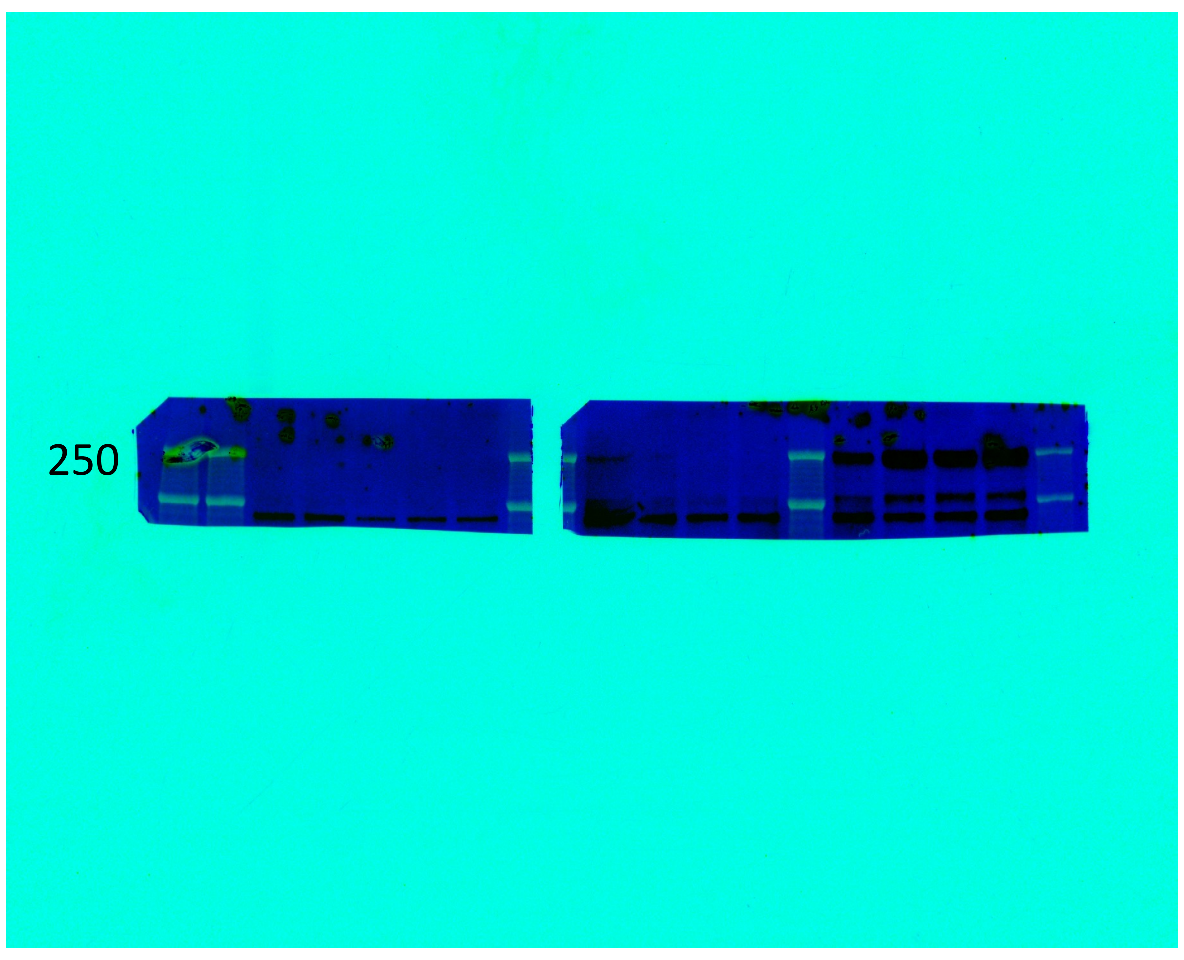

Blots continued on next slide -- ETV5, GFP

Ponceau S Staining

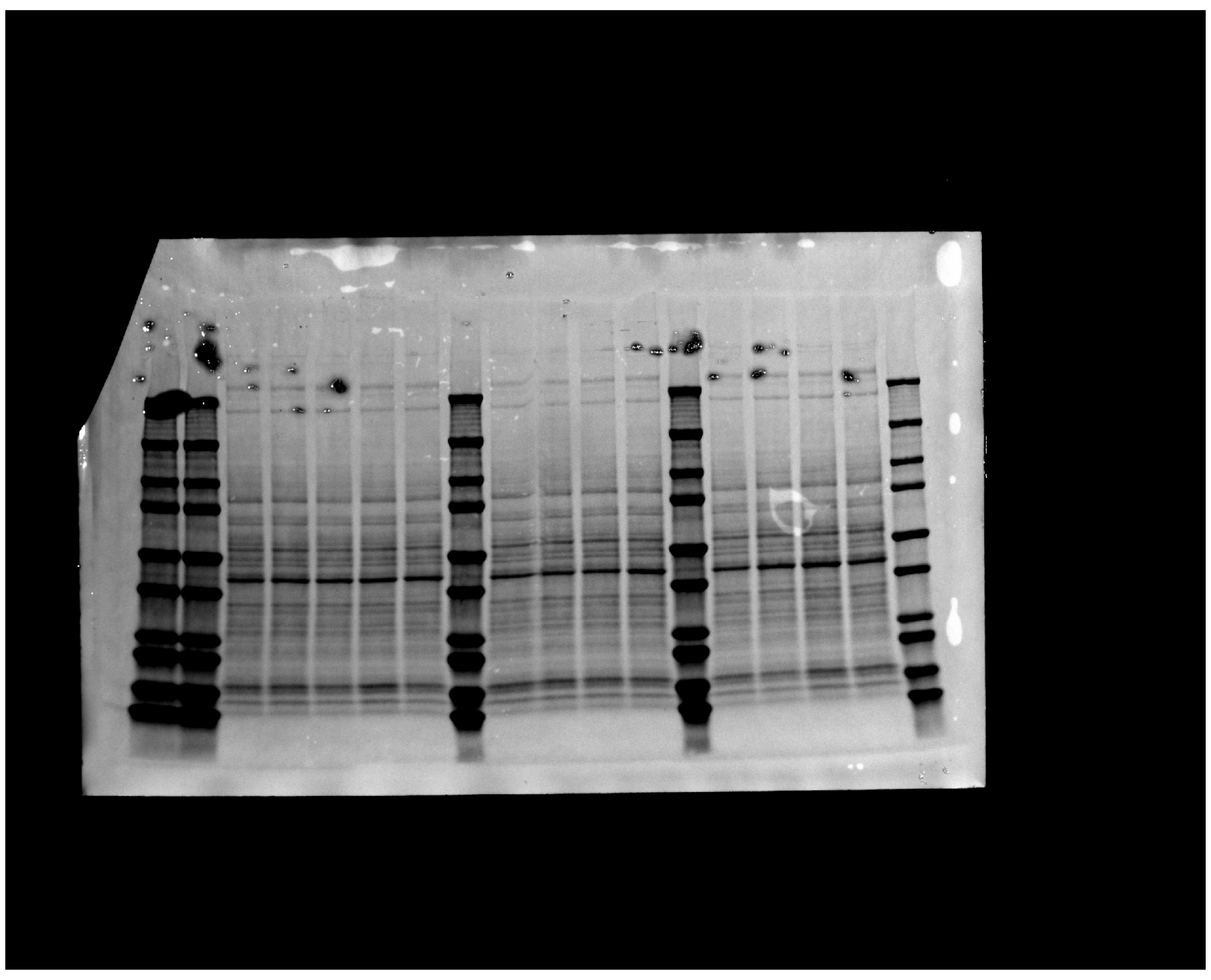

Sample order, left to right:
7 uL ladder
7 uL ladder
Plain
Bulk EV
EV B7
EV C7
EV E5
7 uL ladder
Bulk CICDUX4
CICDUX4 B5
CICDUX4 B7
CICDUX4 C7
7 uL ladder
Bulk dC1
dC1 B8
dC1 B9
dC1 B11
3 uL ladder

Supplemental Figure S3 Panel C, Gel 1 (left)
Continued from previous slide

Uncropped Chemiluminescence Image

ETV5

GFP

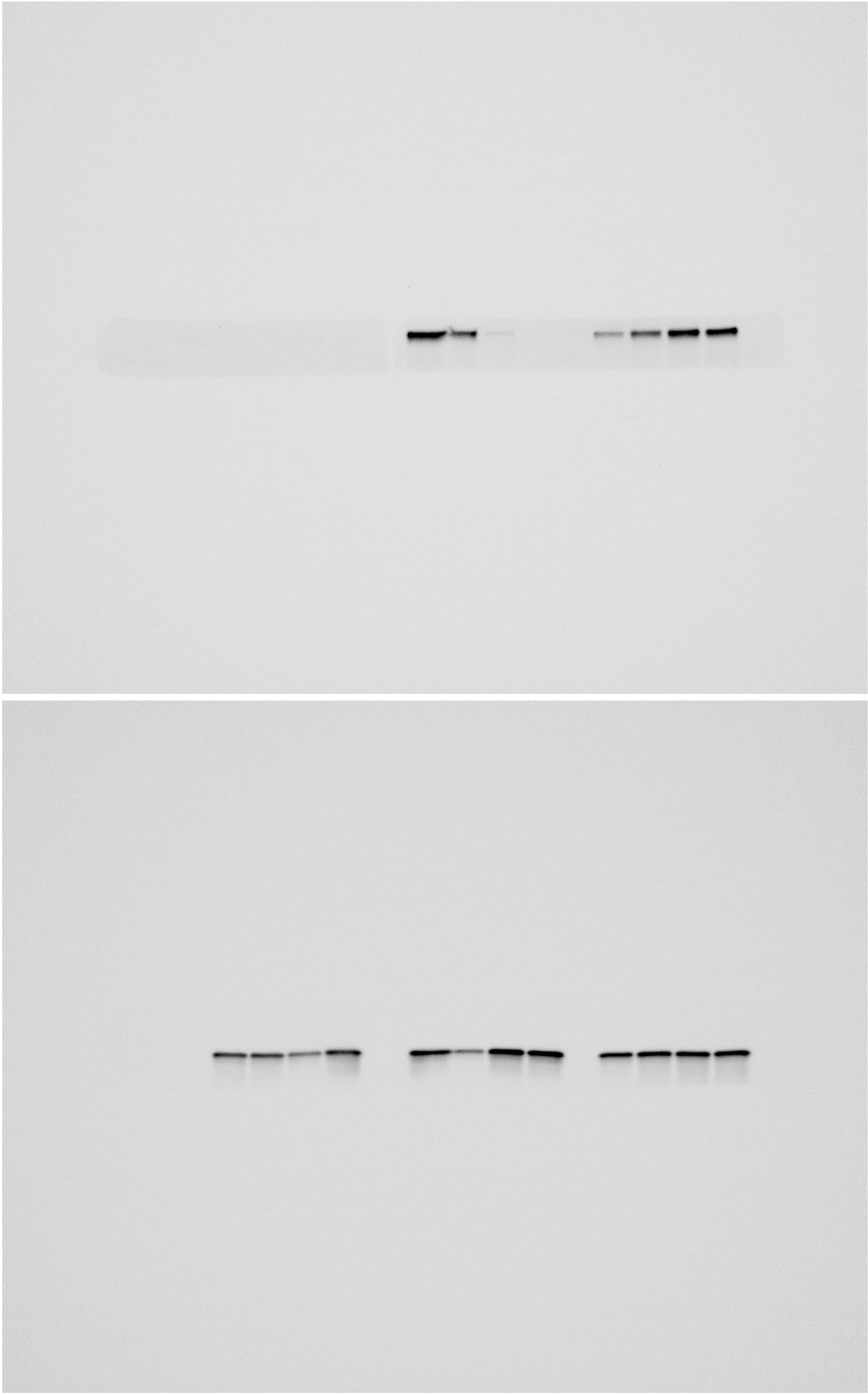

Uncropped Composite (with Colorimetric, for ladder)

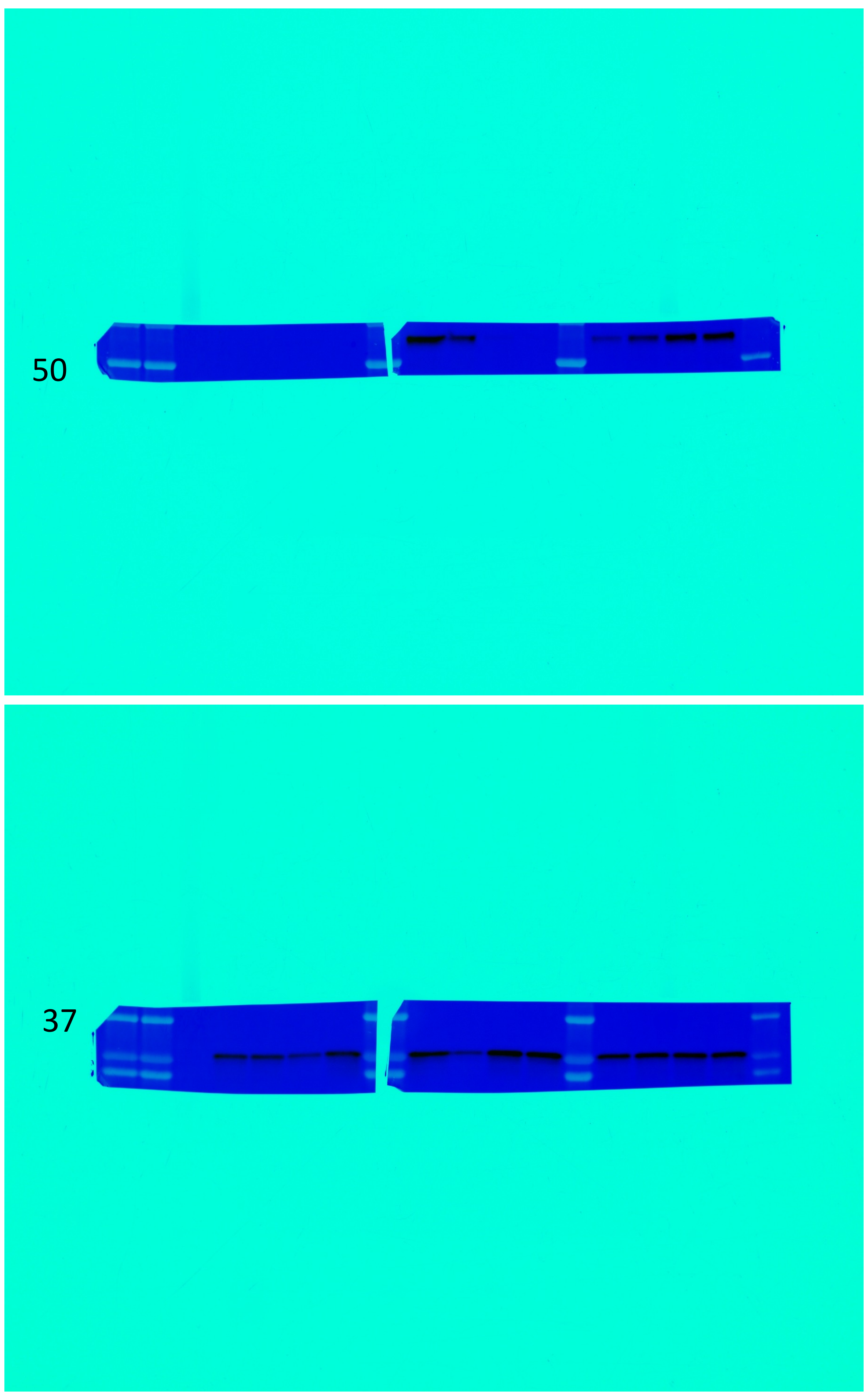

Ponceau S Staining

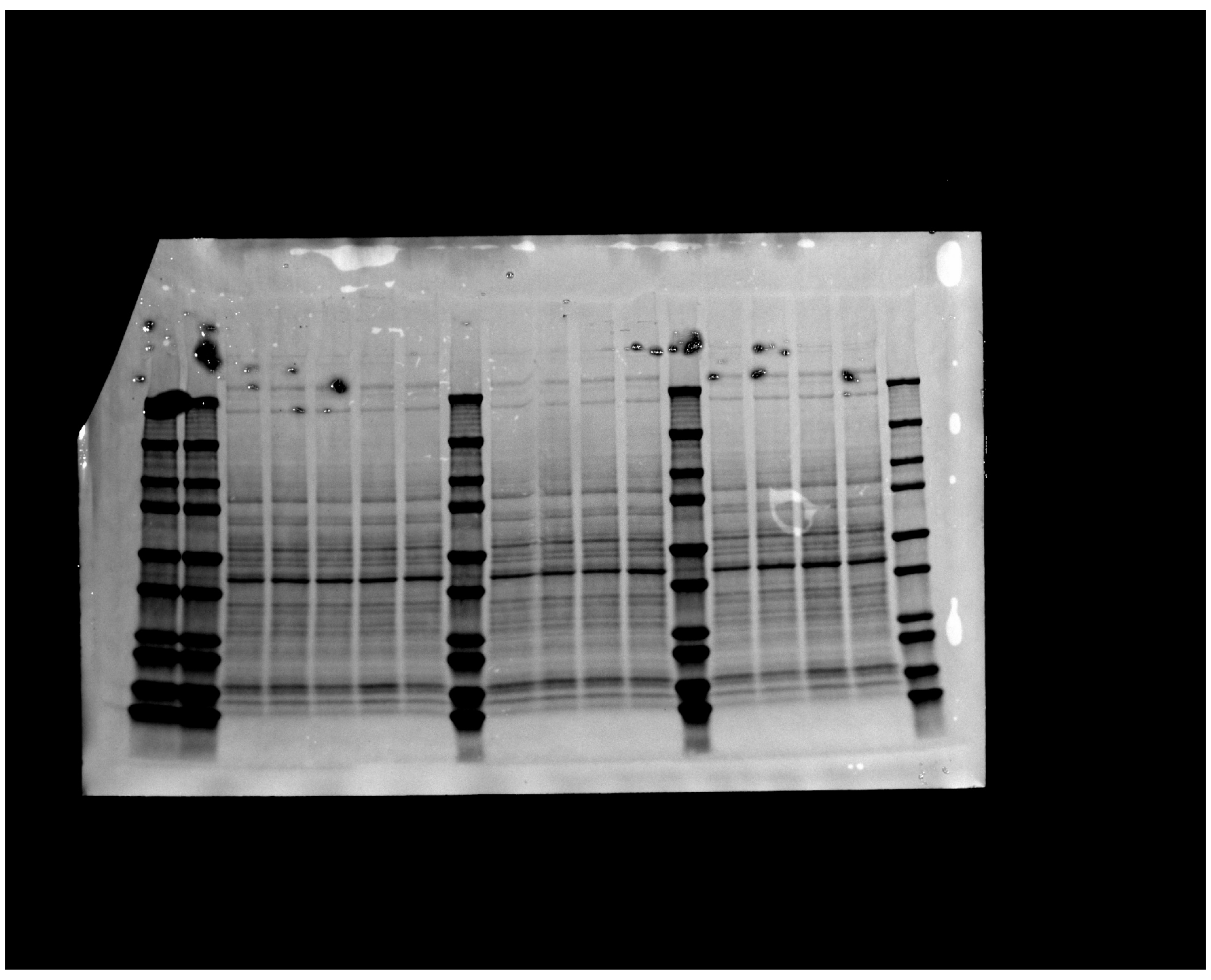

Sample order, left to right:
7 uL ladder
7 uL ladder
Plain
Bulk EV
EV B7
EV C7
EV E5
7 uL ladder
Bulk CICDUX4
CICDUX4 B5
CICDUX4 B7
CICDUX4 C7
7 uL ladder
Bulk dC1
dC1 B8
dC1 B9
dC1 B11
3 uL ladder

Supplemental Figure S3 Panel C, Gel 2 (right)

Uncropped Chemiluminescence Image

HSP90

DUX4 short exposure

DUX4 long exposure

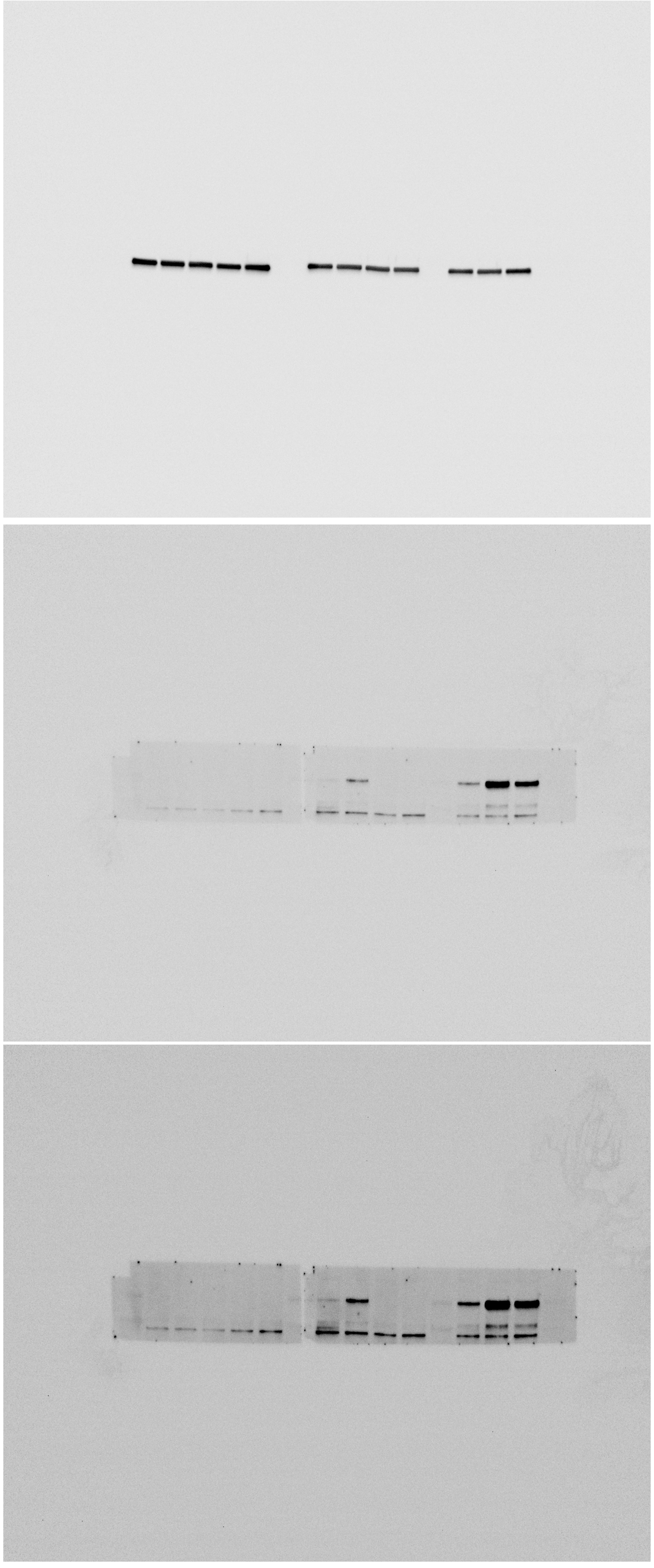

Uncropped Composite (with Colorimetric, for ladder)

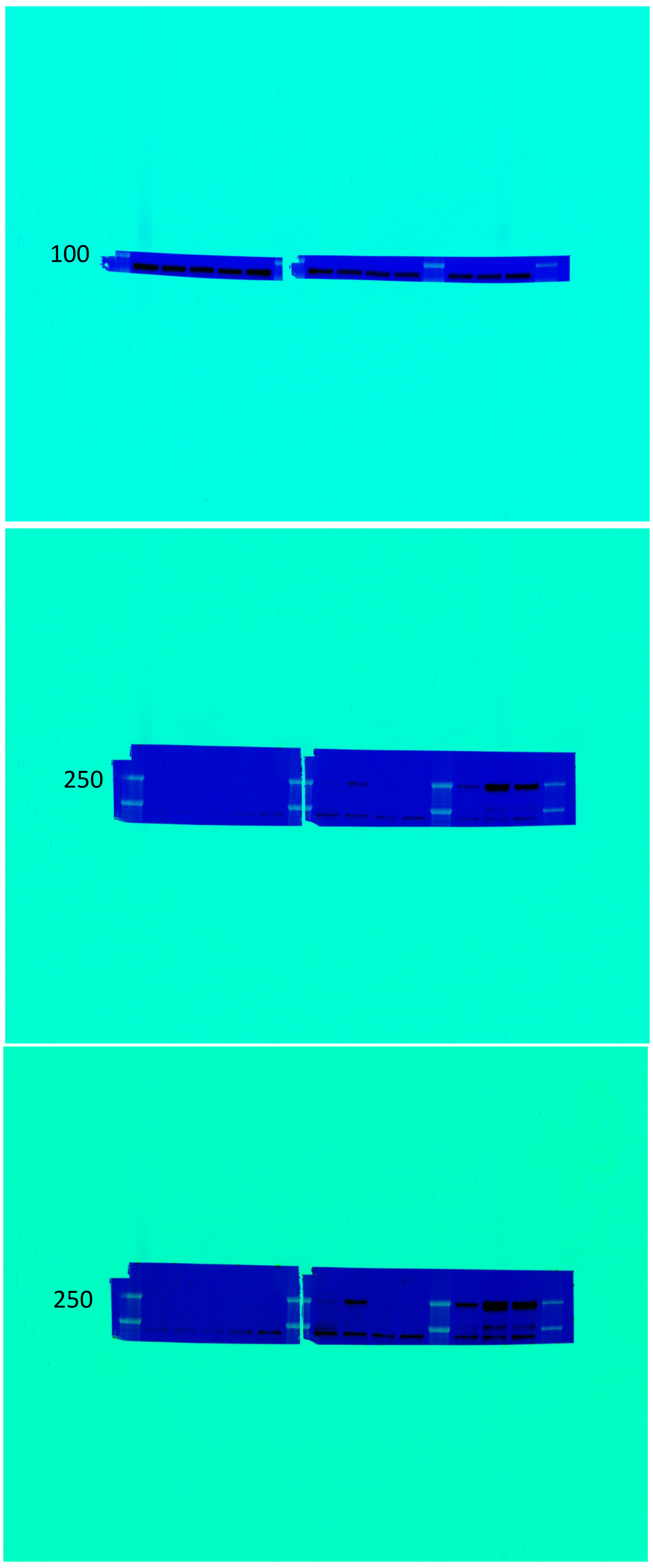

Blots continued on next slide -- ETV5, GFP

Ponceau S Staining

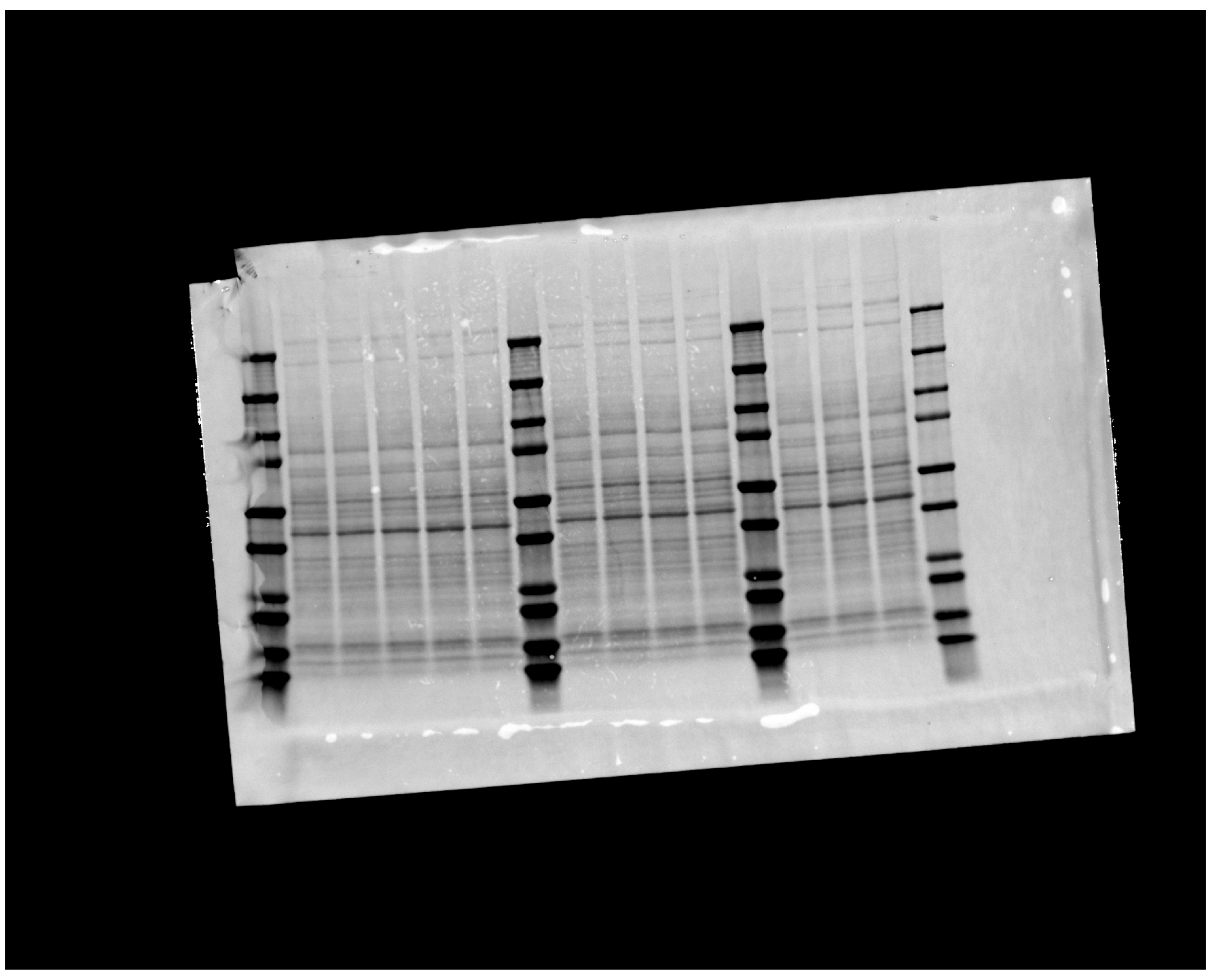

Sample order, left to right:
7 uL ladder
Plain
Bulk EV
EV E7
EV G4
EV G11
7 uL ladder
Bulk CICDUX4
CICDUX4 C9
CICDUX4 E10
CICDUX4 G8
7 uL ladder
Bulk dC1
dC1 C5
dC1 D5
3 uL ladder

Supplemental Figure S3 Panel C, Gel 2 (right)
Continued from previous slide

Uncropped Chemiluminescence Image

Uncropped Composite (with Colorimetric, for ladder)

ETV5

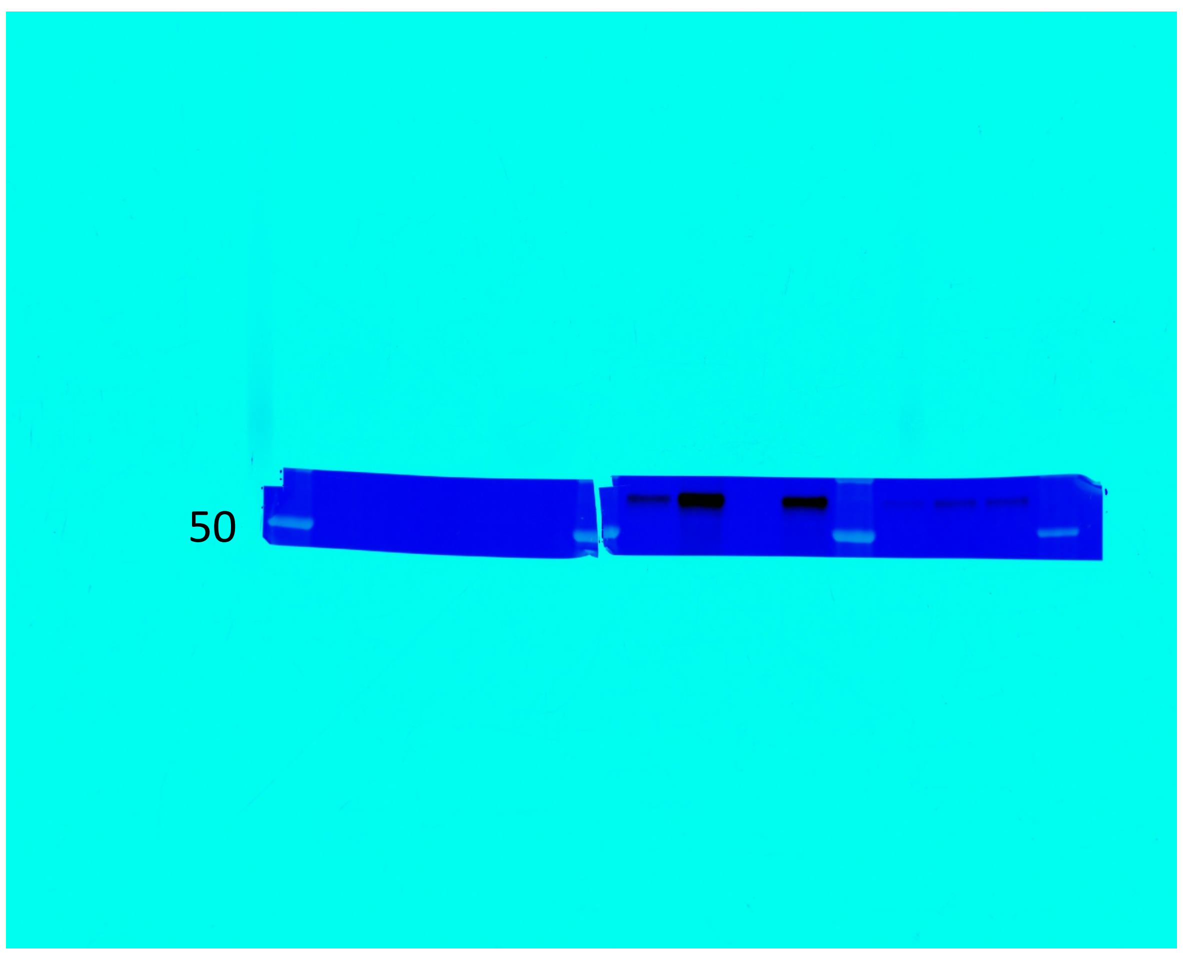

GFP

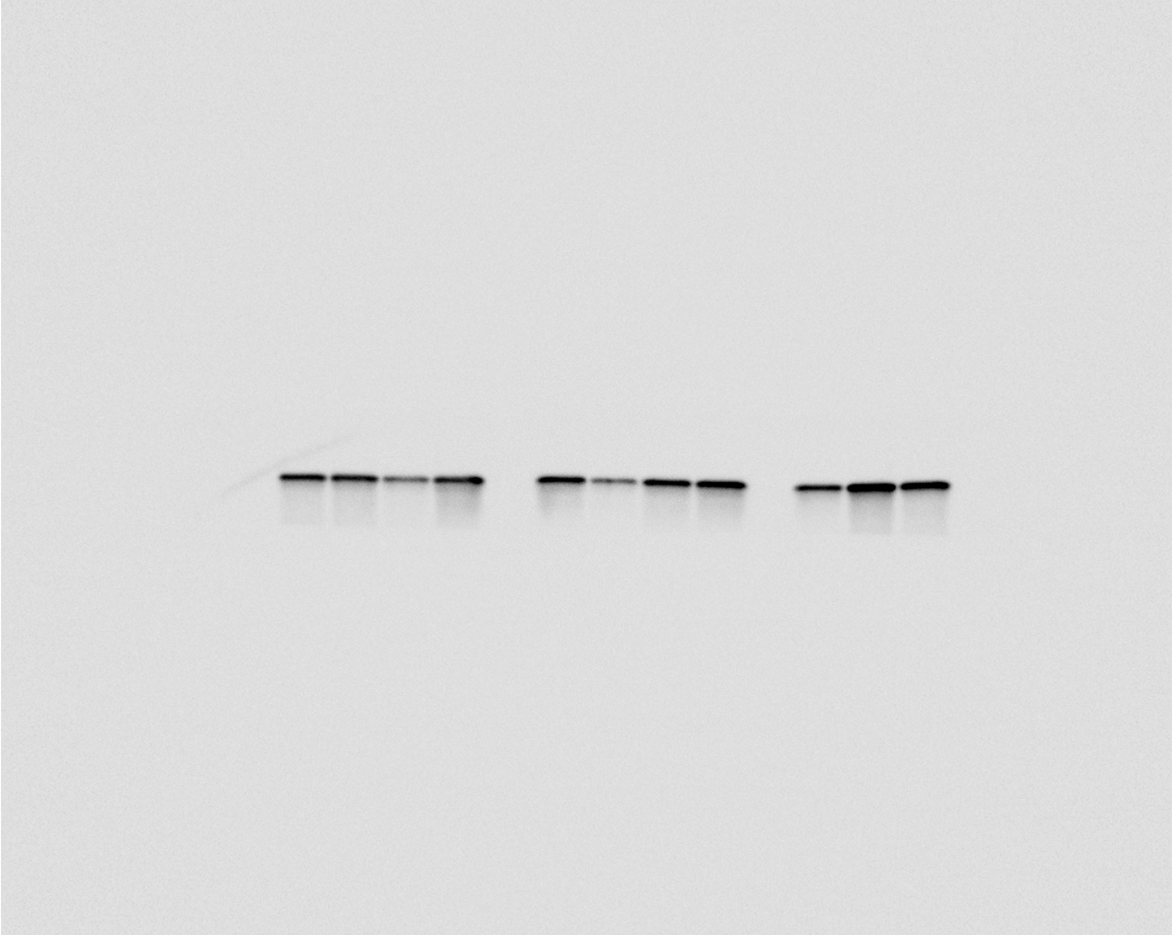

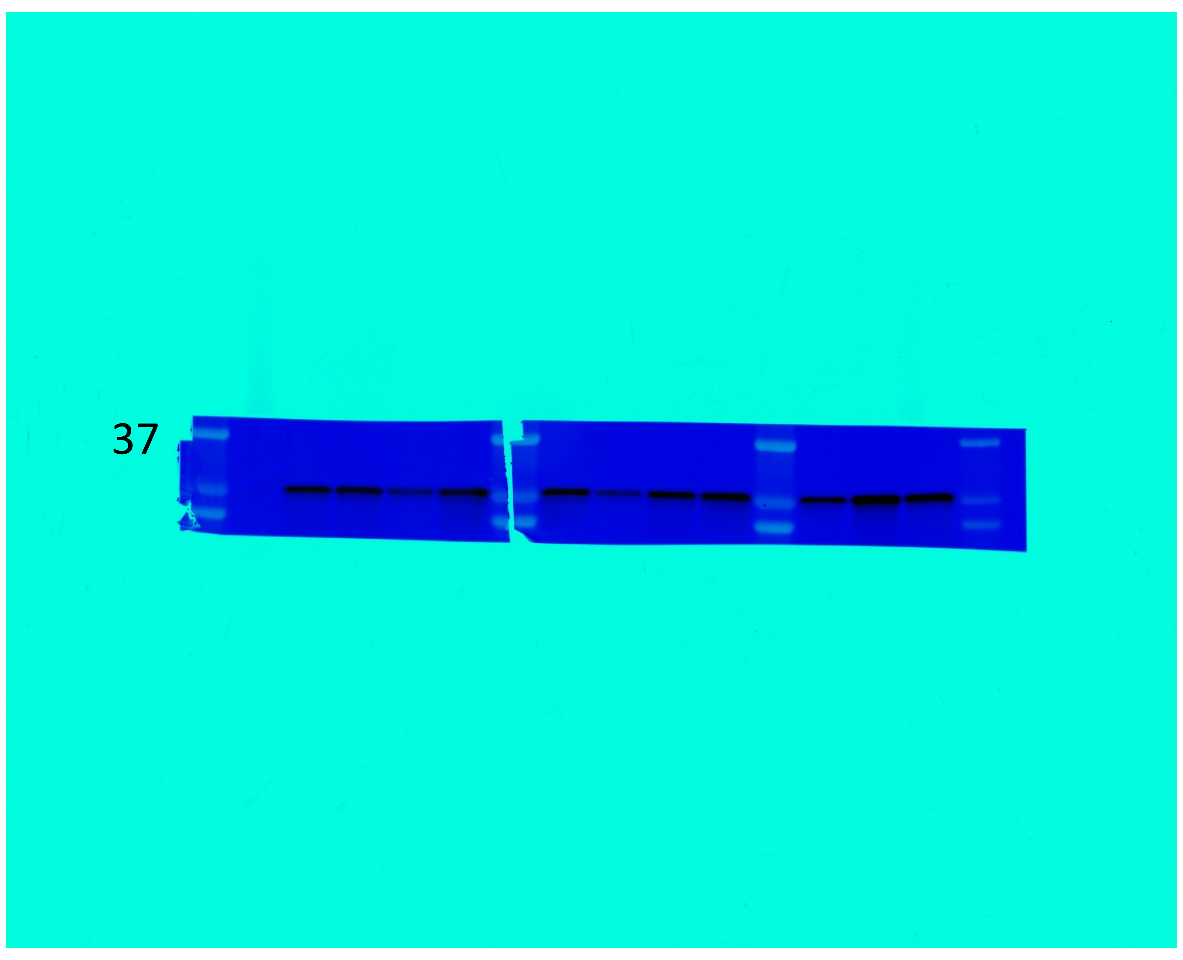

Ponceau S Staining

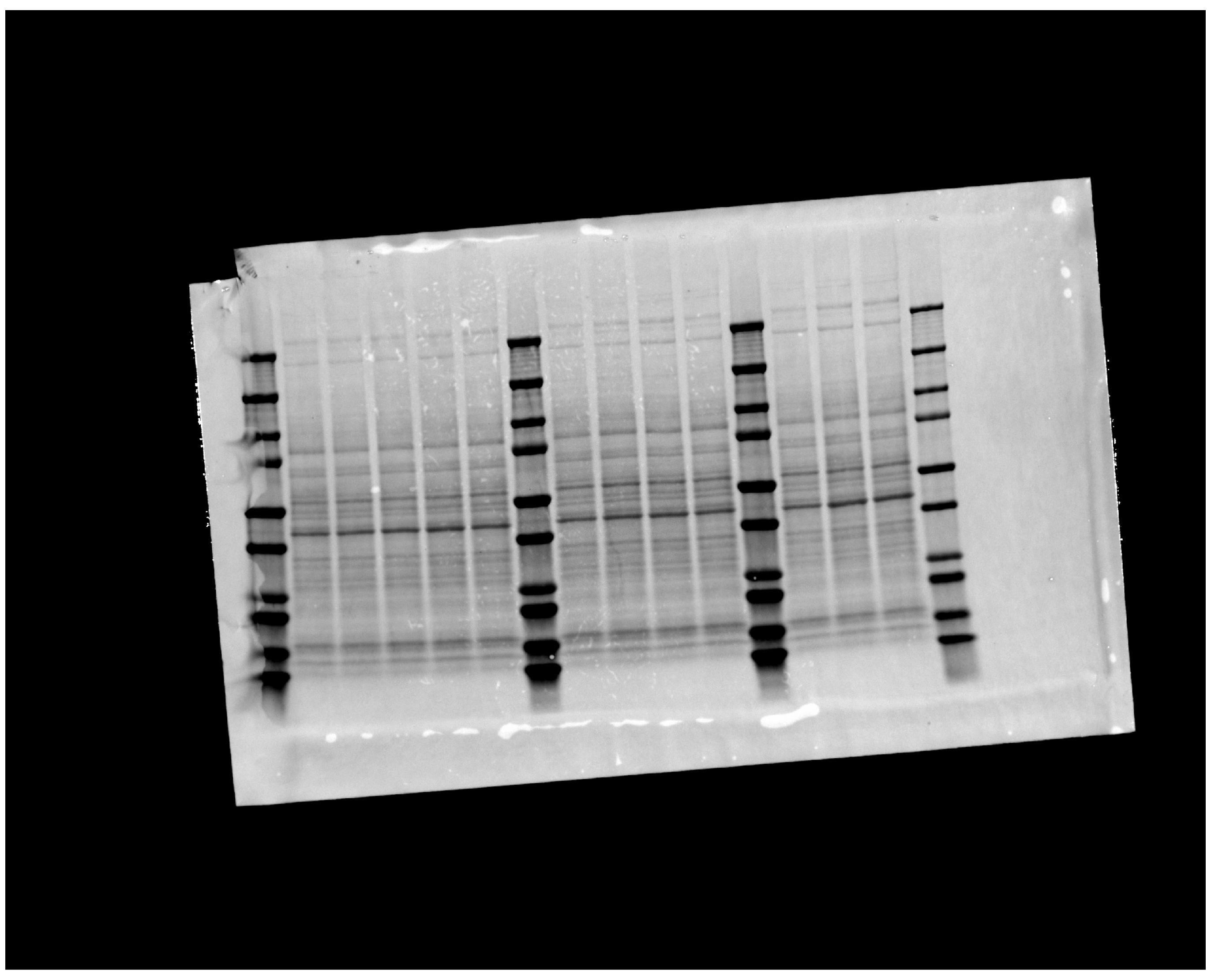

Sample order, left to right:
7 uL ladder
Plain
Bulk EV
EV E7
EV G4
EV G11
7 uL ladder
Bulk CICDUX4
CICDUX4 C9
CICDUX4 E10
CICDUX4 G8
7 uL ladder
Bulk dC1
dC1 C5
dC1 D5
3 uL ladder

Supplemental Figure S3 Panel D

Uncropped Chemiluminescence Image

HSP90

DUX4

ETV5

GFP

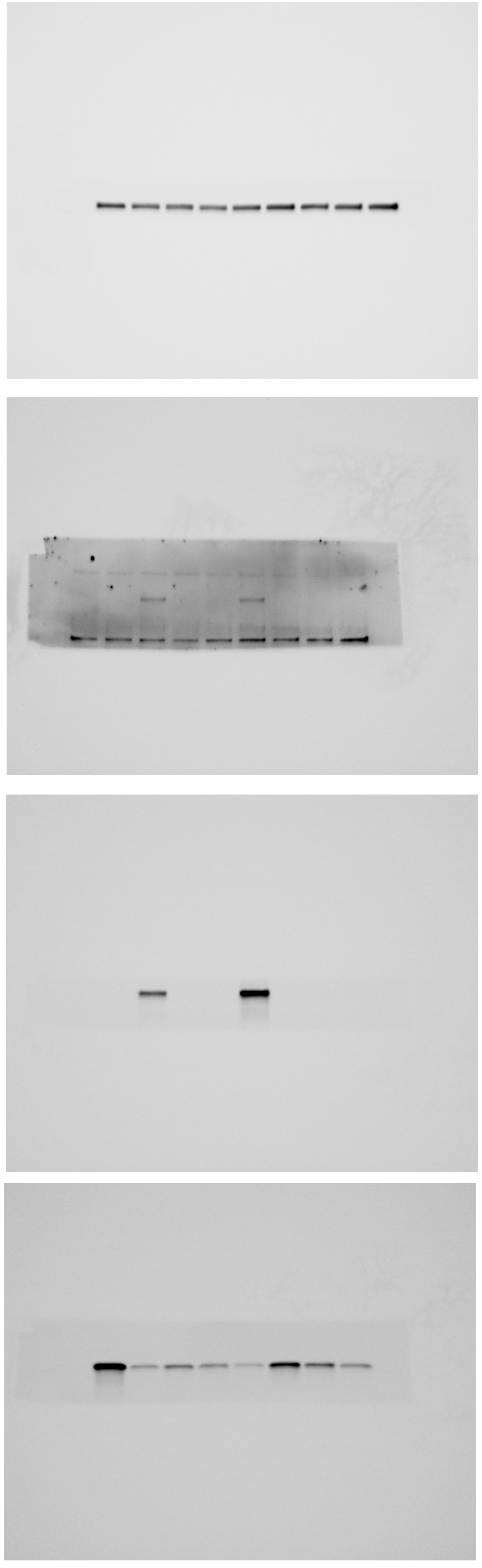

Uncropped Composite (with Colorimetric, for ladder)

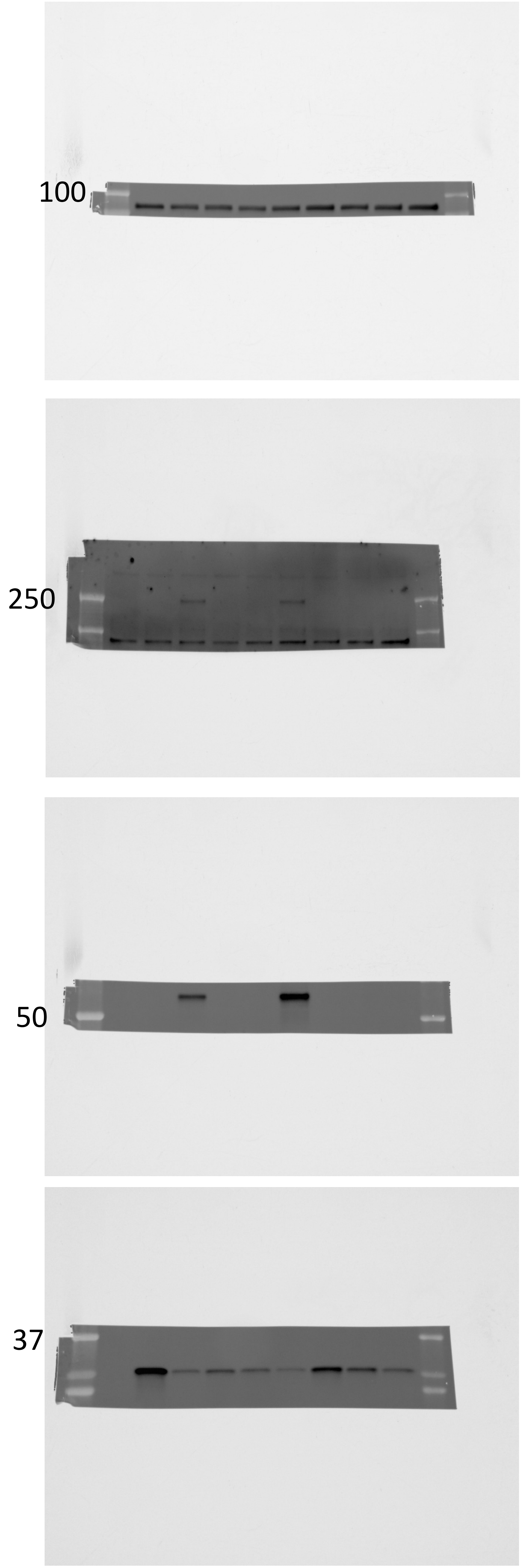

Ponceau S Staining

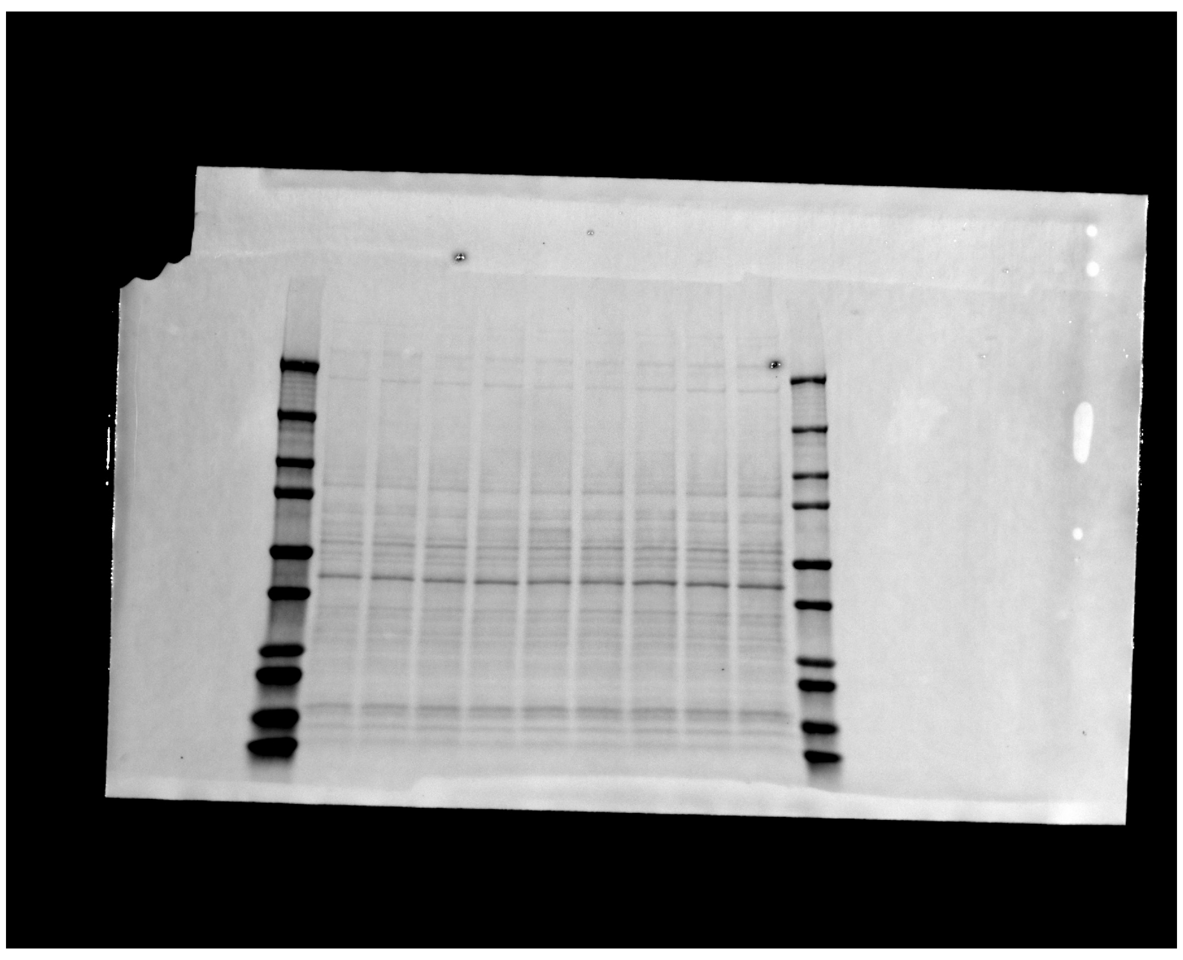

Sample order, left to right:
7 uL ladder
C2C12 plain
C2C12 bulk pMY-EV
C2C12 bulk pMY-CICDUX4
C2C12 pMY-CICDUX4 clone B4
C2C12 pMY-CICDUX4 clone C7.2
C2C12 pMY-CICDUX4 clone D6
C2C12 pMY-CICDUX4 clone E9
C2C12 pMY-CICDUX4 clone F11
C2C12 pMY-CICDUX4 clone G9
3 uL ladder

Supplemental Figure S5 Panel A

Uncropped Chemiluminescence Image

HSP90

DUX4 (short exposure)

DUX4 (long exposure)

ETV5

Myogenin

GFP

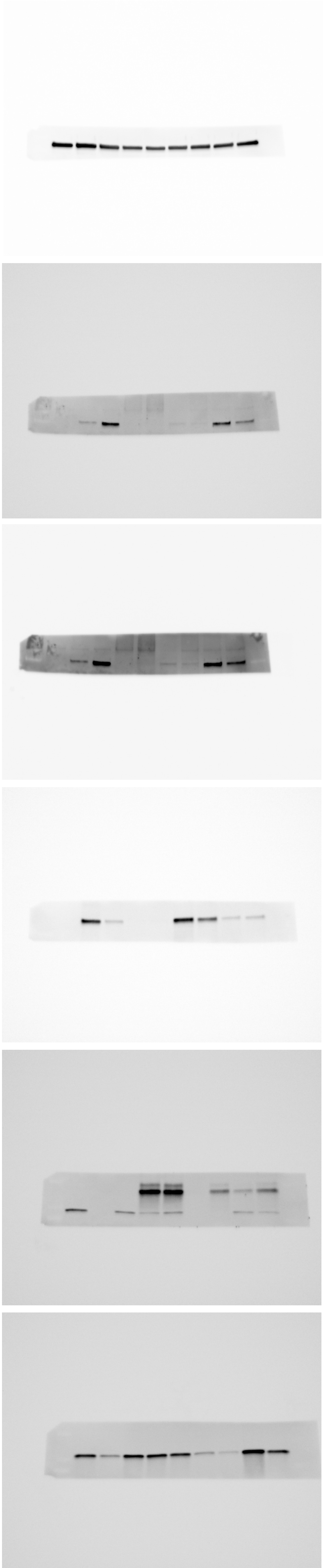

Uncropped Composite (with Colorimetric, for ladder)

100

250

250

50

37

37

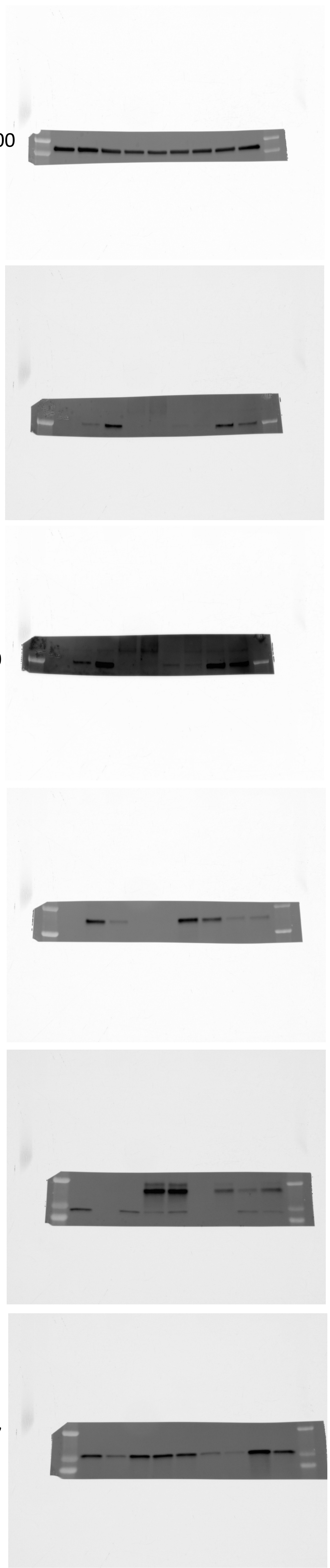

Note: chemi. brightness was adjusted in Image Lab, hence darker bands in the original composite

Ponceau S Staining

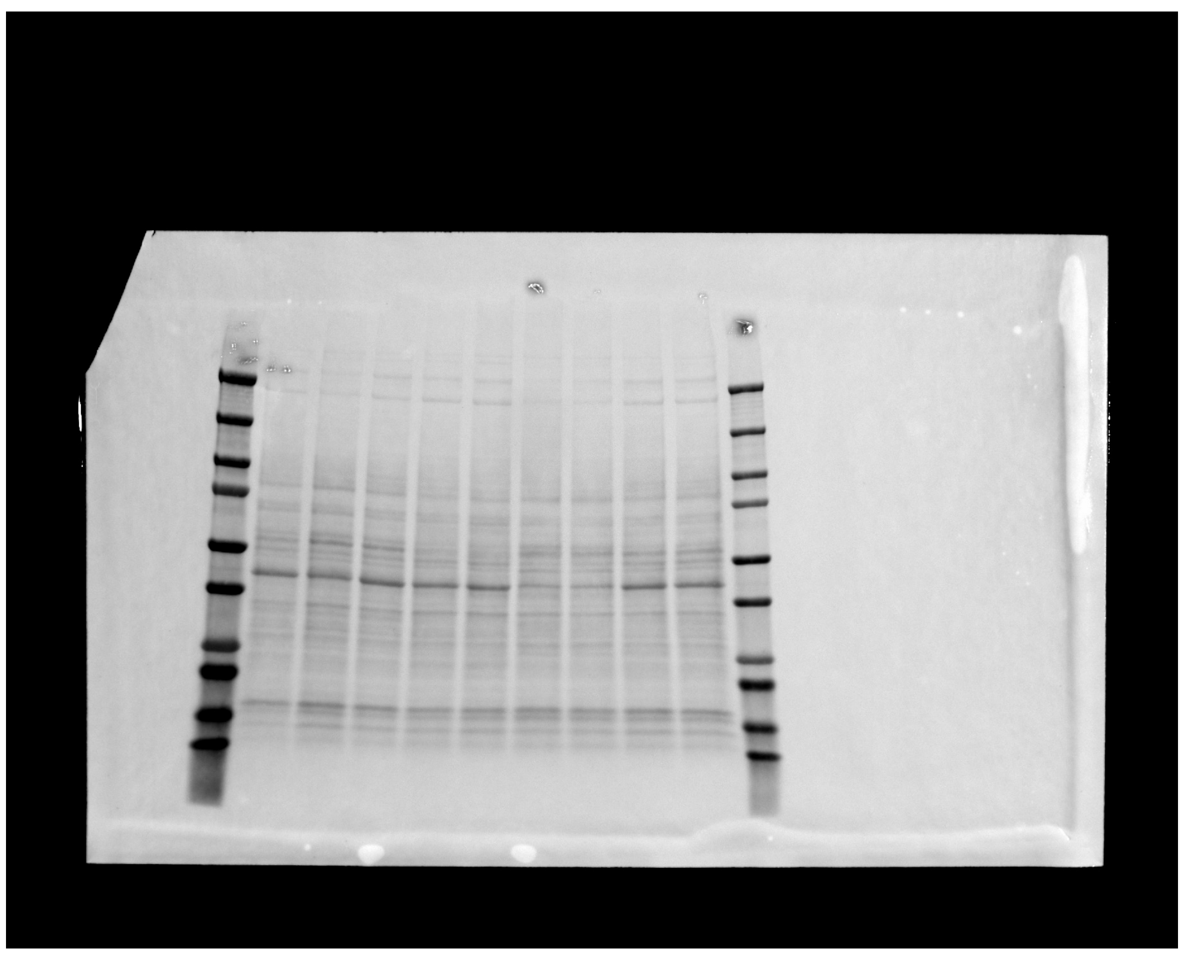

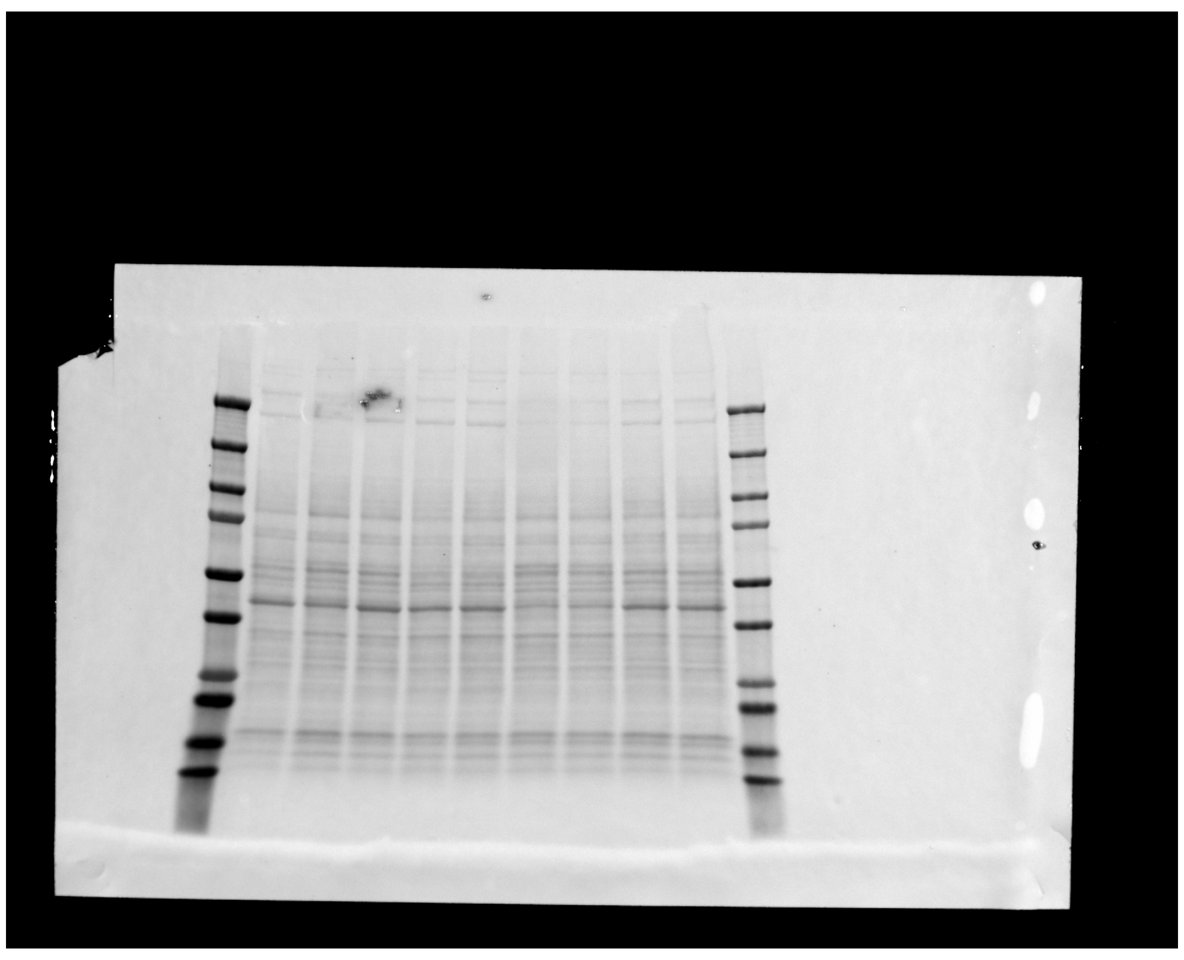

Sample order, left to right:
7 uL ladder
EV G4 day 0
CD4 C9 day 0
dC1 D5 day 0
EV G4 day 5 control
EV G4 day 5 siCIC
CD4 C9 day 5 control
CD4 C9 day 5 siCIC
dC1 D5 day 5 control
dC1 D5 day 5 siCIC
3 uL ladder

Note: ETV5 and GFP cut from the same set of samples (right), and HSP90, DUX4, and Myogenin cut from the other (left)
