# Supplementary material for: The Capicua C1 Domain Is Required for Full Activity of the CIC::DUX4 Fusion Oncoprotein
Source: Cancer Res Commun. 2024 Dec 9;4(12):3099–113. doi: 10.1158/2767-9764.CRC-24-0348 (PMC11626509; doi:10.1158/2767-9764.CRC-24-0348)
Supplement: Supplementary Figure S1 — CIC breakpoints are mildly variable across different 3’ partner genes, and piloting of a CIC::UTR “fusion” model. [file crc-24-0348_supplementary_figure_s1_suppsf1.pdf]

Supp. Fig. S1

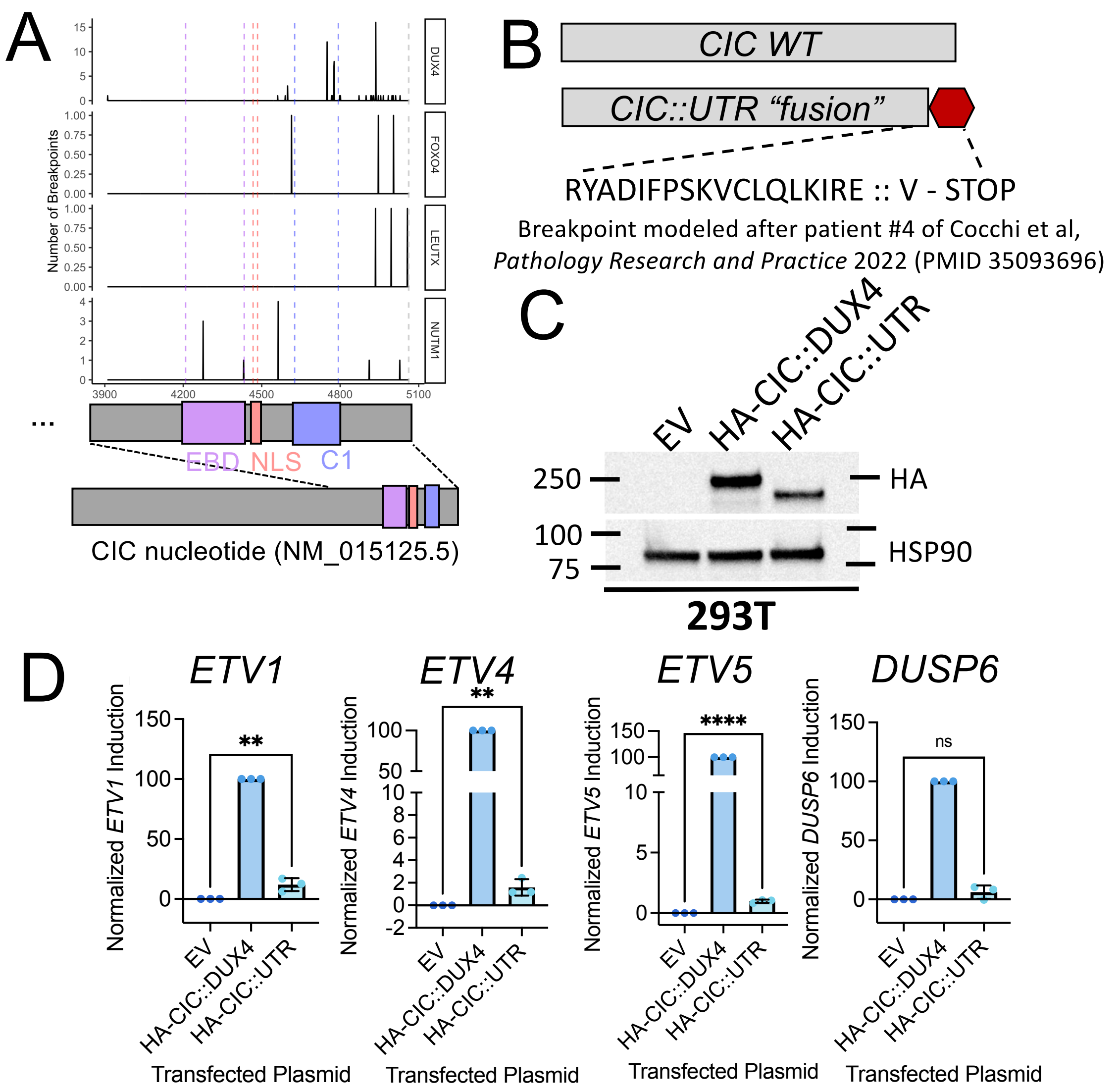

Supplemental Figure S1. *CIC* breakpoints are mildly variable across different 3' partner genes, and piloting of a *CIC*::UTR "fusion" model. (A) Histogram of *CIC* breakpoints across four 3' partner genes for a total of 90 breakpoints using RNA data where both partner breakpoints were available. Breakpoint numbers by partner gene: DUX4 = 74, NUTM1 = 10, LEUTX = 3, FOXO4 = 3. EBD = ERK-binding domain, NLS = nuclear localization signal, C1 = C1 domain. (B) Schematic for design of CIC::UTR construct, WT CIC is shown for visual comparison but HA-CIC::UTR was cloned from an HA-CIC::DUX4 plasmid. (C) Immunoblot of 293T cells approximately 48 hours after transfection with empty vector (EV) or the labeled constructs, data representative of three independent experiments. (D) Normalized RT-qPCR measurement of target gene induction in 293T cells approximately 48 hours after transfection with EV or the labeled constructs. Each data point represents the mean of one of three independent experiments, error bars indicate standard

deviation, **** = $p < 0.0001$, and ** = $p < 0.01$ by one-way ANOVA and Šidák's multiple comparisons test.
