# Supplementary material for: The Capicua C1 Domain Is Required for Full Activity of the CIC::DUX4 Fusion Oncoprotein
Source: Cancer Res Commun. 2024 Dec 9;4(12):3099–113. doi: 10.1158/2767-9764.CRC-24-0348 (PMC11626509; doi:10.1158/2767-9764.CRC-24-0348)
Supplement: Supplementary Figure S2 — Deletion or point mutation of the C1 domain result in attenuated target gene activation and ETV5-promoter engagement by CIC::DUX4. [file crc-24-0348_supplementary_figure_s2_suppsf2.pdf]

Supp. Fig. S2

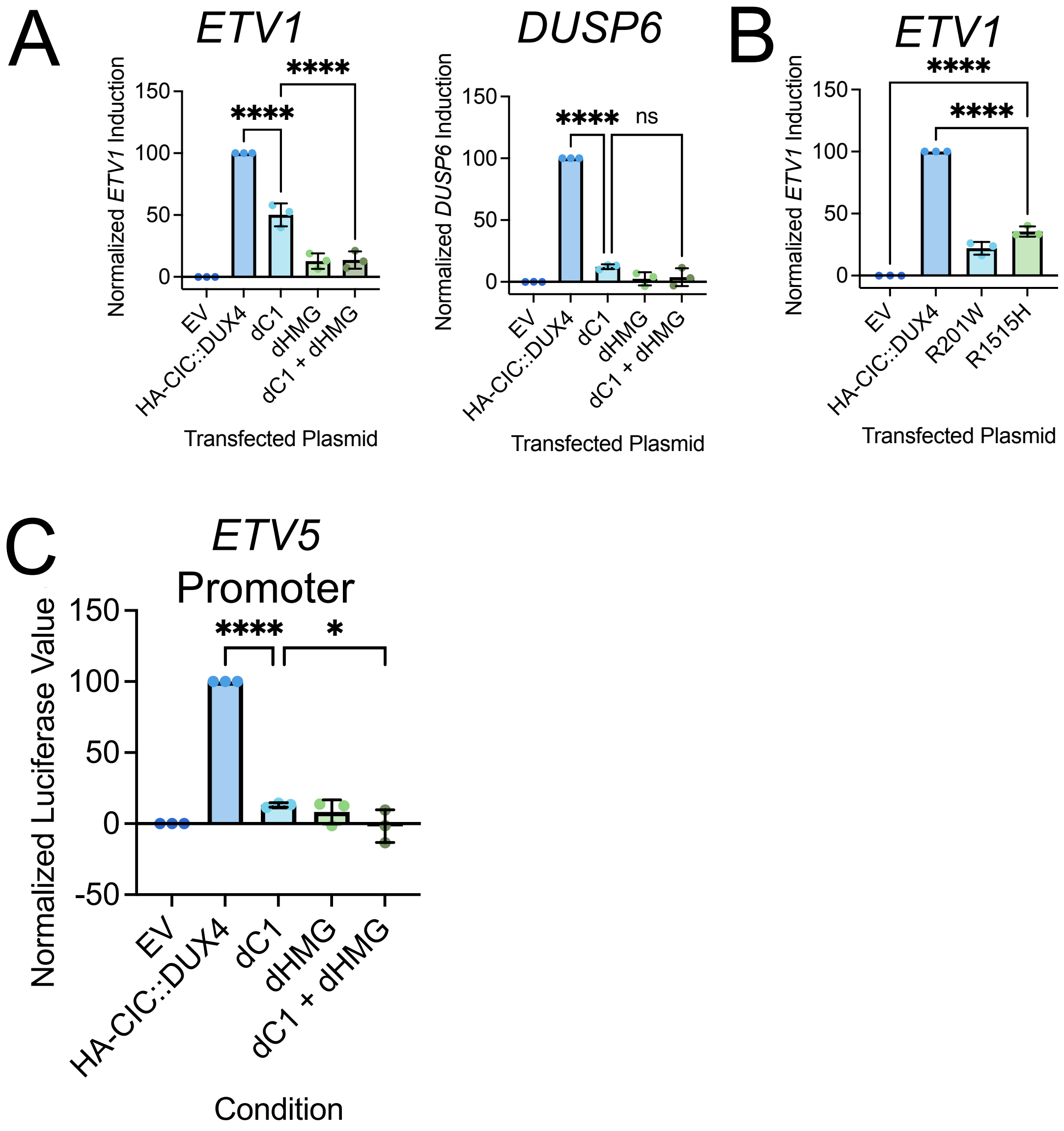

Supplemental Figure S2. Deletion or point mutation of the C1 domain result in attenuated target gene activation and ETV5-promoter engagement by CIC::DUX4. (A and B) Normalized RT-qPCR measurement of target gene induction in 293T cells approximately 48 hours after transfection with EV or the labeled constructs. Each data point represents the mean of one of three independent experiments, error bars indicate standard deviation, **** = $p < 0.0001$, and ns = $p > 0.05$ by one-way ANOVA and Šidák's multiple comparisons test. (C) Normalized luciferase measurement of target gene induction in 293T cells approximately 48 hours after cotransfection with EV or the labeled constructs along with an ETV5-promoter-luciferase reporter. Each data point represents the mean of one of three independent experiments, error bars indicate standard deviation, **** = $p < 0.0001$, and * = $p \leq 0.05$ by one-way ANOVA and Šidák's multiple comparisons test.
