# Supplementary material for: The Capicua C1 Domain Is Required for Full Activity of the CIC::DUX4 Fusion Oncoprotein
Source: Cancer Res Commun. 2024 Dec 9;4(12):3099–113. doi: 10.1158/2767-9764.CRC-24-0348 (PMC11626509; doi:10.1158/2767-9764.CRC-24-0348)
Supplement: Supplementary Figure S3 — Screening and validation of transduced NIH/3T3 and C2C12 clonal cell lines. [file crc-24-0348_supplementary_figure_s3_suppsf3.pdf]

Supp. Fig. S3

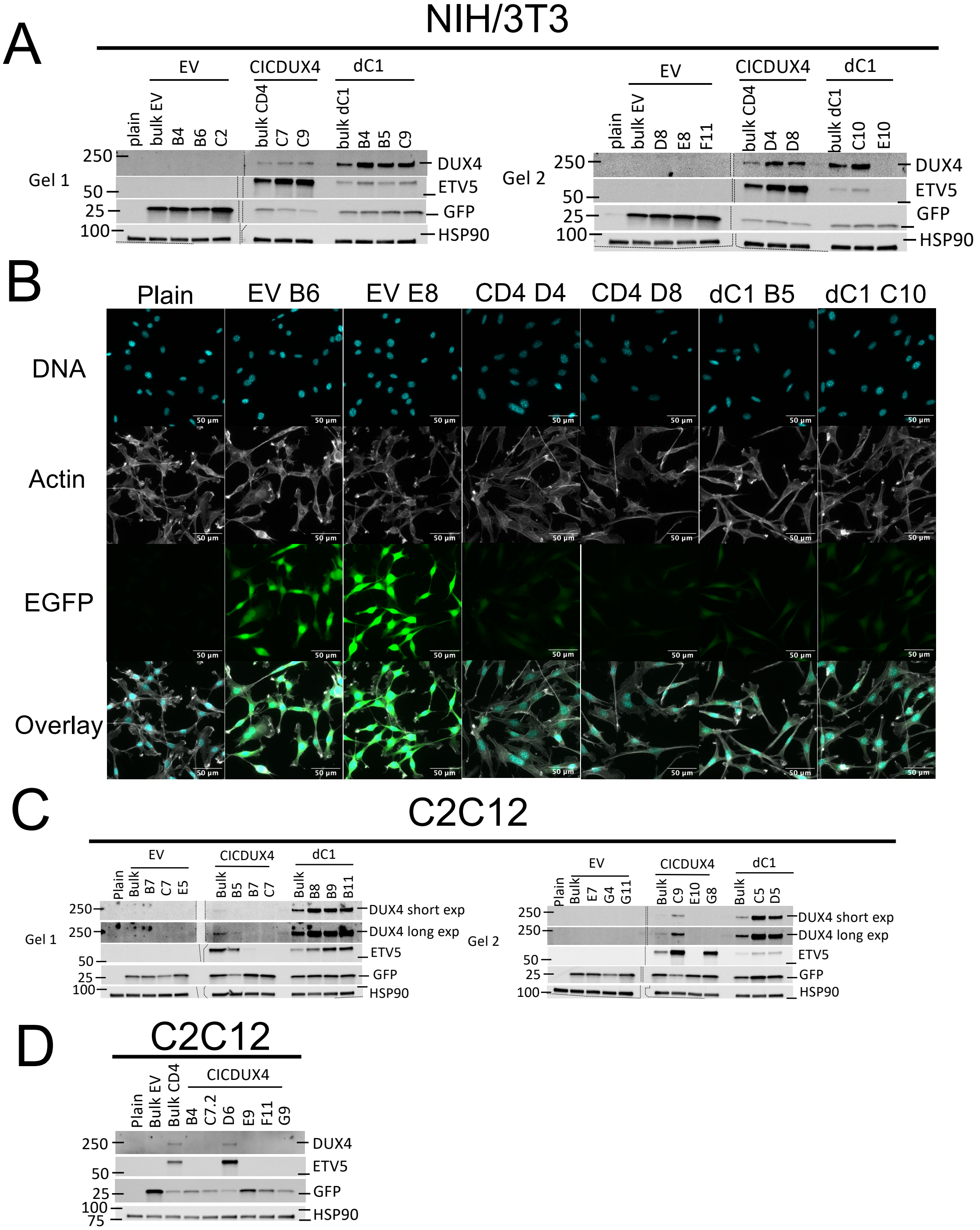

Supplemental Figure S3. Screening and validation of transduced NIH/3T3 and C2C12 clonal cell lines. (A) Immunoblot of untransduced (plain), polyclonal (bulk), or clonal (number-letter) NIH/3T3 cells. Abbreviated labels for transgenes use the same naming scheme as those in Figure 3A. Dashed lines indicate where blots were physically cut due to space limitations. Blank lanes had ladder run in them. This immunoblot for clone screening was performed once. (B) Epifluorescence microscopy of untransduced (plain) or selected clonal NIH/3T3 cells. DNA visualized with DAPI, actin visualized with rhodamine-phalloidin, 20x objective used for imaging,

scale bars indicate 50 μm, representative cells chosen from one experiment. (C) Immunoblot of untransduced (plain), polyclonal (bulk), or clonal (number-letter) C2C12 cells. Abbreviated labels for transgenes use the same naming scheme as those in Figure 3A. “short exp” or “long exp” indicates different exposure times. Dashed lines indicate where blots were physically cut due to space limitations. Blank lanes had ladder run in them. This immunoblot for clone screening was performed once. (D) Immunoblot of untransduced (plain), polyclonal (bulk), or clonal (number-letter) C2C12 cells. Abbreviated labels for transgenes use the same naming scheme as those in Figure 3A. This immunoblot for clone screening was performed once.
