# Supplementary material for: The Capicua C1 Domain Is Required for Full Activity of the CIC::DUX4 Fusion Oncoprotein
Source: Cancer Res Commun. 2024 Dec 9;4(12):3099–113. doi: 10.1158/2767-9764.CRC-24-0348 (PMC11626509; doi:10.1158/2767-9764.CRC-24-0348)
Supplement: Supplementary Figure S4 — Deletion of the C1 domain attenuates activation of CIC::DUX4-induced genes, but varies in magnitude on some known target genes. [file crc-24-0348_supplementary_figure_s4_suppsf4.pdf]

# Supp. Fig. S4

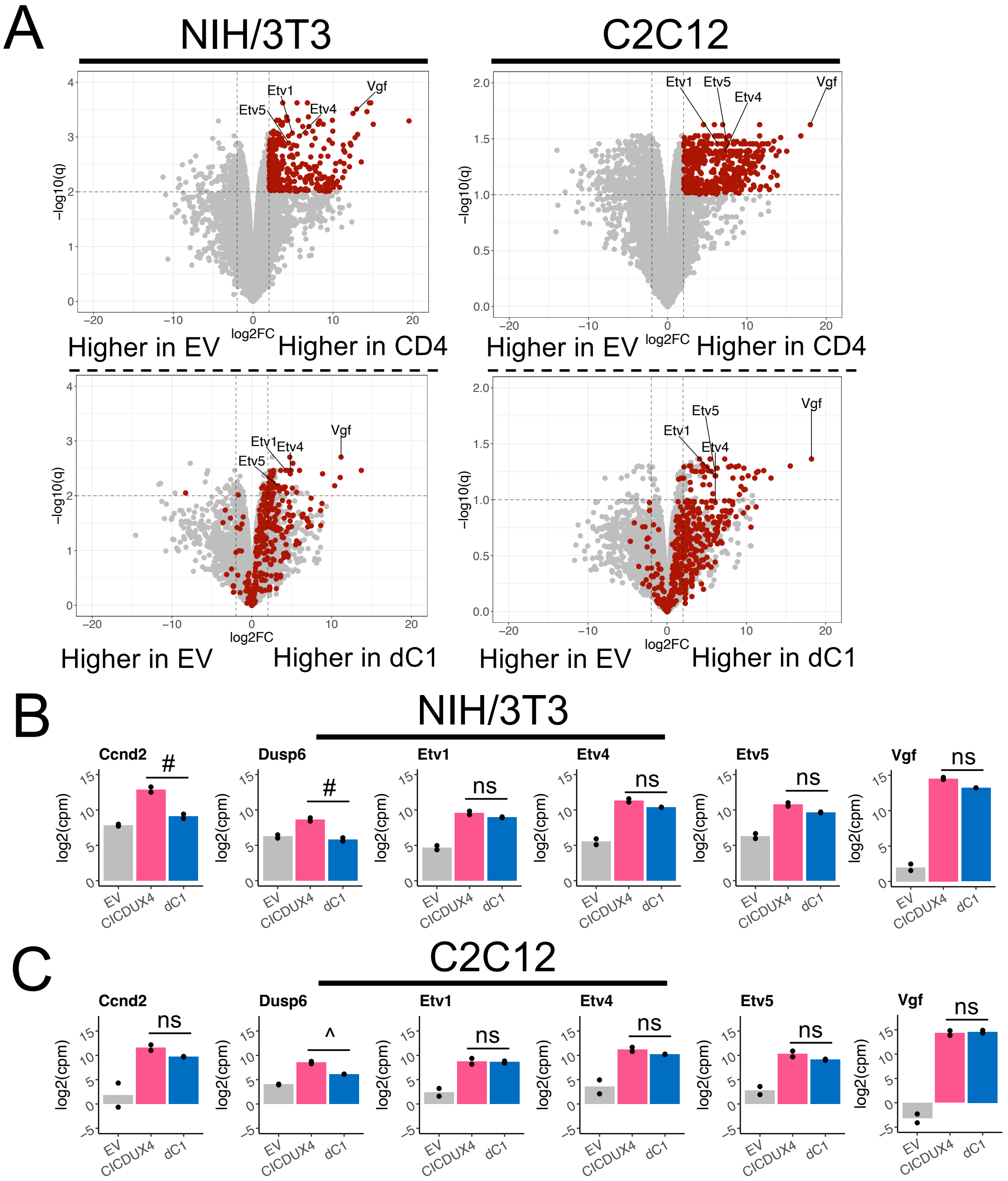


**Supplemental Figure S4.** Deletion of the C1 domain attenuates activation of CIC::DUX4-induced genes, but varies in magnitude on some known target genes. (A) Volcano plots of differentially expressed genes between full-length CIC::DUX4 (CD4) or C1-deleted CIC::DUX4 (dC1) clones and empty vector (EV) clones. NIH/3T3 clone data is shown on the left, C2C12 clone data is shown on the right. Maroon-colored genes were significantly upregulated in the CD4 vs EV comparison (NIH/3T3: $\log_2$ fold change > 2, q < 0.01; C2C12: $\log_2$ fold change > 2, q < 0.1). Select known or high-confidence CIC/CIC::DUX4 target genes are labeled. (B) $\log_2$(counts per million) measurements for six selected known or high-confidence CIC/CIC::DUX4 target genes in NIH/3T3 clones, grouped by transduction. Bars represent mean values, points represent individual clones. In the edgeR differential expression analysis using quasi-likelihood F tests, all

six genes were significantly different for CD4 vs EV (q < 0.01) and only four genes (*Etv1*, *Etv4*, *Etv5*, *Vgf*) were significantly different (q < 0.01) for dC1 vs EV. FDR-adjusted p-values for dC1 vs. CD4 quasi-likelihood F test comparisons are shown, where # indicates that q < 0.01, ns indicates q ≥ 0.01. (C) $\log_2$(counts per million) measurements for six selected known or high-confidence CIC/CIC::DUX4 target genes in C2C12 clones, grouped by transduction. Bars represent mean values, points represent individual clones. In the edgeR differential expression analysis using quasi-likelihood F tests, all six genes were significantly different for CD4 vs EV (q < 0.1) and only four genes (*Dusp6*, *Etv1*, *Etv5*, *Vgf*) were significantly different (q < 0.1) for dC1 vs EV. FDR-adjusted p-values for dC1 vs. CD4 quasi-likelihood F test comparisons are shown, where ^ indicates that q < 0.1, ns indicates q ≥ 0.1.
