# Supplementary material for: The Capicua C1 Domain Is Required for Full Activity of the CIC::DUX4 Fusion Oncoprotein
Source: Cancer Res Commun. 2024 Dec 9;4(12):3099–113. doi: 10.1158/2767-9764.CRC-24-0348 (PMC11626509; doi:10.1158/2767-9764.CRC-24-0348)
Supplement: Supplementary Figure S5 — siCIC treatment can rescue C2C12 clone CIC::DUX4-related differentiation defects, and analysis of myogenic differentiation-related gene expression in C2C12 clones. [file crc-24-0348_supplementary_figure_s5_suppsf5.pdf]

Supp. Fig. S5

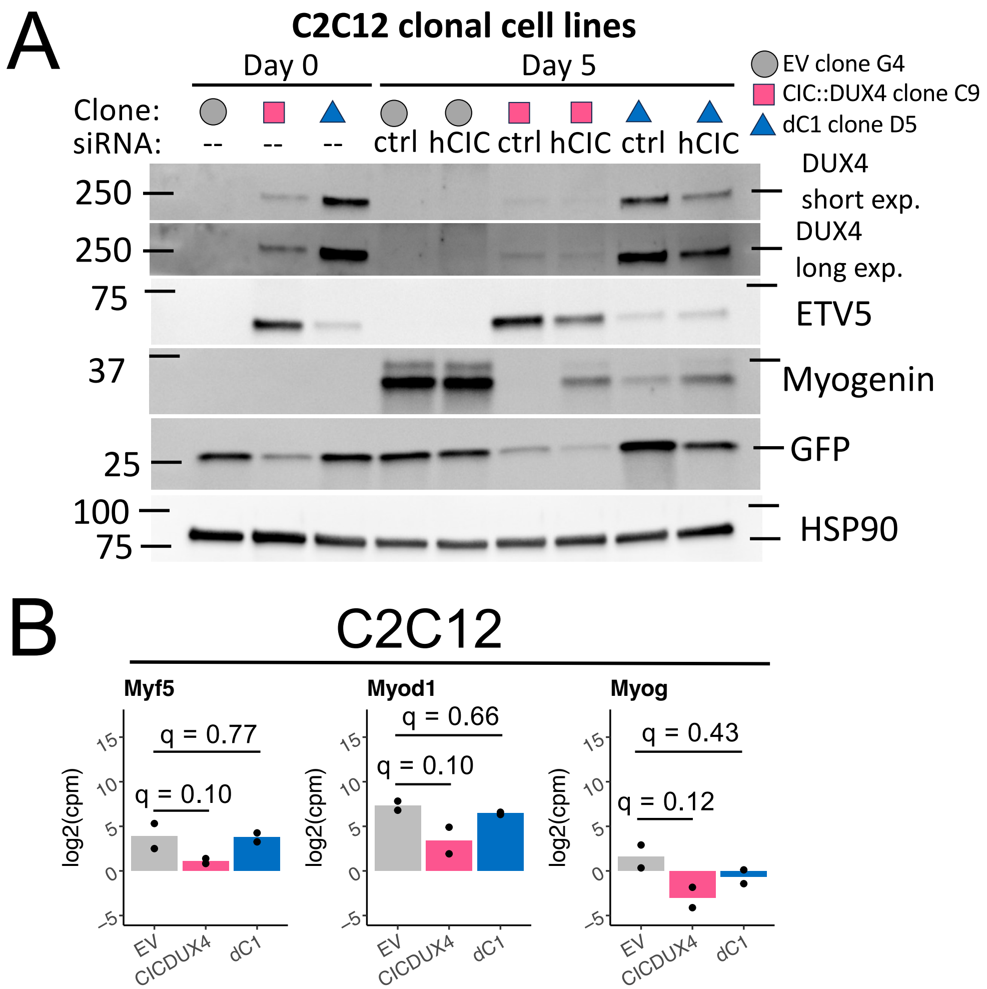

Supplemental Figure S5. siCIC treatment can rescue C2C12 clone CIC::DUX4-related differentiation defects, and analysis of myogenic differentiation-related gene expression in C2C12 clones. (A) Immunoblot of clonal C2C12 cells after differentiation for the indicated times and following transfection with non-targeting (ctrl) or human *CIC* targeting (hCIC) siRNA. Representative of two independent experiments. The HSP90, DUX4, and Myogenin blots were aggregated with GFP and ETV5 blots derived from identically loaded samples processed simultaneously, see Supplemental Dataset S2 for full Ponceau S loading controls and details. (B) log_2(counts per million) measurements for three selected genes related to myogenic differentiation in C2C12 clones, grouped by transduction. Bars represent mean values, points represent individual clones. FDR-corrected p-values (q-values) are shown from edgeR differential expression analysis using quasi-likelihood F tests. The data are derived from the experiment described in Figure 3, meaning that the clones were growing normally and not subjected to differentiation conditions when RNA was harvested.
