# Supplementary material for: The Capicua C1 Domain Is Required for Full Activity of the CIC::DUX4 Fusion Oncoprotein
Source: Cancer Res Commun. 2024 Dec 9;4(12):3099–113. doi: 10.1158/2767-9764.CRC-24-0348 (PMC11626509; doi:10.1158/2767-9764.CRC-24-0348)
Supplement: Supplementary Figure S6 — C1-intact CIC::DUX4 expression alters the growth pattern of clonal C2C12 cells. [file crc-24-0348_supplementary_figure_s6_suppsf6.pdf]

Supp. Fig. S6

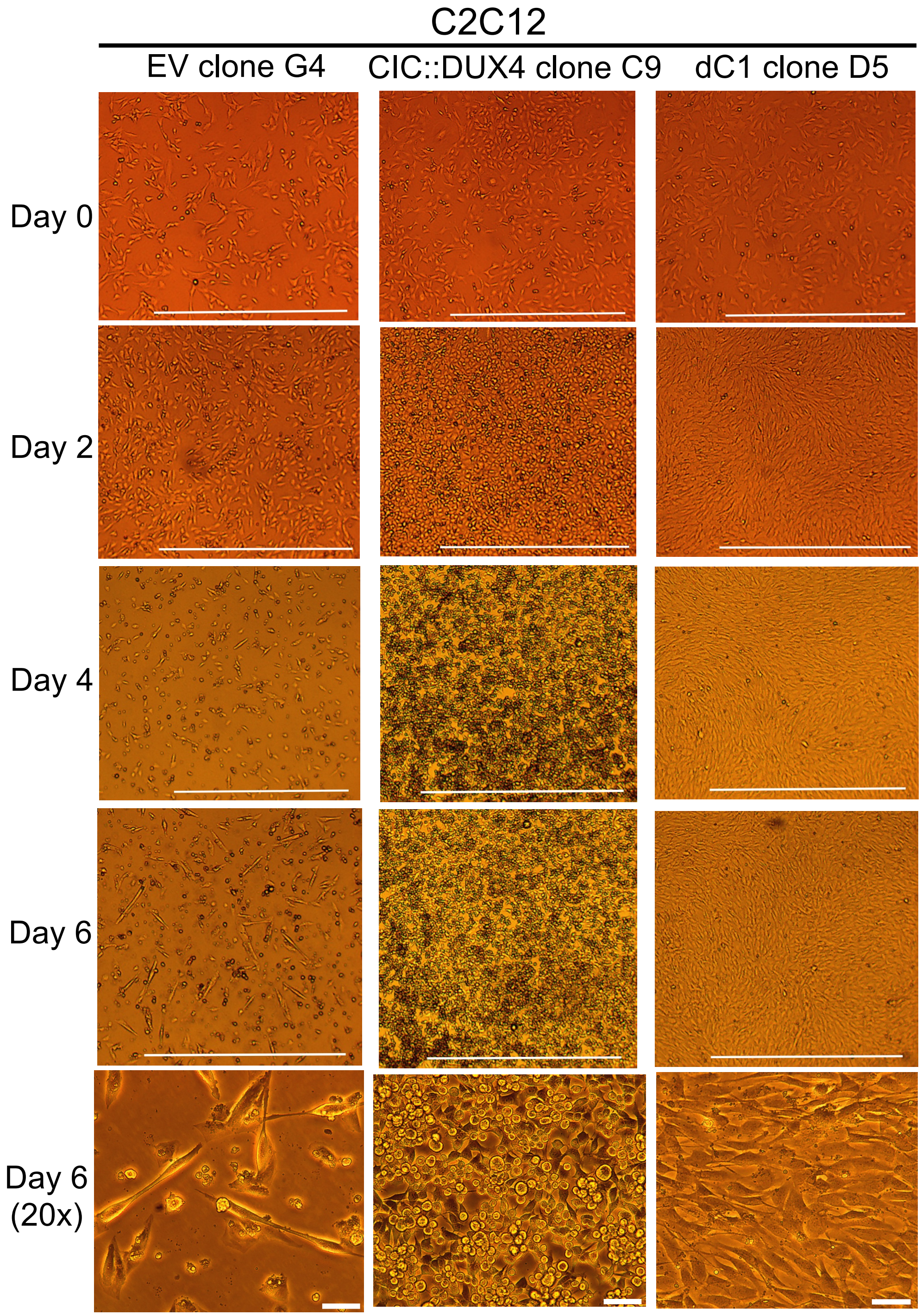

Supplemental Figure S6. C1-intact CIC::DUX4 expression alters the growth pattern of clonal C2C12 cells. Clonal C2C12 cell lines expressing the indicated transduced constructs imaged at the indicated timepoints during a differentiation experiment. Images are from different regions of

wells and/or different identically prepared plates between timepoints. Imaged with a 4x objective unless otherwise indicated, scale bar represents 1mm for 4x objective and 0.05mm scale bar for 20x objective. Representative of two independent experiments.
