# Supplementary material for: The Capicua C1 Domain Is Required for Full Activity of the CIC::DUX4 Fusion Oncoprotein
Source: Cancer Res Commun. 2024 Dec 9;4(12):3099–113. doi: 10.1158/2767-9764.CRC-24-0348 (PMC11626509; doi:10.1158/2767-9764.CRC-24-0348)
Supplement: Supplementary Figure S7 — Full-length or C1-deleted CIC::DUX4 expression alters 3D growth in NIH/3T3 clones. [file crc-24-0348_supplementary_figure_s7_suppsf7.pdf]

Supp. Fig. S7

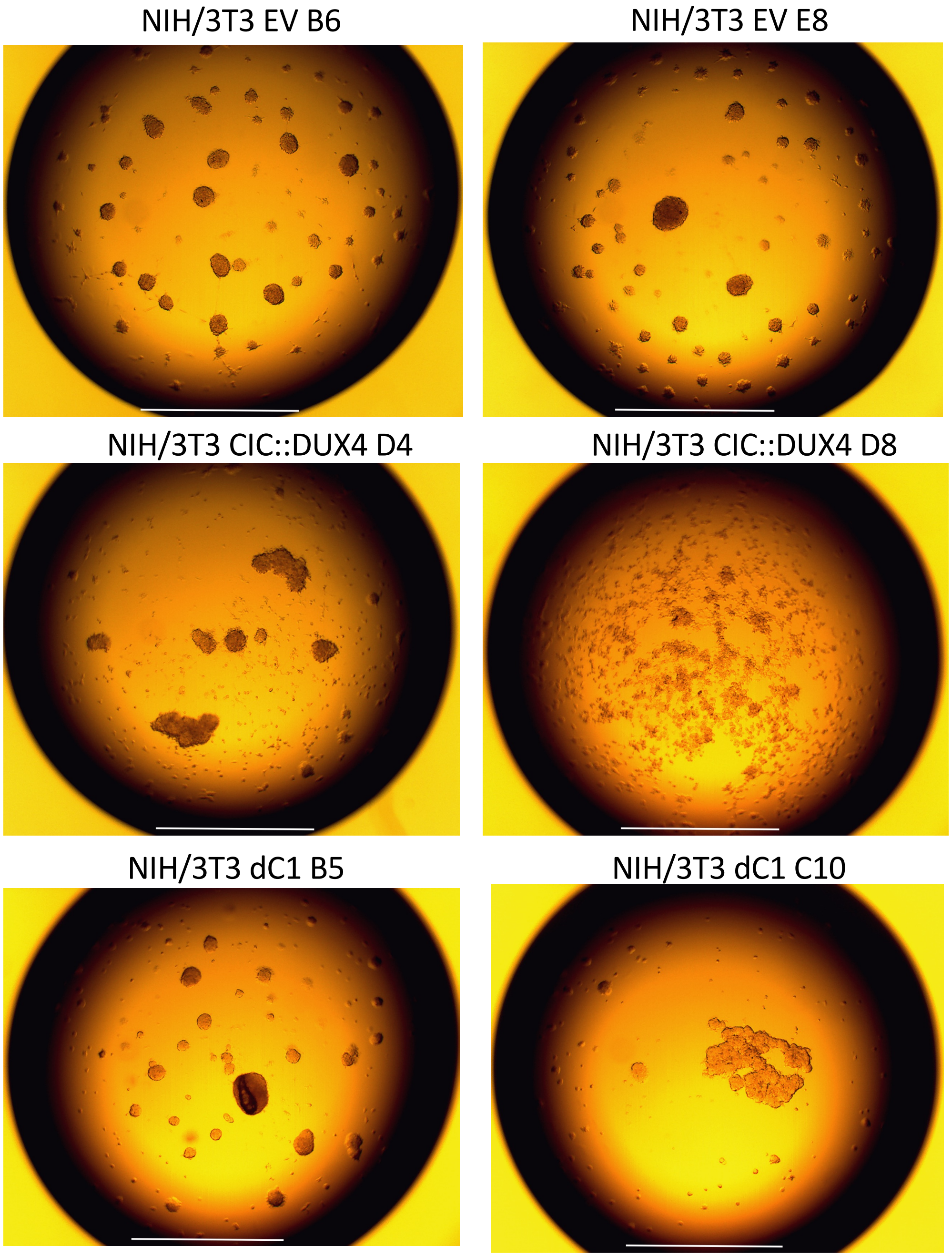

Supplemental Figure S7. Full-length or C1-deleted CIC::DUX4 expression alters 3D growth in NIH/3T3 clones. Images of the indicated NIH/3T3 clones after approximately 24 hours in a hanging drop assay. Imaged with a 4x objective, scale bar represents 1mm. Representative drops are shown from three independent experiments, each with 12 drops per condition.
