# Supplementary material for: The Capicua C1 Domain Is Required for Full Activity of the CIC::DUX4 Fusion Oncoprotein
Source: Cancer Res Commun. 2024 Dec 9;4(12):3099–113. doi: 10.1158/2767-9764.CRC-24-0348 (PMC11626509; doi:10.1158/2767-9764.CRC-24-0348)
Supplement: Supplementary Figure S8 — Growth dynamics of clonal NIH/3T3 and C2C12 cell lines transduced with empty vector, full length CIC::DUX4, or C1-deleted CIC::DUX4. [file crc-24-0348_supplementary_figure_s8_suppsf8.pdf]

Supp. Fig. S8

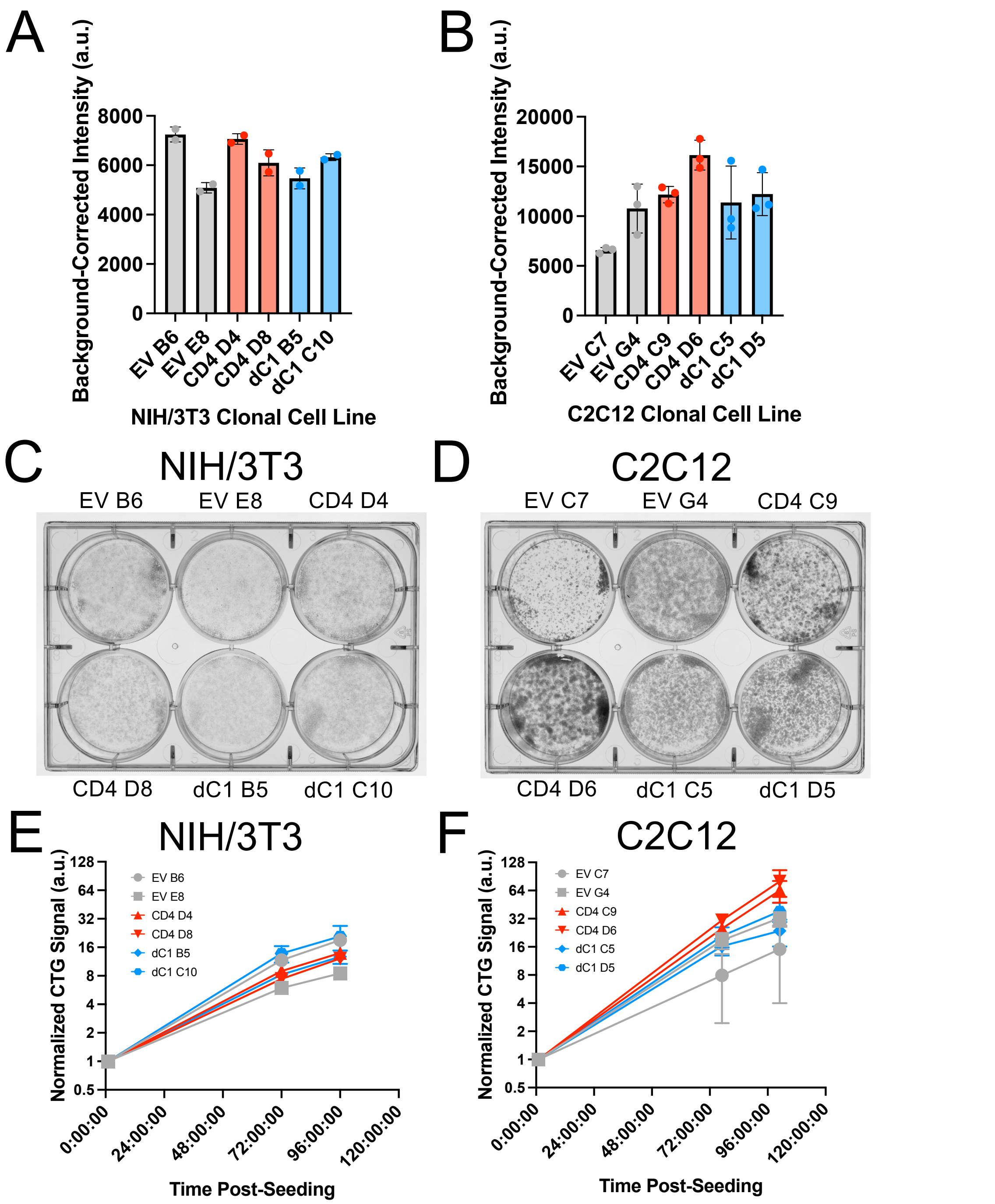

Supplemental Figure S8. Growth dynamics of clonal NIH/3T3 and C2C12 cell lines transduced with empty vector, full length CIC::DUX4, or C1-deleted CIC::DUX4. (A) Quantitation of crystal violet signal from NIH/3T3 clones seeded with 10,000 cells and allowed to grow for approximately 96 hours. Clone name prefixes refer to transductions: EV = empty vector, CD4 = full-length CIC::DUX4, dC1 = C1-deleted CIC::DUX4. Each point represents data from one of two independent experiments, error bars indicate standard deviation. (B) Quantitation of crystal violet

signal from C2C12 clones seeded with 7,500 cells and allowed to grow for approximately 96 hours. Clone name prefixes are the same as above. Each point represents data from one of three independent experiments, error bars indicate standard deviation. (C) A representative NIH/3T3 clone crystal violet-stained plate from the two experiments performed in (A). (D) A representative C2C12 clone crystal violet-stained plate from the three experiments performed in (B). (E) Quantitation of CellTiter-Glo signal from 350 cells of NIH/3T3 clones seeded and measured at the indicated timepoints (hh:mm:ss), with signal normalized to the 1-hour timepoint. Independent experiments were performed twice in technical triplicate, with normalized technical replicate data averaged within each experiment. Data points indicate the mean of the two independent experiments, error bars represent standard deviation. (F) Quantitation of CellTiter-Glo signal from 350 cells of C2C12 clones seeded and measured at the indicated timepoints (hh:mm:ss), with signal normalized to the 1-hour timepoint. Independent experiments were performed twice in technical triplicate, with normalized technical replicate data averaged within each experiment. Data points indicate the mean of the two independent experiments, error bars represent standard deviation.
