# Supplementary material for: The Capicua C1 Domain Is Required for Full Activity of the CIC::DUX4 Fusion Oncoprotein
Source: Cancer Res Commun. 2024 Dec 9;4(12):3099–113. doi: 10.1158/2767-9764.CRC-24-0348 (PMC11626509; doi:10.1158/2767-9764.CRC-24-0348)
Supplement: Supplementary Figure S9 — Only full-length CIC::DUX4 expressing C2C12 cells are capable of forming overt tumors in a nude mouse subcutaneous injection model. [file crc-24-0348_supplementary_figure_s9_suppsf9.pdf]

Supp. Fig. S9

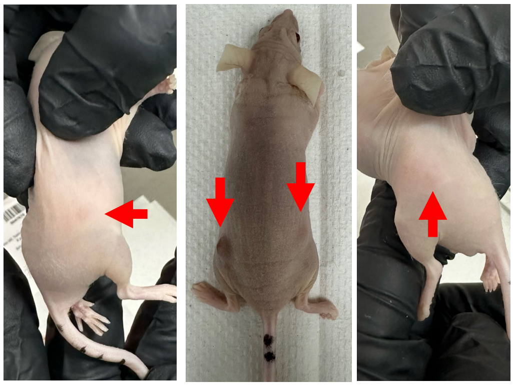

Supplemental Figure S9. Only full-length CIC::DUX4 expressing C2C12 cells are capable of forming overt tumors in a nude mouse subcutaneous injection model. Images of representative lesion-bearing mice injected with the indicated C2C12 clones (top text, M# indicates mouse number in the group) at the time of sacrifice (bottom text). Red arrows indicate lesion sites.
